# Tumor‐Associated Macrophages as Therapeutic Targets: Deciphering Interaction Networks and Advancing Clinical Translation

**DOI:** 10.1002/mco2.70599

**Published:** 2026-01-28

**Authors:** Wurihan Bao, Xiaojie Qu, Yiqi Wang, Dan Huang, Huiling Zhang, Mingyuan Dong, Han Sun, Zhaogang Yang, Xuefeng Li

**Affiliations:** ^1^ School of Life Sciences Jilin University Changchun China; ^2^ Cancer Center The First Hospital of Jilin University Changchun China; ^3^ Department of Breast Surgery General Surgery Center The First Hospital of Jilin University Changchun China

**Keywords:** immunotherapy, immune escape, tumor‐associated macrophages, tumor microenvironment

## Abstract

Tumor‐associated macrophages (TAMs) represent the most abundant immune cell population within the tumor microenvironment and are central drivers of malignant progression and treatment resistance. High TAMs infiltration in solid tumors consistently correlates with poor clinical outcomes, largely due to their role in establishing an immunosuppressive milieu that supports tumor growth, metastasis, and undermines the efficacy of chemotherapy, radiotherapy (RT), and immune checkpoint inhibitors. Although TAMs are well‐recognized promoters of tumor progression, the development of effective strategies to therapeutically target them remains an unmet clinical need. In this review, we examine the multifaceted mechanisms through which TAMs contribute to malignancy, including phagocytic signaling modulation, metabolic reprogramming, exosomal communication, and crosstalk with other immune cells. We also evaluate three key therapeutic strategies: blocking TAMs recruitment and survival, reprogramming TAMs toward antitumor phenotypes, and the emerging approach of chimeric antigen receptor macrophage therapy. Furthermore, we highlight the synergistic potential of integrating TAMs‐targeted strategies with conventional chemotherapy, RT, and immunotherapeutic approaches. By synthesizing current clinical evidence, this review aims to inform the rational design of next‐generation TAMs‐targeted interventions and to propose novel strategies for overcoming treatment resistance.

## Graphical abstract

Tumor‐associated macrophages are the most abundant immune cells in the tumor microenvironment, driving malignant progression and treatment resistance. This review summarizes the protumor mechanisms of TAMs (including phagocytosis modulation, metabolic reprogramming, exosomal communication, and immune interactions), evaluates three major strategies (blocking TAMs recruitment and survival, phenotype reprogramming, and CAR‐M therapy), and highlights the synergistic potential of combining these approaches with conventional treatments. The aim is to provide a foundation for next‐generation targeted interventions and explore new strategies to overcome treatment resistance.

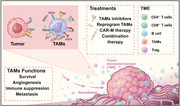

## 1 Introduction

The tumor microenvironment (TME) is a complex, interactive network composed of stromal cells, vasculature, and extracellular matrix (ECM) that plays a critical role in driving oncogenesis, including tumor growth, metastasis, and tissue invasion [1, 2]. Macrophages are key regulators of tissue homeostasis, functioning through signal recognition, phagocytosis, and immune modulation. While typically constituting only 1–5% of cells in healthy tissues, macrophages can expand to represent up to 40% of the cellular composition within the TME [3, 4, 5].

**FIGURE 1:**
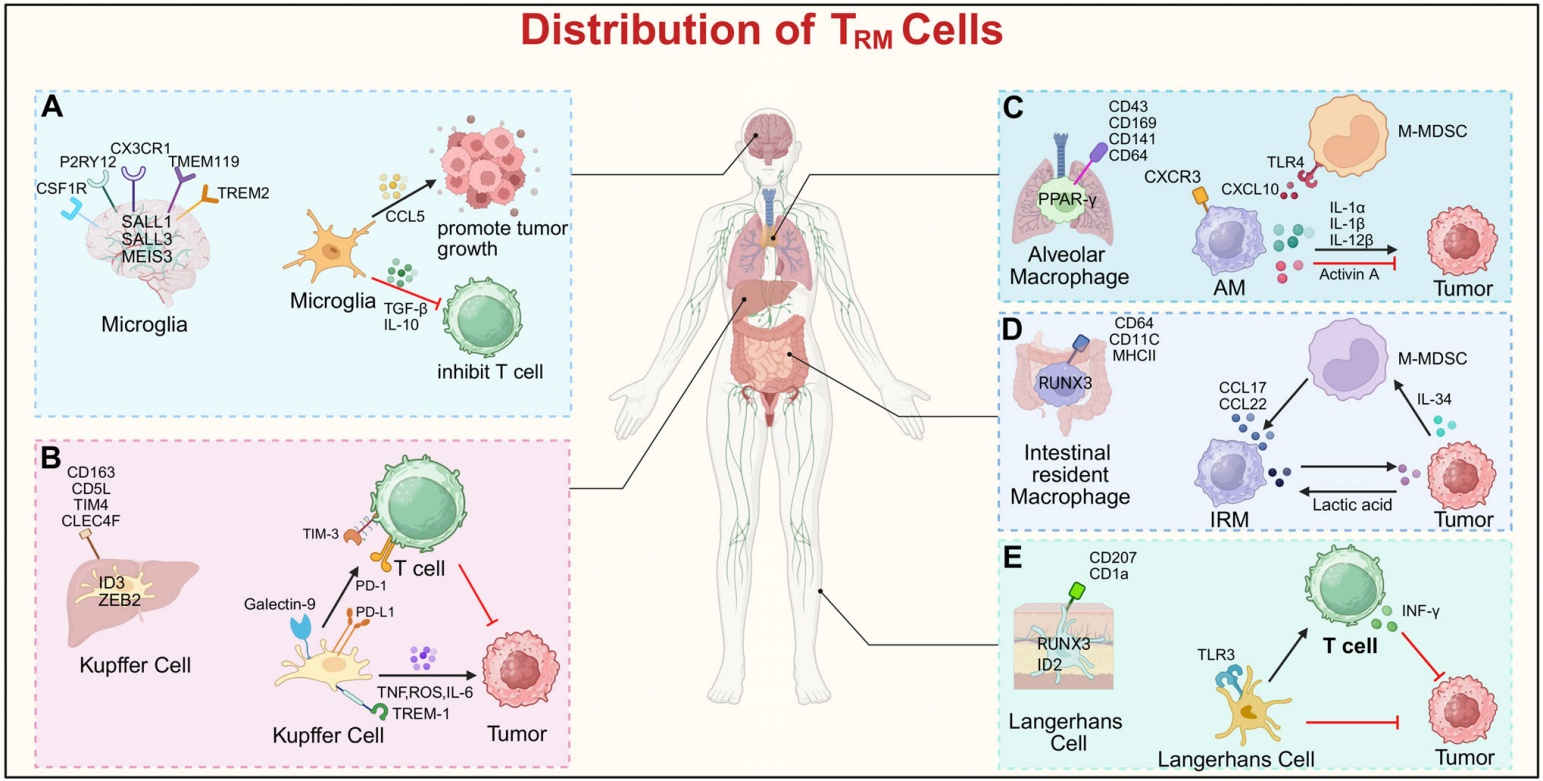
The identity of TRMs is determined by their specific niche of residence. Depending on their location, such as in the lung, liver, brain, skin, or intestine, TRMs release distinct sets of cytokines and display specific surface markers, which together establish their tissue‐specific identity. (A) In tumors, microglia enhance the proliferation and migration of cancer cells in both in vitro and in vivo settings by releasing TGF‐β. Cancer cells induce microglia to secrete and release IL‐6 through the CCL2/CCR2 axis, thereby promoting glioma invasion. During cancer metastasis, tumor cells stimulate the JAK2/STAT3 signaling pathway in microglia by secreting IL‐6 and promote tumor cell migration. (B) In tumor, Kupffer cells (KCs) suppress antitumor immunity through activating the PD‐L1/PD‐1 and galectin‐9/TIM‐3 pathways in T cells. Exposure to cancer cell signals upregulates TREM‐1 expression on KCs, which drives the advancement of HCC. Conversely, loss of TREM‐1 function decreases KC secretion of IL‐1β, IL‐6, CCL2, and CXCL10, resulting in attenuated HCC development. Additionally, KCs‐derived ROS and TNF further contribute to tumor cell expansion. (C) In the lung, alveolar macrophages (AMs) produce activin A, a factor that suppresses the growth of lung cancer cells. These AMs also show increased expression of proinflammatory genes including IL‐12β, IL‐1α, and IL‐1β. In the context of cancer metastasis, AMs can be derived from monocytic myeloid‐derived suppressor cells (M‐MDSCs) through the CXCL10–CXCR3 and TLR4–CCL12 signaling pathways, and they promote metastatic spread through activation of the Wnt/β‐catenin/TNF‐α axis. (D) Tumor‐derived lactic acid, regulated by HIF‐1α, stimulates the expression of VEGF and arginase‐1 in intestinal‐resident macrophages (IRM). Additionally, cancer cell‐secreted IL‐34 drives monocytes to differentiate into M2‐polarized macrophages, which in turn further promotes tumor growth and metastasis. (E) Langerhans cells (LCs) demonstrate antitumor activity. LCs promote T cell proliferation and increase IFN‐γ production. Their capacity to activate T cell responses is further strengthened upon TLR‐3 stimulation.

The understanding of macrophage biology has evolved through several pivotal milestones. The field began with Elie Metchnikoff's identification of “phagocytes” in 1882 [6]. By 1887, Metchnikoff had classified phagocytes into two distinct populations: macrophages and microphages (contemporaneously identified as neutrophils) [7, 8]. This was followed by the proposal and refinement of early conceptual frameworks for the mononuclear phagocyte system [8, 9]. A paradigm shift occurred in 1968 with van Furth's redefinition, which established the bone marrow (BM)‐derived origin of macrophages and dominated the field for decades [9]. The most fundamental revision emerged between the 1990s and 2010s, with the identification of a distinct, embryonically derived, self‐renewing lineage of tissue‐resident macrophages (TRMs), overturning prior dogmas and highlighting their essential role in organ homeostasis [10, 11, 12]. Current classification divides tumor‐associated macrophages (TAMs) into two principal lineages: (i) TRMs, which derive from long‐lived embryonic progenitors (yolk sac or fetal liver origin) during organogenesis, and (ii) BM‐derived macrophages (BMDMs), recruited from circulating myeloid precursors [13, 14, 15]. TRMs constitutively inhabit all healthy organs, where they function as crucial mediators of host defense, tissue homeostasis, structural integrity, and repair mechanisms [16]. In adult mice, most resident macrophage populations, microglia in the central nervous system, Kupffer cells in the liver, and Langerhans cells in the skin, are maintained through local proliferation and do not require replenishment from BM‐derived precursors under homeostatic conditions [10] (Figure 1). Conversely, peritoneal and pancreatic stromal TRMs demonstrate a hybrid maintenance paradigm, integrating self‐renewal capacity with limited BM‐derived supplementation [3]. Notably, the intestinal macrophage population is continuously and entirely replenished by BMDMs, marking a unique exception [17].

In developing tumors, TRMs constitute the initial macrophage population encountering tumor‐derived factors and microenvironmental cues. These cells initiate early inflammatory responses, promote the recruitment of BMDMs, and participate in TAMs formation. Importantly, their contribution to tumor progression is organ specific. Furthermore, TRMs preferentially accumulate in peritumoral regions, where they orchestrate regulatory T‐cell (Tregs) responses, facilitate immune evasion, and promote epithelial–mesenchymal transition (EMT), thereby enhancing tumor cell invasion and metastasis. Concurrently, circulating hematopoietic stem cells secrete cytokines and chemotactic factors, including interleukin (IL)‐1β, C‐C motif ligand (CCL) 2, vascular endothelial growth factor (VEGF), and stromal cell‐derived factor‐1α (SDF‐1α), which mediate the recruitment of monocyte‐derived macrophages into the TME [18]. These recruited monocytes subsequently differentiate into TAMs under microenvironmental cues. TAMs consequently occupy a dynamic phenotypic continuum, demonstrating plastic functional properties that coevolve with tumor progression. At present, the “M1 versus M2” hypothesis, first proposed by Albert Mantovani, remains the prevailing model for classifying TAMs heterogeneity [19]. This framework provides a useful conceptual tool for understanding the dual roles of macrophages in immune responses: M1‐like TAMs promote antitumor immunity by enhancing pathogen clearance and restricting tumor growth, whereas M2‐like TAMs support angiogenesis, tissue repair, and suppression of cytotoxic T cells, thereby facilitating tumor progression [20]. However, the limitations of this binary model have become increasingly apparent, particularly in complex diseases such as cancer, autoimmune disorders, and cardiovascular conditions. Evidence now indicates that heterogeneous macrophage populations coexist within tumors, collectively shaping tumor growth and immune responses [21, 22]. Accumulating evidence increasingly supports the need for a detailed understanding of TAMs heterogeneity and functionality in clinical and therapeutic settings [23].

In this review, we will examine the crucial role of TAMs in the TME, whether they suppress or promote tumor progression. We will further investigate the various underlying mechanisms of the bidirectional interactions between cancer cells and TAMs, including phagocytosis‐mediated signaling, metabolic reprogramming, exosome‐mediated communication, and the interplay between TAMs and other immune cells within the TME. These interactions not only shape the tumor landscape and define the immune environment but also drive disease progression and therapeutic failure. Understanding this cellular crosstalk will facilitate the development of strategies to effectively target cancer and its supportive immune network.

## 2 Macrophage Polarization States in the TME

TAMs constitute a distinct macrophage population residing within the TME. In response to diverse microenvironmental cues, these macrophages undergo phenotypic and functional diversification through a process known as TAMs polarization. Elucidating the cellular and molecular mechanisms governing TAMs polarization in the TME is crucial for advancing our comprehension of tumor pathogenesis and may reveal novel therapeutic avenues for oncology. This section will concentrate on the polarization dynamics of TAMs and their functional implications within the TME.

### 2.1 M1 Macrophages in the TME

M1‐like TAMs execute antitumor functions through a multistep mechanism initiated by recognition of malignant cells through altered surface antigen alterations or abnormal glycosylation signatures. These macrophages detect abnormal carbohydrate structures, including those presented on carcinoembryonic antigen and Tn antigen, through membrane‐bound lectin‐like receptors, thereby enabling discrimination between transformed and normal cellular phenotypes [24]. This recognition triggers a cascade of cytotoxic responses, including direct tumor cell killing through phagocytosis, the generation of reactive oxygen species (ROS)/reactive nitrogen species, and antibody‐dependent cell‐mediated cytotoxicity (ADCC) [25]. Upon activation, M1‐like TAMs further target tumor cells by producing nitric oxide (NO), which induces DNA damage and apoptosis [26]. They also secrete proinflammatory cytokines (e.g., IFN‐γ, IL‐12), which enhance the infiltration and activation of natural killer (NK) cells and cytotoxic T lymphocytes (CTLs) within the TME [27]. Furthermore, as innate immune effectors, M1‐like TAMs bridge to adaptive immunity through postphagocytic tumor antigen presentation [28]. Their ADCC activity represents an adaptive immune interface: tumor‐specific antibodies opsonize cancer cells, and macrophages bind the Fc region of these antibodies to engulf and eliminate the targeted cells [29]. Tumor cells can evade this mechanism by downregulating recognition molecules and activating inhibitory pathways [28, 30]. Beyond antitumor mechanisms of M1‐like TAMs include Dectin‐1‐mediated enhancement of NK cell cytotoxicity, increased tumor necrosis factor‐related apoptosis‐inducing ligand (TRAIL)‐induced cell death through NK cell recruitment in fibrotic tumor regions [31], and costimulatory support for effector T cell activation. Compared with protumor M2‐like TAMs, M1‐like TAMs exhibit reduced secretion of VEGF, MMPs, and CCL [18], thereby suppressing tumor angiogenesis and metastasis [27, 32].

### 2.2 M2 Macrophages in the TME

M2‐like TAMs within the TME promote malignancy through direct and indirect interactions with cancer cells. These interactions enhance tumor cell proliferation, stimulate angiogenesis, drive EMT, remodel the ECM to facilitate metastasis, and reprogram the TME into an immunosuppressive niche.

#### 2.2.1 Promotion of Tumor Growth and Survival

**FIGURE 2:**
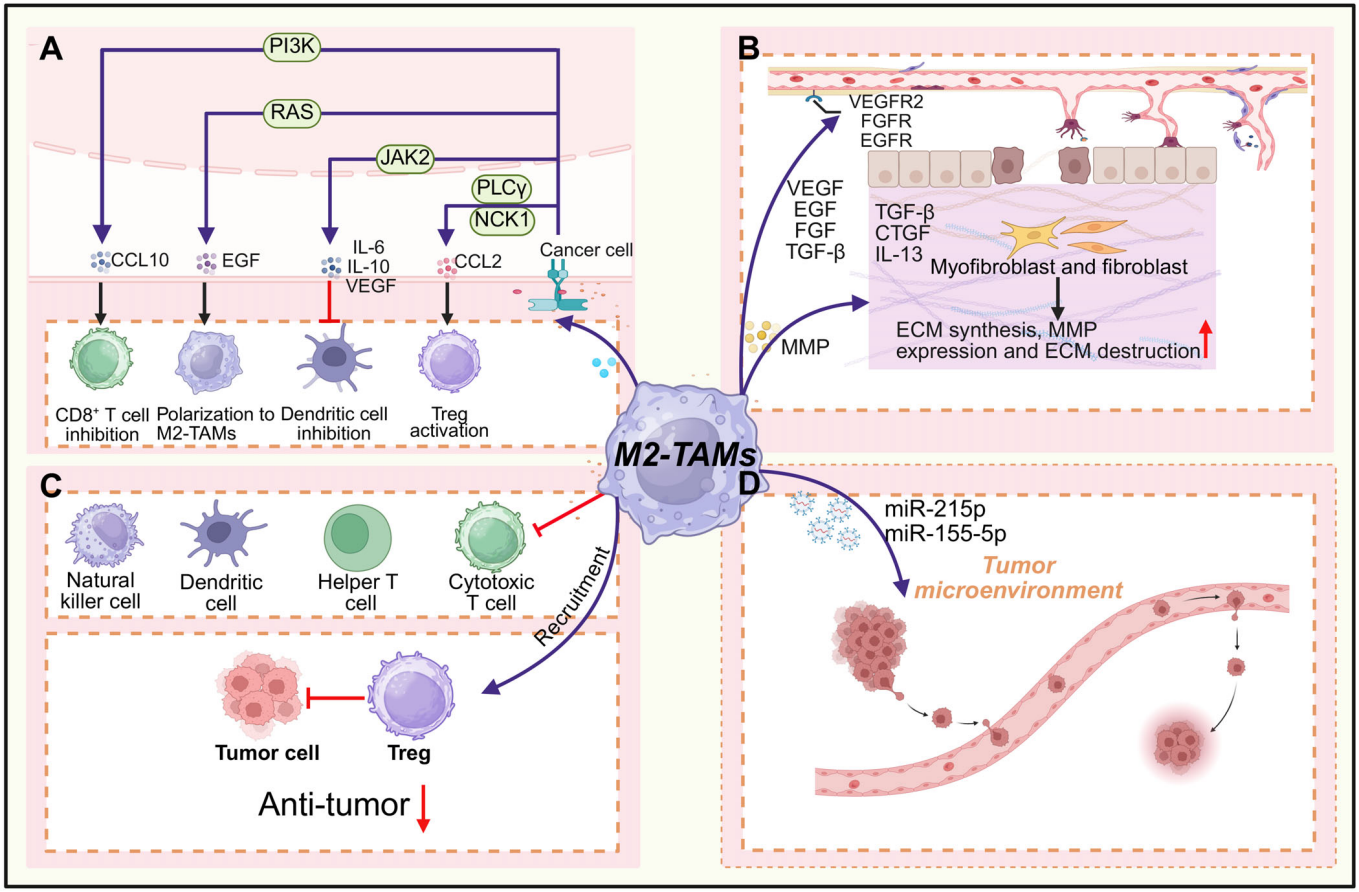
Protumor functions of M2–TAMs. M2–TAMs promote different aspects of tumor development. (A) M2–TAMs activate PI3K/RAS/JAK2 pathways to inhibit CD8^+^ T cells, polarize M2 macrophages, suppress DCs, and activate Tregs, establishing an immunosuppressive microenvironment. (B) M2–TAMs and tumor cells secrete angiogenic factors that activate cognate receptors on endothelial cells to induce neovascularization. Moreover, M2–TAMs and stromal cells (myofibroblasts, fibroblasts) secrete cytokines, which drive ECM synthesis, upregulate MMP expression, and mediate ECM degradation, thereby enabling tumor invasion and metastasis. (C) M2–TAMs suppress NK cells, DCs, helper T cells, and cytotoxic T cells, while recruiting Tregs to attenuate antitumor immunity. (D) TAMs‐derived exosomes can promote tumor metastasis and premetastatic niche formation.

Replicative immortality, a hallmark feature of malignancies, is promoted by increased TAMs infiltration across multiple cancers, such as breast, endometrial, and renal cell carcinoma [33]. In vivo coculture experiments involving macrophages and tumor cells underscore the significance of infiltrated TAMs in promoting tumor cell proliferation. Consequently, depletion of TAMs significantly impairs tumor growth [33]. Enhanced expression of growth factor receptors and their associated signaling networks, particularly the epidermal growth factor receptor (EGFR) and fibroblast growth factor (FGF) receptor families, plays a major role in establishing the malignant phenotype of human tumors by driving uncontrolled proliferation and promoting cancer cell survival [34]. TAMs utilize this mechanism by secreting growth factors such as epidermal growth factor (EGF) and FGF, which activate these pathways, thereby promoting cancer cell survival, migration, metastasis, and resistance to apoptosis [34]. In oncology, TGF‐β exhibits a context‐dependent duality: it can suppress tumorigenesis in early stages while promoting metastasis in advanced disease. In late‐stage cancers, diminished sensitivity to the growth‐inhibitory effects of TGF‐β leads to enhanced proliferation, survival, angiogenesis, and immune suppression [35]. Emerging evidence indicates that TAMs‐derived TGF‐β promotes colorectal cancer (CRC) progression through the HIF1–TRIB3 signaling axis [36]. Furthermore, M2‐like TAMs secrete substantial amounts of the immunosuppressive cytokine IL‐10, which inhibits the antitumor activity of CD8^+^ T cells, Th1 cells, and NK cells [37], thereby diminishing cytotoxic immune responses and facilitating tumor survival (Figure 2A). Furthermore, TAMs‐derived adrenomedullin engages endothelial cells and promotes tumor growth by activating the eNOS pathway in a paracrine manner [38]. Collectively, these findings demonstrate that tumor progression is intricately linked to the molecular signals and immunosuppressive factors released by M2‐like TAMs.

#### 2.2.2 Angiogenesis and ECM Remodeling

M2‐like TAMs play a pivotal role in stimulating tumor angiogenesis, ensuring that rapidly proliferating cancer cells receive the oxygen and nutrients necessary for sustained growth. This proangiogenic function has been consistently observed in various preclinical models of ovarian, cervical, prostate, and breast cancers, as well as melanoma. Although tumor cells themselves can initiate blood vessel formation through proangiogenic signals, ablation of TAMs substantially attenuates this process. Zeisberger et al. demonstrated that depleting TAMs using liposome‐encapsulated clodronate (clodrolip) markedly reduced the density of blood vessels within tumor tissue, underscoring the essential role of TAMs in tumor neoangiogenesis. TAMs secrete a broad spectrum of proangiogenic molecules that drive neovascularization, a critical process for tumor growth and metastasis. For instance, TAMs release growth factors like VEGF, platelet‐derived growth factor (PDGF), TGF‐β, and FGF, which collectively promote angiogenesis across multiple cancer types, including gliomas, esophageal, breast, bladder, and prostate cancers (Figure 2B). Castro et al. noted that in glioblastoma, VEGF blockade by bevacizumab downregulated macrophage migration inhibitory factor at the invasive margin, consequently expanding the M2‐like TAMs population [39]. This adaptive response ultimately promoted disease progression and acquired resistance to bevacizumab. Intriguingly, proangiogenic factor profiles are subtype‐specific: FGF signaling regulates M2a‐induced angiogenesis, while placental growth factor signaling modulates M2c‐driven angiogenesis. In addition to secreting soluble factors, TAMs‐derived MMPs, plasmin, and urokinase plasminogen activator degrade the ECM, thereby facilitating angiogenesis and potentially enabling metastatic dissemination.

Persistent hypoxia combined with disordered vascular remodeling constitutes a hallmark of malignant angiogenesis. Zhang et al. demonstrated that hypoxia drives M2‐like TAMs polarization through a pathway dependent on the transcription factor hypoxia‐inducible factor‐1α (HIF‐1α). In glioblastoma, hypoxic M2‐like TAMs enhance VEGF secretion by activating the PI3K/Akt/Nrf2 signaling, a process that further enhances tumor drug resistance, angiogenesis, and tumor progression [40]. Notably, M2‐like TAMs express the endothelial‐specific tyrosine‐protein kinase receptor Tie‐2 (also known as the angiopoietin‐1 receptor), which engages angiopoietins to regulate vessel maturation. Experimental evidence indicates that selective inhibition of TIE2 expression specifically in TAMs effectively suppresses neovascularization and impedes tumor progression in multiple tumor models. These TIE2‐expressing macrophages demonstrate chemotactic responses to angiopoietin‐2 (ANG2), a ligand produced by developing blood vessels and activated endothelial cells. Hypoxic conditions further upregulate both TIE2 and ANG2, establishing a feedforward loop that stimulates blood vessel formation through autocrine and paracrine mechanisms. Consequently, selective elimination of M2‐like TAMs, for instance through suicide gene strategies, represent a promising approach to disrupt pathological angiogenesis and inhibit tumor progression.

#### 2.2.3 Immune Suppression

The tumor immune microenvironment (TIME) serves as a critical regulator of oncogenesis and therapeutic outcomes. Within the TIME, M1‐like TAMs enhance antitumor immunity through producing proinflammatory cytokines such as IFN‐γ and TNF‐α, which activate T cells and other effector immune cells. Furthermore, M1‐like TAMs function as potent antigen‐presenting cells, supporting T cells in precisely recognizing and inducing apoptosis in cancer cells. Conversely, M2‐like TAMs establish an immunosuppressive niche through upregulation of inhibitory surface markers and secretion of anti‐inflammatory cytokines and chemokines. Extensive experimental evidence demonstrates that macrophages in the TME frequently polarize toward the protumor M2 phenotype [41, 42]. These M2‐like TAMs secrete diverse immunomodulatory molecules, including chemokines, cytokines, and enzymes, that suppress the activity of cytotoxic T cells, NK cells, and other immune effector cells (Figure 2C). This immunosuppressive reprogramming allows TAMs to hinder antitumor immune responses and contribute to therapy resistance in solid tumors. Additionally, M2‐like TAMs foster the recruitment and activation of Tregs, creating a positive‐feedback loop that sustains an immunosuppressive TME. In this loop, M2‐like TAMs stimulate the differentiation of CD4^+^CD25^+^ T cells into Tregs, which in turn promote the polarization of monocytes toward an M2 phenotype [43]. This reciprocal crosstalk attenuates T cell‐mediated tumor elimination and facilitates immune escape mechanisms, ultimately accelerating tumor progression and chemotherapeutic resistance. Moreover, the expression of immune checkpoints such as programmed death receptor‐1 (PD‐1) on TAMs further inhibits their phagocytic and cytotoxic capabilities toward tumor cells while also reinforcing M2‐like polarization. Collectively these mechanisms highlight the multifaceted role of M2‐like TAMs in establishing and maintaining an immunosuppressive microenvironment that enables tumor immune escape [44, 45].

#### 2.2.4 Metastasis and Premetastatic Niches Formation

Cancer metastasis, characterized by the dissemination of neoplastic cells from a primary tumor to distant organ sites, remains the leading cause of cancer‐related mortality worldwide [46, 47] and is consistently associated with poor clinical outcomes [48, 49]. Macrophages represent a dominant cellular component within the TME, frequently over 50% of the total tumor mass [50]. Accumulating evidence underscores their pivotal role in orchestrating metastasis, particularly by establishing premetastatic niches (PMNs) [51, 52, 53, 54, 55]. The formation of PMNs involves both TRMs and recruited monocyte‐derived macrophages, which initially maintain antigen‐presenting capacity and immunostimulatory potential [56]. Concurrently, primary tumors undergo pathological processes including dysregulated proliferation, chronic inflammation, and hypoxia [57]. These conditions induce the systemic release of tumor‐derived secretomes, including soluble factors, extracellular vesicles (EVs), and other bioactive molecules, which hematogenously prime future metastatic sites and recruit immunosuppressive leukocytes such as macrophages [58, 59] (Figure 2D). Once recruited, macrophages participate in establish a self‐sustaining inflammatory loop within developing PMNs. Tumor‐derived EVs and soluble mediators perpetuate macrophage infiltration and reprogram them toward an immunosuppressive, proangiogenic M2‐like state. For instance, caveolin‐1 carried in breast cancer‐derived EVs has been shown to promote PMNs formation by simultaneously inducing prometastatic gene signatures in pulmonary epithelial cells and promoting M2 polarization of alveolar macrophages [60]. Macrophages dynamically interface with soluble tumor‐derived signaling factors, undergoing comprehensive phenotypic and functional reprogramming that promotes ECM remodeling, chronic inflammation, immune suppression, and angiogenesis [61]. Moreover, TAMs orchestrate the infiltration of Tregs into PMNs through CCL22‐mediated chemotaxis, further reinforcing local immunosuppression [62]. Their inherent plasticity enables them to adapt to microenvironmental cues, amplifying oncogenic processes such as matrix remodeling and metastatic dissemination. In summary, M2‐like TAMs not only play a pivotal role in driving primary tumor progression but also actively facilitate PMNs formation, thereby creating a permissive niche for disseminated tumor cells from the primary site. These findings underscore the dual function of TAMs in both local tumor promotion and systemic metastatic dissemination.

## 3 Mechanisms Underlying TAMs–Tumor Interactions

The complex bidirectional signaling between tumor cells and TAMs and its impact on tumorigenesis have been thoroughly discussed in several authoritative reviews [63, 64, 65, 66]. A pivotal pathway through which TAMs modulate tumor development involves the regulation of cancer cell phagocytosis. This regulatory interaction is orchestrated by delicate balance between prophagocytic “eat‐me” signals and antiphagocytic “don't eat‐me” signals expressed on the surface of tumor cells. Prophagocytic ligands include calreticulin (CRT) [67], signaling lymphocytic activation molecule family (SLAMF)7 [68], Fc [69], and phosphatidylserine (PtdSer) [70], which promote macrophage‐mediated engulfment. Conversely, tumor cells frequently upregulate antiphagocytic ligands such as CD47, PD‐L1, major histocompatibility complex class I (MHC I), and cluster of differentiation 24 (CD24) to evade immune clearance [71]. These “don't eat‐me” ligands bind to specific inhibitory receptors on TAMs, thereby suppressing their phagocytic activity and facilitating tumor cell survival and proliferation. Clinical evidence demonstrates that TAMs infiltration, particularly with preferential polarization toward the immunosuppressive M2 phenotype, has been strongly correlated with poor prognosis and decreased overall survival in a wide range of malignancies, including melanoma, breast, pancreatic, ovarian, head and neck, bladder, and renal cell carcinomas [72] (Table 1). This highlights the critical importance of TAMs–tumor interactions in promoting tumor immune evasion and highlights the therapeutic potential of strategies that reprogram TAMs or disrupt these inhibitory pathways to restore their antitumor functions.

**TABLE 1:** Clinical trials targeting phagocytic signals.

Target	Drug	Clinical trials	Clinical phase	Tumor type
CD47	Hu5F9‐G4	NCT03527147 NCT05807126 NCT04788043 NCT02953782 NCT03558139 NCT04541017 NCT02953509 NCT03922477 NCT04751383	Phase 2 Phase 1 Phase 1 Phase 1/2 Phase 1 Phase 1/2 Phase 1/2 Phase 1 Phase 1	NHL Prostate cancer Hodgkin lymphoma Colorectal cancer Solid tumor Lymphoma Lymphoma AML Neuroblastoma
	AK117 HX009 SHR2150 Doxorubicin CC‐90002 HCB101 AK112	NCT06508606 NCT04980885 NCT04886271 NCT04588324 NCT05467670 NCT02367196 NCT05892718 NCT05229497	Phase 2 Phase 1/2 Phase 2 Phase 1/2 Phase 2 Phase 1 Phase 1 Phase 1/2	HNSCC AML Solid tumor Solid tumors Ovarian cancer Solid tumors Solid tumors Malignant tumors
Siglec‐10	ONC‐841	NCT06219499 NCT06352359	Phase 1 Phase 1	Solid tumor Solid tumor
CD24	IMM‐47	NCT05985083	Phase 1	Solid tumor
	CD24Fc	NCT04552704 NCT03960541	Phase 2 Phase 2	Solid tumor HIV
LILRB1/2	NGM707	NCT04913337	Phase 1	NSCLC
	PF‐07826390	NCT06546553	Phase 1	NSCLC
LILRB1	BND‐22	NCT04717375	Phase 1	Solid tumor
	AGEN1571	NCT05377528	Phase 1	Solid tumor
PD‐1/PD‐L1	Camrelizumab	NCT05222035	Phase 2	Solid tumor
	PD‐1 inhibitor + capecitabine	NCT05290194	Phase 2	Solid tumor
	PD‐1 inhibitor + GP	NCT05340270	Phase 2	Solid tumor
	PD‐1 inhibitor + tenofovir	NCT07133776	Phase 1/2	Solid tumor
	Sindilimab	NCT04799639	Phase 2	Cervical cancer
	PD‐1 inhibitor + lenvatinib	NCT06333561	Phase 1	NSCLC
	PD‐1 inhibitor + pemigatinib	NCT05913661	Phase 2	Intrahepatic cholangiocarcinoma
	Pembrolizumab	NCT07159217	Phase 2	Biliary tract cancer
	Serplulimab	NCT05675033	Phase 2	Solid tumor
	PD‐1 inhibitor + gemcitabine	NCT04624984	Phase 2	Refractory CHL

*Note*: Data resources come from https://clinicaltrials.gov, covering a time span of approximately 5 years.

### 3.1 The Phagocytic Signals Between TAMs and Tumor Cells

#### 3.1.1 “Eat‐Me” Signals

##### 3.1.1.1 CRT

**FIGURE 3:**
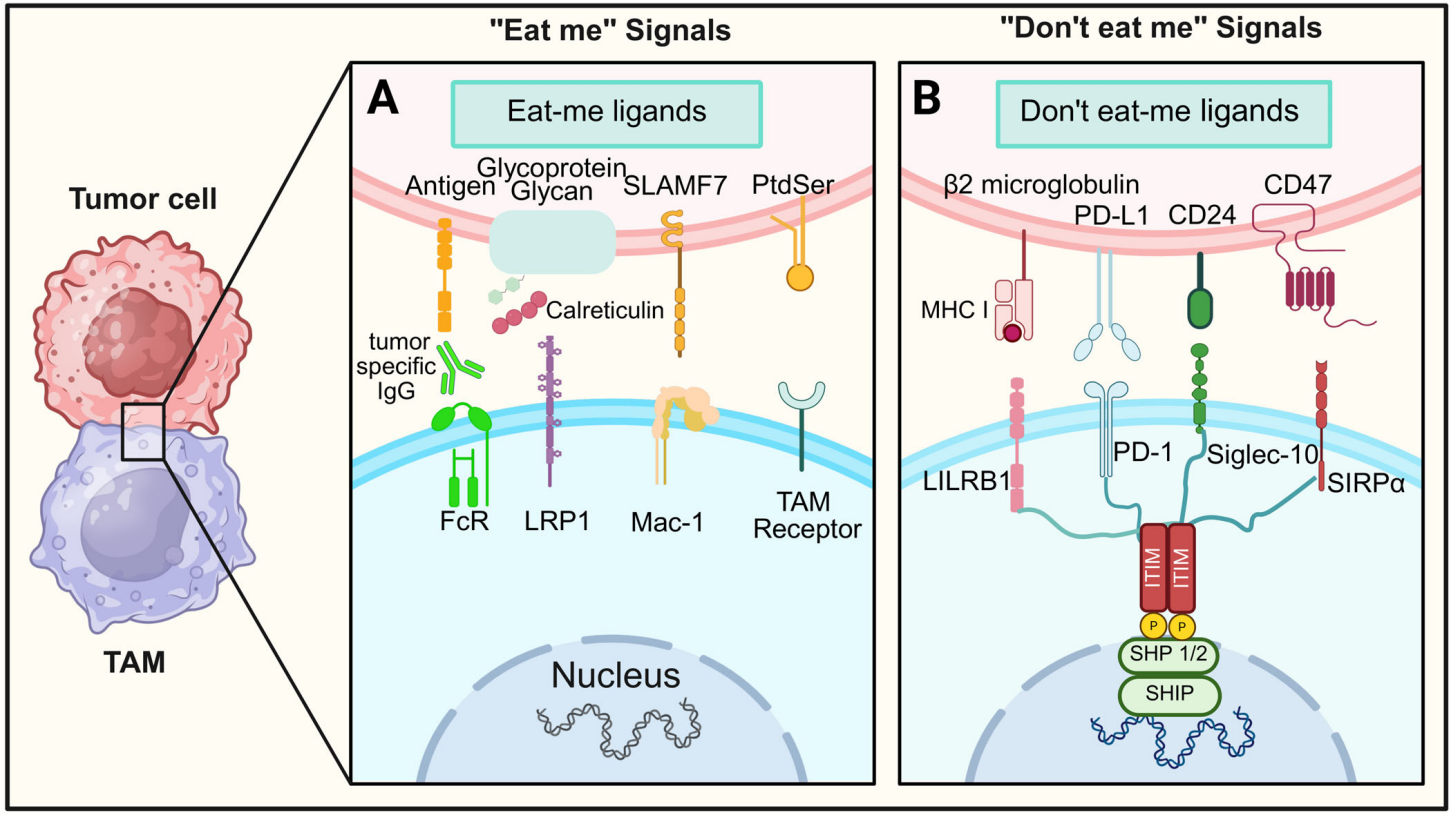
The signals of interactions between tumor cells and TAMs. The influence of macrophages on tumor cells is regulated by “eat me” and “don't eat me” signals, which are mediated through receptor–ligand interactions that occur after cell–cell contact. (A) Tumor cells present “eat me” ligands (antigens, glycoprotein–glycan, SLAMF7, PtdSer) that engage cognate receptors on TAMs (FcR complexed with tumor‐specific IgG, LRP‐1, TAM receptor, Mac‐1). These interactions initiate phagocytic signaling cascades in TAMs, promoting tumor cell efferocytosis. Dysregulation of these pathways facilitates tumor escape from phagocytic surveillance, highlighting their translational relevance for the development of prophagocytic immunotherapeutic modalities. (B) Tumor cells express “don't eat me” ligands (β2‐microglobulin/MHC I, PD‐L1, CD47, CD24) that interact with inhibitory receptors on TAMs (LILRB1, PD‐1, Siglec‐10, SIRPα). Engagement of these axes triggers intracellular inhibitory signaling (mediated by SHP1/2, SHIP) that abrogates TAMs phagocytic activity, enabling tumor immune evasion. Targeting these “don't eat me” checkpoints (e. g., anti‐CD47, anti‐CD24, anti‐PD‐L1 therapeutics) represents a pivotal strategy in cancer immunotherapy to restore TAMs‐mediated tumor clearance.

As integral components of the endoplasmic reticulum (ER) “lectin chaperone” family, CRT and calnexin play key roles in diverse cellular functions. Under physiological conditions, CRT primarily acts as a molecular chaperone and Ca^2^^+^ buffer, facilitating the proper folding of newly synthesized proteins within the ER. Beyond its role in protein homeostasis, CRT participates in Ca^2^^+^‐dependent processes including cell adhesion, integrin signaling, and antigen presentation through MHC I molecules, thereby contributing to immune surveillance [73]. CRT serves as a crucial “eat‐me” signal that triggers phagocytosis and downstream immune responses through multiple pathways, with the protein being derived from both target cells and macrophages (Figure 3A). In apoptotic cells, CRT exposed on the cell surface acts as a recognition ligand. It forms a bridging complex with C1q and the low‐density lipoprotein receptor‐related protein 1 (LRP‐1, also known as CD91) expressed on phagocytes, initiating phagocytic clearance [74, 75, 76]. During tumor cell death, CRT exposure promotes immunogenic cell death, facilitating the uptake of dying cancer cells by dendritic cells (DCs). This process enables DCs to process and present tumor‐associated antigens, subsequently activating adaptive immune responses. Intriguingly, CRT can also be expressed on the cell membranes, where it similarly mediates the macrophage‐dependent engulfment of dysfunctional or malignant cells [77, 78]. In such contexts, activated macrophages are often the primary source of CRT‐mediated recognition, targeting cells that have lost homeostatic integrity. For instance, during inflammation, neutrophils that infiltrate affected tissues mature and undergo clearance by macrophages, a process that is partly dependent on CRT exposure.

Collectively, these findings highlight CRT's dual role as both a homeostatic chaperone and a pivotal regulator of phagocytic recognition, thereby connecting innate and adaptive immunity through the elimination of pathogenic or transformed cellular elements.

##### 3.1.1.2 Signaling Lymphocytic Activation Molecule Family

The SLAMF receptors function as pivotal regulators in mediating phagocytic clearance of hematopoietic malignancies [79]. This receptor family encompasses nine members, seven of which (CD48, Ly9, CD244, CD84, SLAMF7, SLAMF8, and SLAMF9) are known to be expressed on macrophages [80, 81]. Among these receptors, SLAMF7 (also known as CS1 or CD319) has been identified as a crucial “eat‐me” signal that enhances phagocytic uptake of hematopoietic tumor cells through its interaction with the integrin Mac‐1 (Figure 3A). Structurally, SLAMF7 represents a transmembrane protein comprising three domains: an extracellular region, a transmembrane segment, and a cytoplasmic tail. The cytoplasmic tail contains immunoreceptor tyrosine‐based inhibitory motif (ITIMs) that include both activating and inhibitory tyrosine residues. While SLAMF7 demonstrates constitutive low‐level expression on CD4^+^ T cells, monocytes, macrophages, DCs, and B cells, its expression is significantly upregulated in both healthy and neoplastic plasma cell populations in myeloma. It is also detectable on NK cells and some CD8^+^ T cell subsets. Experimental evidence supports its functional importance: genetic deletion of SLAMF7 in mice impairs the phagocytic capacity of BMDMs against various B cell and myeloid‐derived cancer cell lines, particularly under conditions of CD47 blockade [82]. Notably, distinct from other SLAMF receptors, SLAMF7‐mediated phagocytosis does not require signaling lymphocyte activation molecule‐associated protein (SAP) adaptors. Instead, it engages Mac‐1 (a heterodimeric complement receptor comprising CD11b and CD18), which is widely expressed on innate immune cells, including macrophages and other phagocytes [83]. Through this bidirectional crosstalk, SLAMF7 forms a surface complex with Mac‐1 on macrophages, enabling the recruitment of immunoreceptor tyrosine‐based activation motif (ITAM)‐bearing adaptors, including FcRγ and DAP12. This initiates an activation cascade involving sarcoma family kinases (Src), spleen tyrosine kinase (Syk), and bruton's tyrosine kinase (Btk) that ultimately drives efficient phagocytosis of hematopoietic tumor cells. Notably, while Mac‐1 is indispensable for SLAMF7‐mediated phagocytosis, SLAMF7 does not participate in conventional Mac‐1/C3bi‐dependent phagocytic pathways [82], underscoring its unique role in tumor‐specific immune surveillance.

##### 3.1.1.3 Fc

Fc receptors (FcRs) are a family of cell surface receptors that bind to the Fc domain of immunoglobulins (Igs), thereby initiating downstream signaling pathways in multiple immune effector cells, including macrophages, DCs, and granulocytes [84, 85] (Figure 3A). Within the human FcγR family, FcγRIIB represents the exclusive inhibitory member, while the other members (FcγRI, FcγRIIA, FcγRIIC, FcγRIIIA, and FcγRIIIB) function as activating receptors [85, 86, 87]. FcγRs primarily bind Ig subclasses IgG1, IgG3, and IgG4; however, FcγRIIIB does not bind IgG4, and FcγRIIA is the principal receptor for IgG286. Upon IgG binding, activating FcγRs initiate phosphorylation of ITAM, which are present either in the ligand‐binding α‐chain (as in FcγRIIA and FcγRIIC) or in the associated signaling γ‐chain (as in FcγRI and FcγRIIIA) [84]. Phosphorylation of ITAM tyrosine residues by Src family kinases creates docking sites for Syk‐family tyrosine kinases, facilitating their recruitment and subsequent activation. These kinases further phosphorylate downstream targets, including Rac‐specific guanine nucleotide exchange factors (Rac‐GEFs) [88, 89]. Activated Rac‐GEFs stimulate GTPases including RhoA, Rac1, and Cdc42, which coordinate actin cytoskeleton remodeling through actin‐regulatory proteins, ultimately facilitating efficient phagocytosis [89, 90]. Unlike activating FcγRs, FcγRIIB, which is conserved in both human and murine models, possesses an ITIM within its α‐chain. Upon phosphorylation, the ITIM recruits phosphatases such as SHP1 and SHIP1 (Src homology‐2‐domain containing inositol polyphosphate 5′ phosphatase 1), transmitting inhibitory signals that counteract ITAM‐mediated activation [90]. Moreover, FcγRIIB facilitates the endocytic uptake of antibody–antigen complexes on target cells, thereby limiting the availability of antibodies for activating FcγRs and further dampening immune cell activation [91, 92, 93]. Collectively, the delicate equilibrium between activating and inhibitory FcγR signaling shapes the magnitude and outcome of antibody‐dependent effector functions, including antibody‐dependent cell‐mediated phagocytosis and ADCC.

##### 3.1.1.4 Phosphatidylserine

When macrophages establish direct membrane contact with apoptotic cells, they detect cell death through specific surface‐exposed markers. These molecular cues, known as “eat‐me” signals, facilitate the recognition and clearance of dying cells through phagocytosis. Among these, PtdSer is the most widespread, potent, and functionally versatile signal identified to date [94, 95, 96]. PtdSer is a glycerophospholipid component found at varying levels in multiple cellular membranes, including those of the ER, mitochondria, Golgi apparatus, and the plasma membrane of virtually every cell type (Figure 3A). Its unique role as an “eat‐me” signal stems from its highly asymmetric distribution within the membrane bilayer. Under physiological conditions, nearly all PtdSer is restricted to the inner, cytoplasmic leaflet of the plasma membrane, along with other amino phospholipids such as phosphatidylinositol and phosphatidylethanolamine. Under homeostatic conditions, this asymmetric distribution is actively maintained by flippases, which translocate PtdSer from the outer to the inner leaflet [97, 98]. However, during apoptosis or cellular stress, this asymmetry is disrupted by the concerted activation of scramblases and inhibition of flippases, leading to the externalization of PtdSer on the cell surface. Once exposed, PtdSer functions as a potent recognition ligand for macrophages and other phagocytes, marking the cell for engulfment and removal. This mechanism ensures the efficient engulfment of apoptotic cells and prevents the release of intracellular contents that could otherwise trigger inflammatory responses.

#### 3.1.2 “Don't Eat‐Me” Signals

##### 3.1.2.1 CD47/SIRPα Axis

The CD47/signal regulatory protein alpha (SIRPα) axis plays a central role in regulating immune responses and is a key target in cancer immunotherapy strategies. CD47, frequently overexpressed on malignant cells, functions as a dominant “don't eat‐me” signal that enables evasion of phagocytic clearance. Its receptor, SIRPα, is highly expressed on myeloid cells such as macrophages and DCs. Structurally, SIRPα comprises an extracellular Ig domain that binds CD47, and a cytosolic domain containing ITIMs. Ligand binding triggers phosphorylation of these ITIM motifs, facilitating recruitment of SH2‐containing protein tyrosine phosphatases (SHP) such as SHP‐1 and SHP2, initiating inhibitory signaling cascades that suppress phagocytosis [99, 100, 101]. When CD47 engages SIRPα, it effectively sends a “don't eat‐me” signal that prevents myeloid cells from phagocytosing and eliminating malignant cells (Figure 3B). This interaction represents a crucial immune evasion mechanism for tumors. Therapeutic blockade of the CD47/SIRPα pathway with anti‐CD47 monoclonal antibodies or SIRPα–Fc fusion proteins disrupts this inhibitory signaling, thereby reactivating the phagocytic capacity of macrophages and other innate immune cells to eliminate cancer cells more effectively [102, 103]. Magrolimab, a CD47‐targeted humanized IgG, is one such agent currently under clinical investigation. In a Phase 1b study, the combination of magrolimab and azacitidine demonstrated notable clinical efficacy in patients with higher‐risk myelodysplastic syndrome, especially those harboring TP53 mutations, a subgroup historically associated with poor prognosis and limited treatment options [104, 105, 106]. CD47‐targeted interventions demonstrate considerable promise in cancer immunotherapy, though several therapeutic challenges require resolution. A primary concern involves the ubiquitous expression of CD47 on healthy cellular populations, particularly erythrocytes, potentially inducing off‐target tumor effects [107, 108]. CD47 blockade frequently precipitates hematological complications including anemia through unintended opsonophagocytosis of circulating erythrocytes, which constitutively express CD47 as a protective “don't‐eat‐me” signal against splenic and hepatic macrophage‐mediated clearance [109]. The severity of these adverse events exhibits dose‐dependency and correlates with treatment duration and patient‐specific factors. IMM01, is a recombinant human SIRPα–Fc fusion protein engineered to selectively bind CD47 on tumor cells while exhibiting low‐affinity interactions with CD47 on erythrocytes. This unique design minimizes red blood cell binding, thereby reducing the risk of severe hemolytic anemia, a common dose‐limiting toxicity associated with conventional CD47/SIRPα inhibitors, without compromising its antitumor activity. Collectively, targeting the CD47/SIRPα immune checkpoint holds great promise for reprogramming macrophage phagocytosis and reversing cancer immune resistance.

##### 3.1.2.2 PD‐1/PD‐L1 Signals

PD‐1 is a 55‐kDa transmembrane glycoprotein that contains an extracellular Ig variable‐like ligand‐binding domain and cytoplasmic ITIMs that suppress immune signaling. Under physiological conditions, PD‐1 and its ligands (PD‐L1 and PD‐L2) help maintain peripheral tolerance by dampening excessive immune activation during self‐antigen recognition and tissue repair [110, 111] (Figure 3B). Tumor cells exploit this mechanism by upregulating PD‐L1 or PD‐L2 to engage PD‐1 on T cells and myeloid cells, effectively transmitting a “don't eat‐me” signal that enables immune evasion and tumor persistence [71, 112, 113, 114, 115]. Importantly, substantial evidence indicates that TAMs are closely involved in PD‐1/PD‐L1‐mediated immunosuppression. Within the TME, M2‐like TAMs express high levels of PD‐L1 and further undermine PD‐1/PD‐L1 blockade by secreting protumor cytokines and exosomal cargo, thereby sustaining an immunosuppressive niche [116, 117]. As tumors progress, the proportion of PD‐L1^+^ M2‐like TAMs increases, further inhibiting effective T cell recruitment and activation [118, 119]. This immunoregulatory loop contributes to inherent and acquired resistance to PD‐1/PD‐L1 immune checkpoint inhibitors (ICIs) in various cancers.

Clinically, monoclonal antibodies that block the PD‐1/PD‐L1 axis demonstrate remarkable therapeutic benefit across various tumor types, including Hodgkin lymphoma [120], non‐small cell lung cancer (NSCLC) [121, 122], and melanoma [123]. Pembrolizumab, a fully humanized IgG4κ monoclonal antibody developed by Merck, is one of the best‐characterized PD‐1‐blocking agents [124, 125, 126, 127, 128]. By binding to PD‐1, pembrolizumab disrupts the inhibitory signaling cascade, thereby reactivating exhausted T cells and restoring their antitumor cytotoxic functions [124, 129]. This blockade reinvigorates immune surveillance and enhances tumor clearance, underscoring the clinical relevance of targeting the PD‐1/PD‐L1 axis to overcome tumor immune escape. ICIs has revolutionized cancer treatment, demonstrating particular efficacy in patients with advanced‐stage disease [130]. Nevertheless, overall response rates remain suboptimal [131]. A subset of patients develops acquired resistance following initial response and disease relapse [132, 133], while others exhibit disease progression despite early clinical benefit [134]. A significant proportion of patients, however, demonstrate primary resistance and derive no benefit from treatment [132]. Furthermore, ICIs administration can provoke immune‐related adverse events (irAEs) affecting multiple organ systems. Although ICIs have achieved substantial success across diverse malignancies, their widespread clinical adoption is hampered by limited response rates and treatment‐emergent irAEs. It is therefore imperative to elucidate the determinants of therapeutic response, resistance mechanisms, and toxicity, underscoring the urgent need for predictive biomarkers of ICIs efficacy. The upregulation of PD‐1/PD‐L1/PD‐L2 expression in peripheral blood mononuclear cells (PBMCs) represents a potential novel mechanism of immune escape in lung cancer. Its detection may serve not only as a predictive biomarker for prognosis and treatment response, but PD‐L1 expression levels have now been established as a definitive clinical indicator for guiding ICIs therapy in NSCLC and urothelial carcinoma. Despite markedly improving outcomes in cancer patients, the therapeutic benefits of ICIs remain constrained by limited efficacy and considerable treatment‐related toxicities. To mitigate ICIs overtreatment and reduce irAE incidence, the development of robust predictive biomarkers for irAEs is critically needed [135]. Among emerging candidates, serum cytokine profiles may offer predictive utility for irAE risk stratification.

##### 3.1.2.3 MHC I/LILRB1 Signals

MHC I molecules fulfill dual immunological functions by mediating endogenous antigen presentation to CTLs while simultaneously regulating the phagocytic capacity of macrophages [136]. In vertebrated, the MHC represents a diverse set of highly polymorphic genes encoding the principal histocompatibility antigens. In humans, this gene complex is referred to as the human leukocyte antigen (HLA) system [137], which includes classical HLA‐I and HLA‐II molecules as well as nonclassical HLA‐III variants [138, 139]. Virtually all nucleated cells express MHC I molecules, enabling continuous immune surveillance by CD8^+^ cells. Emerging evidence highlights that MHC I also interacts with the leukocyte Ig‐like receptor B1 (LILRB1), an inhibitory receptor expressed on innate and adaptive immune cells. This molecular interaction establishes a critical regulatory checkpoint for phagocytosis and contributing to tumor immune evasion [140, 141, 142]. LILRB1, also known as CD85J or LIR‐1, classified within the LILRB subfamily and is broadly expressed on macrophages, NK cells, DCs, B cells, and subsets of T lymphocytes [143, 144, 145, 146, 147]. Structurally, LILRB1 features three key domains: an extracellular Ig‐like recognition domain that binds MHC I, a transmembrane segment, and an intracellular region that contains multiple ITIMs [148]. The binding of MHC I molecules to LILRB1, which occurs specifically through interaction with the MHC I α3 domain and its associated β2‑microglobulin subunit, leads to phosphorylation of the ITIM motifs on LILRB1, primarily mediated by Src family kinases [147, 149]. These phosphorylated ITIMs subsequently recruit SHIP, initiating downstream inhibitory signaling that attenuates macrophages activation [150] (Figure 3B). This SHIP‐dependent pathway concurrently attenuates ITAM‐mediated phagocytic signaling while potentiating the PI3K/AKT signaling axis [151, 152]. This process culminates in diminished macrophage phagocytosis, enhanced tumor cell survival, and impaired overall antitumor immune responses. Collectively, the MHC I/LILRB1 axis represents a critical “don't eat‐me” signal that tumor cells exploit to evade immune surveillance, underscoring its potential as a therapeutic target to enhance macrophages‐mediated phagocytosis in cancer immunotherapy.

##### 3.1.2.4 CD24/Siglec‐10 Signals

CD24, alternatively termed heat stable antigen (HSA) or small cell lung carcinoma cluster 4 Antigen, represents a highly glycosylated membrane protein covalently linked to the cell surface through a glycosylphosphatidylinositol anchor [153, 154]. The CD24 gene, localized to chromosome 6q21, encodes a polypeptide harboring up to 16 potential O‐ and N‐glycosylation motifs [155]. Comprehensive transcriptome profiling data from The Cancer Genome Atlas and Therapeutically Applicable Research to Generate Effective Treatments initiatives demonstrate upregulated CD24 expression profiles across multiple malignancies, underscoring its role in tumor immune evasion [156]. Functionally, CD24 engages with Siglec‐10 expressed on myeloid‐derived immune cells, including macrophages. This interaction serves as a critical “don't eat‐me” signal that attenuates potentially detrimental inflammatory responses in various pathological contexts, such as bacterial infection [157], sepsis [158], hepatic injury [159], and chronic graft‐versus‐host disease [160]. Upon binding CD24, the cytoplasmic tail of Siglec‐10, which contains two ITIMs, recruits the SHP‐1 and/or SHP‐2, initiating an inhibitory signaling axis (Figure 3B). This pathway suppresses Toll‐like receptor (TLR)‐mediated inflammatory signaling and impairs cytoskeletal rearrangements essential for macrophage‐mediated phagocytosis [161, 162, 163]. Functional studies employing gene knockout models and therapeutic monoclonal antibodies targeting the CD24/Siglec‐10 axis demonstrate significantly enhanced tumor cell phagocytosis by macrophages in vivo, resulting in suppressed growth of macrophage‐dependent neoplasms. Notably, the magnitude of this effect correlates with Siglec‐10 expression levels on macrophages, and ablation of Siglec‐10 eliminates the benefit of CD24 blockade, underscoring the specificity of this checkpoint pathway [164]. Collectively, these findings demonstrate that the CD24/Siglec‐10 axis suppresses macrophage phagocytosis, thereby facilitating tumor immune evasion. Although healthy tissues naturally exploit similar “don't eat‐me” mechanism to maintain self‐tolerance and prevent inappropriate clearance, tumor cells are often highly dependent on these antiphagocytic pathways to escape immune surveillance [165, 166, 167, 168]. Consequently, targeting the CD24/Siglec‐10 interaction represents a promising therapeutic strategy for unleashing macrophage‐mediated tumor clearance and enhancing the efficacy of cancer immunotherapies aimed at overcoming tumor immune escape [71].

### 3.2 Metabolic Crosstalk

**FIGURE 4:**
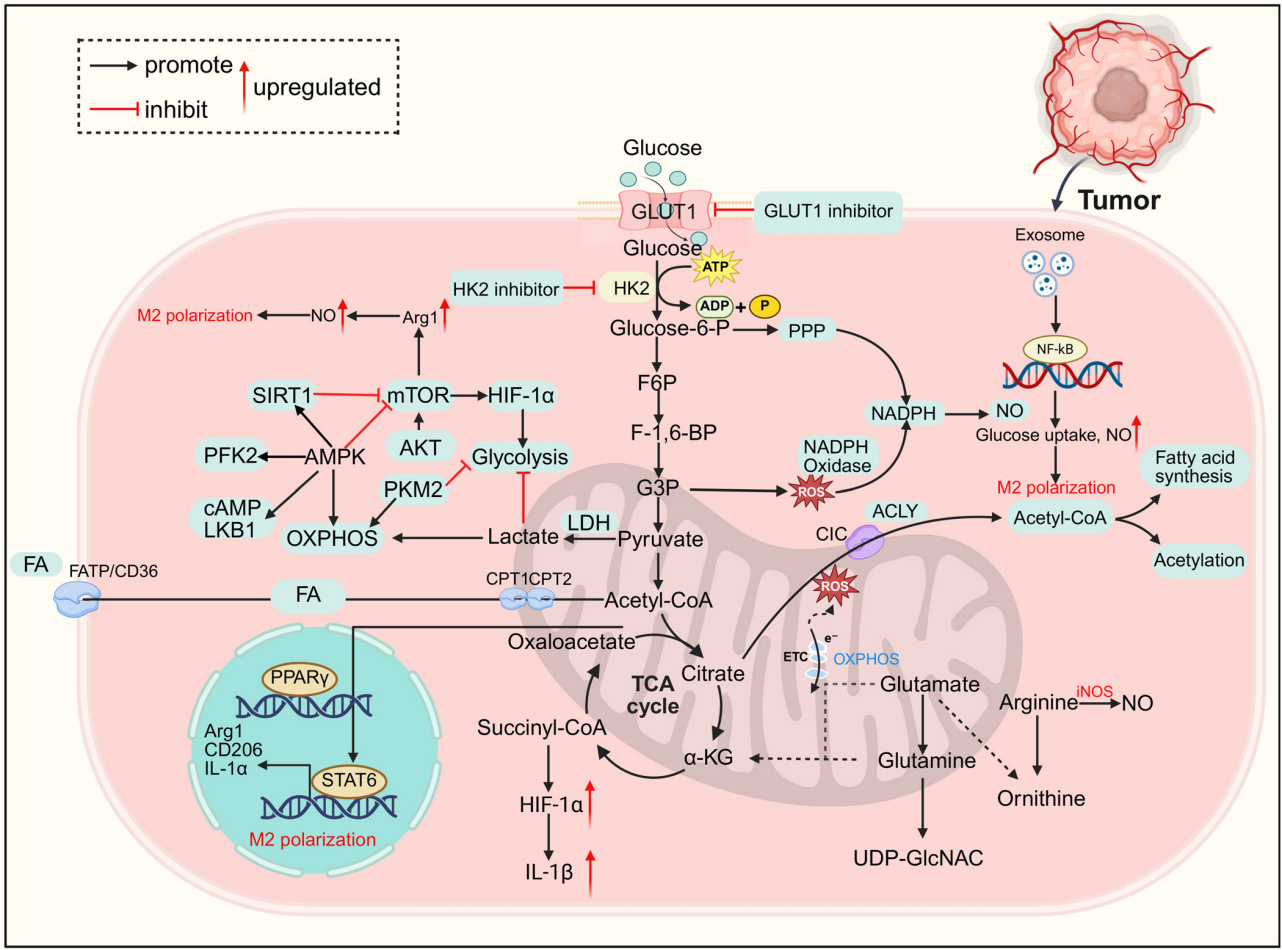
Metabolic reprogramming of TAMs polarization. Metabolic reprogramming of TAMs in the TME. TAMs exhibit specialized glycolytic reprogramming mediated by nitric oxide synthase 2 (NOS2): this enzyme generates NO, which in turn promotes TAMs activation and M2‐like polarization. Tumor‐derived exosomes amplify this immunosuppressive metabolic reprogramming through TLR2/NF‐κB signaling, enhancing glucose uptake and NOS2 expression to facilitate M2‐like polarization. A key step in cancer cell glycolysis is glucose uptake, which is mediated by GLUTs on the cell membrane. Inhibition of GLUT1 with specific small‐molecule inhibitors can selectively kill such cancer cells. HK2 is a pivotal enzyme that initiates glycolysis in cancer cells by catalyzing the phosphorylation of glucose to glucose‐6‐phosphate. The development of targeted HK2 inhibitors is still in its infancy. Enhanced fatty acid oxidation (FAO) drives mitochondrial oxidative phosphorylation (OXPHOS) and ROS production, which in turn activates the JAK1–STAT6 signaling axis, promoting M2‐like polarization and enhancing cancer cell invasiveness. Carnitine palmitoyltransferase (CPT) is a key rate‐limiting enzyme for FAO; inhibitors targeting CPT‐1A can suppress FAO‐dependent ROS and NOD‐like receptor pyrin domain‐containing protein 3 (NLRP3) production in M2‐like TAMs, thereby inhibiting IL‐1β secretion and limiting TAM‐mediated cancer cell migration. Arginase 1 (Arg1) mediates the conversion of arginine to ornithine and urea; its generated metabolites not only support tumor growth but also limit the availability of arginine required for nitric oxide (NO) synthesis. Polyamines and inhibitory cytokines derived from Arg1 further reinforce this immunosuppressive state. The PI3K/Akt/mTOR signaling axis maintains this immunosuppressive circuit by transcriptionally upregulating Arg1 expression. PI3Kγ inhibitors can disrupt this pathway, suppressing Arg1 while upregulating nitric oxide synthase (NOS) activity, thus shifting arginine metabolism toward NO production and enhancing tumor cytotoxicity.

Metabolic reprogramming has emerged as a pivotal regulator of cellular differentiation pathways and phenotypic determination [169]. Analogous to the Warburg effect, a hallmark of malignant cells, immune cells, including macrophages, undergo activation‐induced metabolic reprogramming, a phenomenon known as “immunometabolism,” to fulfill increased demands for biosynthesis and bioenergetics. Notably, these metabolic adaptations extend beyond ATP generation to actively orchestrate immune effector functions through transcriptional regulation and posttranslational modifications. Consequently, metabolic pathways are now acknowledged not merely as passive facilitators but as dynamic regulators of immune responses across physiological and pathological states [170, 171] (Figure 4).

#### 3.2.1 Glucose Metabolism

TAMs exhibit specialized glycolytic reprogramming mediated by NO synthase 2 (NOS2). This enzyme generates NO, thereby promoting TAMs activation and M2‐like polarization [172]. Tumor‐derived exosomes amplify this immunosuppressive metabolic reprogramming through TLR2/NF‐κB signaling, enhancing glucose uptake and NOS2 expression to establish a feedforward loop [51]. Glycolytic byproducts such as lactate further potentiate immunosuppression by activating the G‐protein coupled receptor 81 (GPR81/HCA1), thereby enhancing M2 polarization and anti‐inflammatory functions [173]. Consequently, lactic acid serves as a key regulator of TAMs‐mediated immunosuppression. Inhibiting glycolysis has become a critical therapeutic strategy. Currently, glycolytic inhibition has emerged as a strategically significant therapeutic approach. While broad‐spectrum inhibitors like 2‐deoxyglucose (2‐DG) lack specificity, the antidiabetic agent metformin offers a more refined strategy [174]. Metformin suppresses mitochondrial complex I activity, reducing ATP production and inducing energetic stress. This metabolic challenge activates AMPK, which enhances glucose uptake and glycolytic flux in TAMs, ultimately shifting their polarization toward a proinflammatory, antitumor phenotype [175]. Notably, as glycolysis concurrently supports macrophage ROS production and phagocytosis, its modulation must balance immunostimulatory and immunosuppressive effects.

#### 3.2.2 Fatty Acid and Lipid Metabolism

Emerging evidence underscores the pivotal role of lipid metabolism in shaping TAMs polarization. Notably, macrophage lipolysis is linked to immunosuppressive functions, whereas lipid synthesis pathways support proinflammatory activity [176]. Within the TME, enhanced fatty acid oxidation (FAO) drives mitochondrial oxidative phosphorylation (OXPHOS) and ROS generation, subsequently activating the JAK1–STAT6 signaling axis that promotes M2‐like polarization and enhances cancer cell invasion [177]. The TME provides diverse lipid substrates that TAMs internalize through receptor‐dependent pathways (CD36, scavenger receptor A), scavenger receptor class B type I) or through receptor‐independent mechanisms [178]. Once internalized, these lipids serve dual metabolic purposes: they can be catabolized to sustain cellular effector functions or stored as intracellular lipid droplets to confer metabolic flexibility [179]. Importantly, overexpression of lipid metabolic genes in TAMs correlates strongly with immunosuppressive polarization and poor clinical outcomes [180]. Consequently, therapeutic targeting of lipid metabolic pathways represents a promising clinical strategy to reprogram TAMs and inhibit tumor progression.

#### 3.2.3 Amino Acid Metabolism

The TME critically shapes the functional polarization of TAMs through amino acid metabolic reprogramming. Arginine metabolism serves as a pivotal switch directing macrophages toward either protumor or antitumor phenotypes [181, 182]. Arginase‐1 (Arg1) mediates the transformation of arginine into ornithine and urea, generating metabolites that support tumor growth while simultaneously limiting arginine availability for NO synthesis, a crucial mediator of antitumor immunity [183]. In both murine and human TAMs, Arg1‐derived polyamines and inhibitory cytokines reinforce this immunosuppressive state [184, 185]. This immunosuppressive circuit is maintained by the PI3K/Akt/mTOR axis, which transcriptionally upregulates Arg1 expression [186]. Notably, PI3Kγ inhibitors can disrupt this pathway, suppressing Arg1 while upregulating NOS activity, thereby redirecting arginine metabolism toward NO production and enhancing tumor cytotoxicity [187, 188]. Consequently, targeting PI3Kγ represents a viable strategy to reprogram TAMs metabolism and restore antitumor immunity. Furthermore, activation of the CD40–NF‐κB pathway in TAMs enhances proinflammatory cytokine secretion and upregulates costimulatory molecules [189]. Emerging research demonstrates that agonistic anti‐CD40 antibodies exert dual immunometabolic effects by coordinating glutamine metabolism and FAO through CD40 signaling, which drives epigenetic remodeling and polarization toward an antitumor TAMs phenotype [190].

In summary, immunometabolism dynamically reprograms TAMs polarization and effector functions through integrated crosstalk among glucose, lipid, and amino acid metabolic pathways. Deciphering these interconnected networks offers a strong rationale for targeting metabolic reprogramming as an innovative strategy to redirect TAMs polarization and counteract tumor‐mediated immune suppression.

### 3.3 Exosome‐Mediated Communication

**FIGURE 5:**
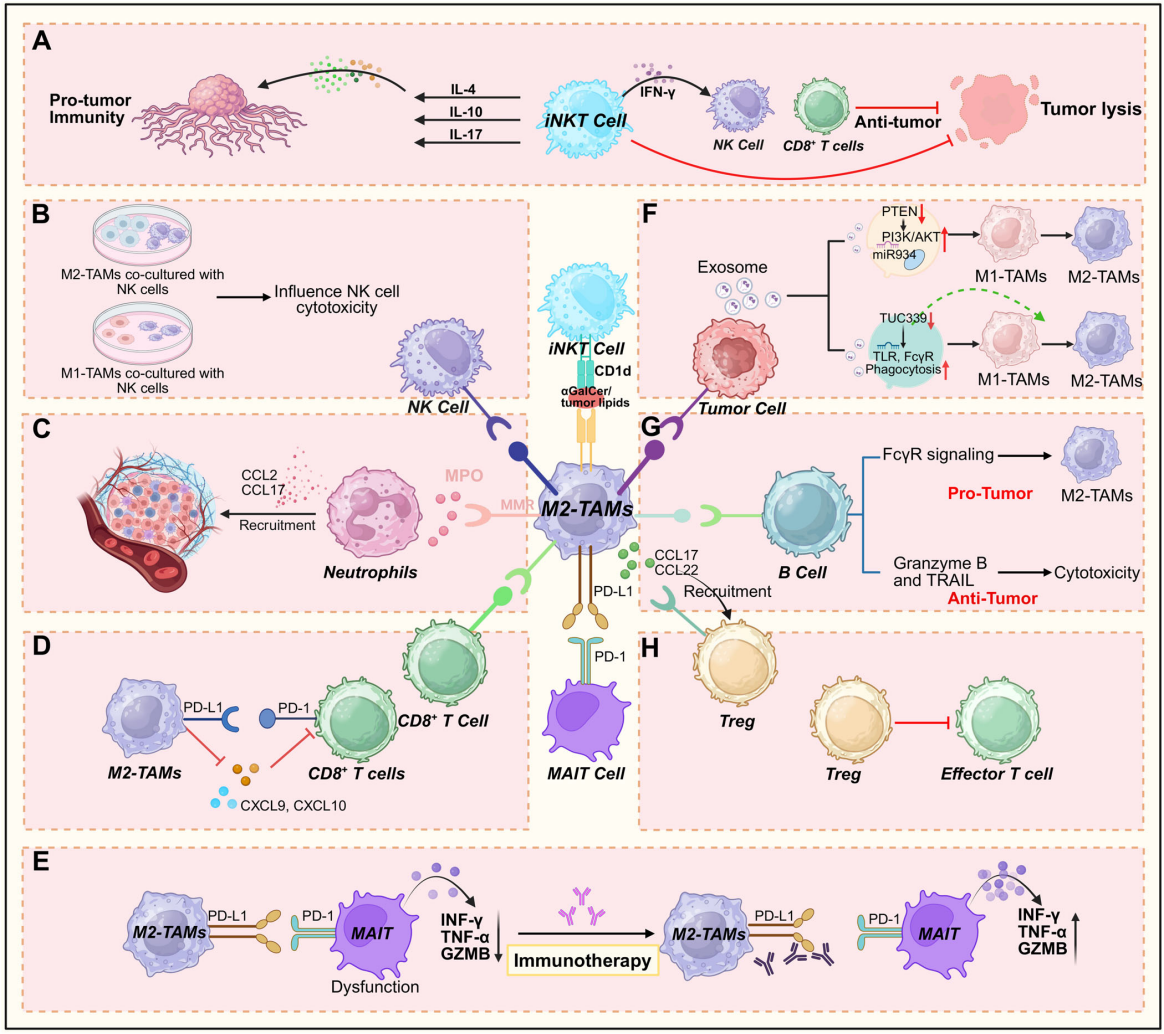
Interactions between M2–TAMs, TDEs, and other immune cells. M2–TAMs interactions with immune cells, delineating protumor and antitumor immune landscapes and identifying therapeutic targets for cancer immunotherapy. (A) iNKT cells exhibit dual, opposing roles in tumor immunity: they can drive tumor progression by secreting protumor cytokines (IL‐4, IL‐10, IL‐17), they also exert antitumor effects by producing IFN‐γ, this cytokine recruits NK and CD8+ T cells to the TME, ultimately promoting tumor cell lysis. (B) Coculture of M2–TAMs with NK cells impairs NK cell cytotoxicity, whereas coculture with M1–TAMs does not exert this inhibitory effect. (C) TAMs recruit neutrophils through chemokine signaling to facilitate protumor processes. M2‐like TAMs secrete CCL2 and CCL17, recruiting neutrophils that release MPO. TAMs express MMR, collectively contributing to protumor cascades. (D) M2–TAMs express PD‐L1, which binds to PD‐1 on CD8+ T cells; meanwhile, they reduce the secretion of chemokines (CXCL9/CXCL10), thereby inhibiting CD8+ T cell activation. (E) M2‐like TAMs induce MAIT cell dysfunction, with restoration by PD‐L1/PD‐1 blockade immunotherapy. M2‐like TAMs engage PD‐L1 with PD‐1 on MAIT cells, leading to diminished secretion of IFN‐γ, TNF‐α, and granzyme B, resulting in MAIT cell dysfunction. Immunotherapeutic blockade of PD‐L1/PD‐1 restores MAIT cell effector function, as evidenced by increased production of these molecules. (F) Tumor‐derived exosomes can drive M2‐like polarization by activating the PI3K/AKT signaling pathway; additionally, they can induce M2‐like polarization through TLR4 and FcγR‐mediated phagocytosis. (G) B cells can promote M2‐like TAMs polarization through FcγR signaling, and also secrete granzyme B/TRAIL to exert direct cytotoxicity. (H) M2‐like TAMs recruit Tregs by secreting CCL17 and CCL22, which in turn inhibit the function of effector T cells.

The TME represents a sophisticated biological network consisting of malignant cells and diverse stromal components, including cancer‐associated fibroblasts (CAFs), tumor‐infiltrating immune populations (notably macrophages), mesenchymal stromal cells, vascular endothelial cells, and an intricate vascular architecture, along with various soluble molecular factors [191]. Cellular communication within the TME orchestrates key aspects of malignant progression, including tumor growth, metastasis, and treatment resistance. Among these components, exosomes have emerged as critical mediators of this dynamic intercellular communication, attracting considerable scientific interest [192]. These nanovesicles transport biologically active cargo, including proteins, metabolites, and nucleic acids (DNA, mRNA, lncRNA, miRNA), enabling bidirectional communication that reprogram macrophages toward either to an M1‐like or M2‐like phenotype, depending on their molecular constituents [193]. Notably, ncRNAs within exosomes play pivotal roles in regulating protein expression, cellular processes, and immunomodulation [194]. Key ncRNA subtypes such as miRNAs, lncRNAs, and circRNAs modulate cellular proliferation, differentiation, and metabolic reprogramming through diverse molecular mechanisms (Figure 5F).

#### 3.3.1 Exosomal miRNAs

MiRNAs exert posttranscriptional control of gene expression by binding to complementary sequences on target mRNAs, ultimately inducing either translational blockade or transcript instability [195]. Emerging evidence has established exosomal miRNAs as pivotal modulators of TAMs function, influencing macrophage polarization, cytokine production, T cell‐mediated immunosuppression, and adaptation to hypoxic TME. For instance, in CRC liver metastases, Zhao and colleagues reported significantly upregulated miR‐934 levels within regions enriched with CD163^+^ TAMs. Tumor‐derived exosomal miR‐934 promoted M2 polarization by suppressing phosphatase and tensin homolog deleted on chromosome ten expression and activating the PI3K/AKT signaling pathway [196]. Similarly, CRC‐derived exosomes transporting miR‐145 have been shown to induce M2‐like polarization in macrophages, thereby facilitating an immunosuppressive microenvironment [197]. Furthermore, exosomal miRNAs can modulate metabolic enzymes: melanoma‐derived exosomal miR‐125b‐5p targets lysosomal acid lipase A in macrophages, promoting phenotypic transition and enhancing M2 macrophage survival [198].

#### 3.3.2 Exosomal lncRNAs

LncRNAs contribute significantly to multiple fundamental biological processes, including genomic imprinting, cell cycle regulation, differentiation, tumorigenesis, and metastasis [199, 200, 201]. Kogure et al. revealed significantly increased expression of lncRNA TUC339 in hepatocellular carcinoma (HCC)‐derived exosomes. This lncRNA facilitates intercellular communication between HCC cells, thereby promoting tumor proliferation and metastasis [202]. Importantly, exosomal TUC339 can be internalized by adjacent macrophages, promoting their polarization toward the immunosuppressive M2 phenotype and attenuating antitumor immunity. Transcriptome profiling revealed that exosomal TUC339 suppresses critical immune pathways in macrophages, particularly TLR signaling and FcγR‐mediated phagocytosis. Genetic knockdown of TUC339 was found to enhance macrophage phagocytic capacity, underscoring its functional relevance [203]. Complementary evidence from breast cancer studies demonstrate that tumor‐derived exosomal BCRT1 is readily taken up by macrophages, inducing M2 polarization and thereby augmenting malignant cell migration and chemotactic capacity, exacerbating tumor progression [204].

#### 3.3.3 Exosomal circRNAs

CircRNAs represents a unique subclass of ncRNA characterized by their covalently closed‐loop structure, conferring resistance to exonuclease‐mediated degradation and enabling stable intercellular transport through exosomes [205]. CircRNAs exert regulatory functions primarily by acting as competing endogenous RNAs, sponging miRNAs and thereby modulating downstream gene expression [206]. Lee et al. demonstrated that circ007293 is highly enriched in exosomes isolated from the serum of patients with papillary thyroid carcinoma (PTC) as well as in PTC cell culture supernatants [207]. Functionally, exosomal circ007293 was shown to promote EMT, cellular invasion, and proliferation by modulating the miR‐653‐5p/PAX6 axis. These findings highlight the potential of exosomal circ007293 as both a diagnostic biomarker for monitoring PTC progression and a promising therapeutic target. Collectively, exosome‐mediated transfer of ncRNAs represents a potent mechanism through which tumors reprogram TAMs and remodel the TME. This process facilitates immune evasion, tumor progression, and metastasis. A deeper understanding of the molecular intricacies underlying this vesicular communication network provides critical insights for developing novel therapeutic strategies aimed at intercepting tumor‐promoting exosomes–TAMs crosstalk.

### 3.4 Interactions With Other Immune Cells

TAMs serve as pivotal regulators of the innate immune system within the TME, with their origins and functions being closely linked to another crucial myeloid population—myeloid‐derived suppressor cells (MDSCs). MDSCs constitute a heterogeneous group of innate immune cells derived from myeloid precursors at various developmental stages. Under pathological conditions, they are extensively recruited into tumor tissues where they support tumor progression through multiple mechanisms, including enhancing tumor cell survival, stimulating angiogenesis, and promoting tissue invasion and metastasis. Notably, tumor‐infiltrating MDSCs can rapidly differentiate into TAMs, a process that substantially expands the macrophage population within the TME. TAMs serve as pivotal regulators within the innate immune compartment of the TME. Through bidirectional interactions with both adaptive and innate immune effectors such as B lymphocytes, T cells, NK cells, and neutrophils, TAMs modulate the equilibrium between protumor immunity and antitumor surveillance. These interactions profoundly shape the immunosuppressive networks that underpin tumor progression. Rational cotargeting of TAMs alongside complementary TME components holds significant promise for overcoming therapeutic resistance and achieving synergistic antitumor effects. However, an exclusive focus on TAMs and their interactions with conventional tumor cells and immune constituents provides an incomplete pathophysiological perspective. The complementary dimension involves the specialized bidirectional crosstalk between cancer stem cells (CSCs) and TAMs. This interaction assumes critical importance given CSCs’ defining competencies in tumor initiation, metastatic dissemination, and disease recurrence, coupled with TAMs’ dual role as both a predominant TME cellular population and active promoters of tumorigenesis (including those differentiated from MDSCs). The dynamically reinforced circuitry established between these cellular populations represents a fundamental biological mechanism underpinning tumor progression and maintenance.

#### 3.4.1 NK Cells

NK cells are critical effectors of the innate immunity, functioning as primary responders to sites of infection and tumorigenesis [208]. Unlike cytotoxic T cells, NK cells can eliminate malignant cells independent of MHC I expression, a feature that enables them to target tumor cells that evade adaptive immune recognition [209, 210]. However, the TME actively subverts NK cells antitumor activity by suppressing cytotoxic functions and downregulating tumor‐recognition receptors [211, 212]. Macrophages, particularly M2‐like TAMs, further modulate NK cell functionality by altering the expression profiles of activating and inhibitory receptors [211, 213, 214, 215, 216]. For instance, M2‐like TAMs suppress NK cell cytotoxicity, while M1‐like TAMs contribute to its preservation [217] (Figure 5B). In murine models, coculture of splenic NK cells with M2‐like TAMs reduces the expression of CD27 activation markers, whereas M1‐like TAMs maintain NK cell activation under similar conditions [211].

#### 3.4.2 Neutrophils

Neutrophils serve as pivotal regulators of inflammatory responses and exhibit functional plasticity analogous to macrophages, adopting either antitumor (N1) or protumor (N2) phenotypes depending on the microenvironmental cues [218, 219]. Upon activation, neutrophils orchestrate macrophage recruitment to inflammatory sites through secretion of chemotactic cytokines including IL‐8 and TNF‐α [220]. This interaction involves macrophage mannose receptor (MMR)‐dependent recognition and neutrophil‐derived myeloperoxidase (MPO) signaling [220] (Figure 5C). In HCC models, neutrophil‐derived chemokines such as CCL2 and CCL17 promote macrophage infiltration into tumors [221]. Notably, depletion of neutrophils exacerbates tumor progression, as demonstrated in 3‐methylcholanthrene‐induced sarcoma models using granulocyte colony‐stimulating factor receptor‐deficient mice. Neutropenic conditions are associated with increased M2‐like TAMs gene signatures and poorer clinical outcomes [218].

#### 3.4.3 B Lymphocytes

B cells exhibit context‐dependent immunomodulatory activities in cancer, exerting both protumor and antitumor effects that are shaped by local microenvironmental cues [222, 223, 224, 225, 226]. Their protumor functions can involve recruiting TAMs through FcγR‐dependent signaling triggered by immune complexes. In contrast, antitumor B‐cell responses include antigen presentation to T cells and direct cytotoxic activity mediated by granzyme B and TRAIL [224, 226] (Figure 5G). Studying B cells in carcinogenesis is challenging due to their phenotypic diversity within the TME and technical difficulties in isolating well‐defined B‐cell subsets [222, 225]. Notably, B cells and macrophages originate from a common developmental pathway: shared BM‑derived progenitor cells maintain the ability to commit to either lineage. Early pre‑B cells, which co‑express myeloid markers prior to B‑cell receptor rearrangement, retain the potential to differentiate into macrophages, highlighting the inherent plasticity of this hematopoietic lineage [227].

#### 3.4.4 T Cell

##### 3.4.4.1 Conventional T Cells

*CD4^+^ T Cells*: CD4^+^ T cells comprise multiple functionally specialized subsets, including Th1, Th2, Tregs, and follicular helper T cells [228]. Th1‐derived cytokines, such as IFN‐γ, drive classical M1‐like TAMs polarization, whereas Th2‐derived IL‐4 and IL‐13 promote alternative M2‐like TAMs activation [229, 230]. Consequently, the Th1/M1 axis is associated with robust antitumor immunity, whereas the Th2/M2 axis supports tumor progression [230]. Tregs, as a specialized CD4^+^ subset, orchestrate immunosuppressive networks that suppress cytotoxic immune responses against malignancies [231] (Figure 5H). Clinical studies have demonstrated coordinated infiltration of TAMs and Tregs in multiple malignancies, correlating with poor prognosis in colorectal [232], lung [233], and ovarian cancers [234]. Mechanistically, TAMs promote Tregs recruitment through paracrine signaling, releasing chemokines such as CCL17 and CCL22, which engage CCR4 on Tregs to facilitate their tumor infiltration [235, 236, 237, 238, 239].

*CD8^+^ T Cells*: CD8^+^ T lymphocytes are central to adaptive antitumor immunity, directly recognizing and eliminating transformed cells [240, 241]. A higher intratumoral density of CD8^+^ T cells is strongly correlated with improved clinical outcomes in many cancer types [242] (Figure 5D). However, M2‐like TAMs can suppress CD8^+^ T cell cytotoxicity, while M1‐like TAMs are associated with enhanced CD8^+^ T cell infiltration and activity [243]. Preclinical models, including Lewis lung carcinoma mice, demonstrate that T cells can reprogram TAMs toward an M1 phenotype within tumors [244]. Notably, TAMs and DCs are primary sources of PD‐L1 [245, 246], which interacts with PD‐1 receptors expressed on CD8^+^ T cells [242, 247]. This PD‐L1/PD‐1 axis acts as a key immune checkpoint, dampening T cell cytotoxicity and contributing to resistance against immune checkpoint blockade therapies [248].

##### 3.4.4.2 Unconventional T Cells

*NKT Cells*: NKT cells represent a specialized T cell population characterized by dual expression of αβ‐T cell receptors (TCR) for antigen recognition and typical NK cell markers including CD56, CD16, and granzyme [249]. Accounting for less than 1% of circulating T cells [250, 251], they share common lymphoid precursors with conventional T cells and undergo thymic maturation. Functionally and phenotypically distinct from conventional T cells, NKT cells recognize lipid antigens presented by CD1d molecules, whereas typical T cells identify peptide antigens presented by MHC I/II molecules [252] (Figure 5A). Based on TCR diversity, NKT cells divide into two subsets: Type I (invariant) NKT cells feature a conserved TCRα chain with limited TCRβ repertoire, while Type II (variant) NKT cells demonstrate diverse TCR chain combinations [253].

Emerging research on NKT cell biology has expanded their potential therapeutic applications across various diseases [253]. Type I NKT cells particularly demonstrate capacity to enhance antitumor immunity through TME modulation. In primary human neuroblastoma, these cells exhibit antitumor effects by eliminating TAMs [251]. The functional impairment of Type I NKT cells correlates with reduced iNKT cell frequencies in multiple malignancies including head/neck and prostate cancers [254]. Consequently, iNKT‐based combination immunotherapy represents a promising strategy for patients refractory to conventional immunotherapies [255]. Preliminary preclinical evidence strongly supports the potent antitumor capabilities of NKT cells.

*Mucosal‐Associated Invariant T Cells*: Mucosal‐associated invariant T (MAIT) cells represent a specialized subset of innate‐like T lymphocytes characterized by a semi‐invariant T‐cell receptor α‐chain (Vα19‐Jα33 in mice; Vα7.2‐Jα33/Jα12/Jα20 in humans) coupled with limited TCRβ‐chain diversity [256]. This distinctive TCR configuration confers specificity for antigens presented by the monomorphic MHC I‐related molecule MR1, which exhibits remarkable evolutionary conservation among mammalian species [257, 258]. MAIT cells exhibit pronounced tropism for mucosal tissues, including the lungs, liver, and intestines, and have been consistently identified within tumor‐infiltrating lymphocyte [259, 260].

Activation of MAIT cells triggers upregulation of the cytolytic mediators granzyme B and perforin [261, 262], and confers cytotoxic capacity against tumor cells presenting the microbial metabolite 5‐OP‐RU [263], indicating their potential for direct antitumor immunity. Beyond MR1‐restricted recognition, MAIT cells exhibit responsiveness to inflammatory cytokines [264, 265, 266] and express activating receptors such as DNAX accessory molecule‐1 that engage cognate ligands on malignant cells [266]. These findings collectively suggest that MAIT cell activation within the TME may occur through both MR1‐dependent and‐independent pathways. Notably, activated MAIT cells display functional duality, secreting cytokines with either antitumor (IFN‐γ, TNF) or protumor (IL‐17α, IL‐22, IL‐13) properties [257, 267]. This pleiotropic nature complicates assessment of their net impact on antitumor immunity, despite their evident activation within the tumor milieu.

In HCC, MAIT cells exemplify remarkable functional plasticity, though their prognostic significance remains contested. While some investigations associate abundant MAIT infiltration with favorable clinical outcomes [260], others report correlation with poor prognosis [268]. Detailed characterization reveals that intratumoral MAIT cells in HCC exhibit upregulated inhibitory receptor expression (PD‐1, cytotoxic T‐lymphocyte‐associated protein 4 [CTLA‐4], TIM‐3) concomitant with impaired production of cytotoxic mediators (IFN‐γ, granzyme B, perforin) compared with their peritumoral counterparts (Figure 5E). This exhausted phenotype appears principally mediated by TAMs within the immunosuppressive microenvironment. TAMs drive MAIT cell dysfunction via elevated expression of CSF1R, PD‐L1, and CD69, with direct cellular interactions ultimately suppressing MAIT cell effector functions and promoting functional exhaustion [269].

*γδT Cells*: γδT cells represent an unconventional lymphocyte population that operates independently of MHC restriction and demonstrates strong associations with innate immunity. These cells exhibit preferential homing to epithelial‐rich environments including the intestinal mucosa, cutaneous tissues, and pulmonary epithelium. Their antigen recognition capability encompasses MHC‐independent nonpeptide ligands upregulated during cellular stress responses.

γδT cells exert their antitumor cytotoxicity through multiple effector mechanisms, predominantly through coordinated secretion of IFN‐γ together with perforin and granzyme family proteins [270]. Conversely, under specific microenvironmental conditions, these cells undergo polarization toward protumor phenotypes, largely mediated by IL‐17A production. Accumulating evidence from both murine models and human cancer specimens has established the protumor characteristics of the γδT17 subpopulation. Mechanistic investigations further identify MDSCs as critical downstream mediators executing γδT17‐driven tumor promotion.

In breast cancer, tumor‐associated mesenchymal stem cell‐derived exosomes drive the differentiation of monocytic MDSCs (M‐MDSCs) toward highly immunosuppressive TAMs characterized by elevated PD‐L1 and CD206 expression, along with enhanced Arg‐1 activity [271]. Intriguingly, γδT cells have been identified as essential drivers of immunosuppressive macrophage polarization in nonmalignant experimental lung inflammation models [272]. Conversely, within neoplastic microenvironments, MDSCs, mesenchymal stromal cells, immunosuppressive TAMs, and Tregs collectively impair γδT cell infiltration and cytotoxic function through coordinated suppression of IFN‐γ production and induction of immunosuppressive mediator release [273, 274, 275].

#### 3.4.5 Myeloid‐Derived Suppressor Cells

MDSCs comprise a heterogeneous population of innate immune cells originating from myeloid precursors at various developmental stages. Under physiological conditions, these cells undergo normal differentiation into DCs, macrophages, and granulocytes. However, in pathological states, including inflammatory conditions, tissue injury, neoplasms, and autoimmune disorders, the release of immunosuppressive mediators disrupts myeloid progenitor differentiation, promotes their expansion, and facilitates their recruitment to peripheral blood, spleen, liver, and tumor tissues [276, 277, 278]. The aberrant differentiation and functional alteration of myeloid cells represents a recognized hallmark of cancer. Tumors frequently exhibit accumulation of relatively immature, pathologically activated MDSCs possessing potent immunosuppressive capabilities. These cells support tumor progression through multiple mechanisms: enhancing tumor cell survival, stimulating angiogenesis, facilitating tissue invasion, and metastasis [279].

There are two different types of MDSC, as identified in studies in both mice and humans: polymorphonuclear MDSC (PMN‐MDSC) are morphologically and phenotypically similar to neutrophils, whereas M‐MDSC are similar to monocytes. Accumulating evidence indicates distinct functional specialization between MDSCs residing in peripheral lymphoid organs versus those infiltrating tumor tissues. In peripheral lymphoid organs, MDSCs are predominantly represented by PMN‐MDSCs exhibiting moderate immunosuppressive activity, primarily functioning to regulate tumor‐specific immune responses and ultimately induce T‐cell tolerance [280, 281, 282]. Within these sites, the differentiation of M‐MDSCs into macrophages and DCs is substantially impaired. In contrast, tumor‐infiltrating MDSCs display enhanced immunosuppressive capacity, with M‐MDSCs predominating over PMN‐MDSCs and rapidly differentiating into TAMs [283].

#### 3.4.6 Cancer Stem Cell

The rare subpopulation of CSCs serves as the fundamental driver of tumor growth due to their unique tumor‐initiating capacity. CSCs not only initiate primary tumor formation but also metastasize to distant sites to establish secondary tumors [284]. While most disseminated tumor cells fail to complete the metastatic cascade or successfully detach from the primary ECM [285], CSCs overcome these barriers through ECM remodeling, EMT, and intrinsic quiescence, enabling them to survive and adapt throughout this demanding process [286]. However, the pivotal role of CSCs in tumorigenesis, metastasis, and recurrence depends critically on their ability to evade antitumor immune responses and enact self‐serving immune editing. CSCs disrupt immune effector functions within their niche through multiple strategies: they suppress NK cell activity by inducing apoptosis and modulate surface receptors to avoid immune recognition [287, 288]; simultaneously, they evade CD8^+^ cytotoxic T‐cell immune surveillance by downregulating MHC I expression and selectively enhance inhibitory checkpoint receptors to impede T‐cell proliferation [288, 289]. Furthermore, CSCs actively shape a pro‐TME through the recruitment of immunosuppressive cell populations such as MDSCs and Tregs. These recruited cells secrete specific cytokines and drive metabolic reprogramming within the niche, which not only helps maintain CSC properties but also grants them a selective survival advantage [290, 291]. The presence and spatial distribution of TAMs are positively correlated with CSCs. Comprehensive analyses confirm that both TAMs infiltration density and their physical proximity to CSCs directly correlate with histological grade and CSC prevalence [292]. Furthermore, TAMs demonstrate preferential accumulation within hypoxic TME [293], mirroring the established localization pattern of CSCs within analogous hypoxic niches [294]. Preclinical models substantiate these clinical observations: coimplantation of CSCs with TAMs in immunocompetent syngeneic mice significantly enhances tumor‐initiating capacity and metastatic efficiency [295]. Supporting this reciprocal interplay, xenograft studies document amplified tumorigenicity when HCC CSCs are preconditioned with TAMs prior to transplantation [296, 297].

Further investigations reveal that CSCs actively orchestrate macrophage recruitment through the dynamic secretion of chemotactic mediators, including CCL2, CCL3, CCL5, CCL8, CXCL5, and CXCL12[298, 299]. Across multiple malignancies, CSCs exhibit significantly higher CCL2 expression compared with non‐CSC counterparts. This enhanced CCL2 expression correlates with increased recruitment of TAMs, particularly the M2‐polarized phenotype, to tumor sites, whereas CCL2 blockade effectively impairs the accumulation of M2‐like TAMs [299, 300]. Both CXCL5 and CXCL12 are similarly expressed by CSCs and facilitate TAMs infiltration. CXCL5, functioning downstream of transcription factor Sox9, demonstrates chemotactic activity for both neutrophils and macrophages, while CXCL12 activates CXCR7 to coordinate metastatic progression in breast cancer alongside M2‐like TAMs recruitment [301]. Additional soluble factors including IL‐33, CSF1, and VEGFA contribute to this process, with glioma CSCs documented to release CSF1 and other cytokines that not only recruit macrophages but also polarize them toward an immunosuppressive phenotype [293]. CSC‐derived exosomes also participate in macrophage modulation. For instance, glioblastoma stem cell‐derived exosomes promote monocyte differentiation into protumor M2‐like TAMs through STAT3 transfer—a mechanism known to induce protumor TAMs phenotype [302]. Moreover, CSCs can drive macrophages toward M2‐like TAMs through direct contact‐dependent mechanisms, illustrating the diverse strategies CSCs employ to exert immunomodulatory arm. A reciprocal support exists between M2‐like TAMs and CSCs, wherein TAMs contribute to CSCs maintenance, expansion, and EMT. This symbiotic relationship is further reinforced through shared chemokine signaling; specifically, chemokines secreted by CSCs to recruit TAMs are reciprocally produced by TAMs to promote CSCs function [303]. In breast cancer, TAMs‐derived CCL2 activates the β‐catenin signaling pathway, leading to the upregulation of CSCs‐related stemness genes and the expansion of the ALDH^+^ and CD44^+^ CSCs population [304]. Additionally, TAMs‐derived CCL3 enhances tumor cell migration and invasion [305]. Similarly, in glioblastoma, TAMs‐derived CCL5 and CCL8 drive stemness and invasive phenotypes [306]. In HCC and breast cancer, TAMs‐derived IL‐6 activates the STAT3 pathway to promote CSCs population, migration, and angiogenesis [296, 307]. TAMs also release IL‐10 and IL‐35, cytokines that reinforce the CSC phenotype. For instance, IL‐10 from M2‐like TAMs induces the upregulation of stemness genes in lung cancer. Targeting this pathway represents a promising therapeutic strategy.

In summary, the intricate and multifaceted interactions between TAMs and various immune cell populations highlight their pivotal role in establishing the immunosuppressive TME. A deeper understanding of these crosstalk mechanisms will guide the rational development of combination therapies that simultaneously target TAMs and other key immune components, thereby promoting sustained antitumor immunity.

## 4 TAMs‐Targeted Therapeutic Strategies

Given the pivotal role of TAMs in establishing and maintaining a tumor‐supportive microenvironment, therapeutic strategies aimed at neutralizing their protumor functions are critical for enhancing cancer treatment outcomes. Therapeutic interventions targeting TAMs can synergistically restore antitumor immunity by dismantling immunosuppressive networks, unleashing macrophage phagocytic capacity, and reestablishing a more immunogenic TME (Table 2).

**TABLE 2:** Clinical trials targeting tams.

Target	Drug	Clinical trials	Tumor type	References
CCR2/CCL2	Carlumab TD‐92	Preclinical Preclinical	Glioblastoma Solid tumor	[308, 309]
	M2pep	Preclinical	Solid tumor	[310]
CXCR4/CXCL12	Plerixafor	NCT00103610	Lymphoma	[311]
	BL‐8040	NCT02826486	AML	[312]
CSF1/CSF1R	3D185 PLX3397	NCT00786201 NCT02371369	Solid tumor Solid tumor	[313, 314]
TLR agonist	MGN1703	NCT02077868	Colorectal cancer	[315]
CAR‐M therapy	CT‐0508	NCT04660929	Solid tumor	[316]
	MCY‐M11	NCT04863014	Ovarian cancer	[317]
CAF	Sibrotuzumab	NCT00004074	NSCLC	[318]
Immune checkpoint inhibitors	Nivolumab	NCT02558894	HCC	[319]
	Durvalumab	NCT02125461	NSCLC	[320]
	Relatlimab	NCT03470922	Melanoma	[321]
Combination therapy	SNDX‐6352 Atezolizumab	NCT04301778 NCT02323191	HCC Solid tumors	[322, 323]

*Note*: Data resources come from https://clinicaltrials.gov, covering a time span of approximately 5 years.

### 4.1 Depletion or Inhibition of TAMs Recruitment

**FIGURE 6:**
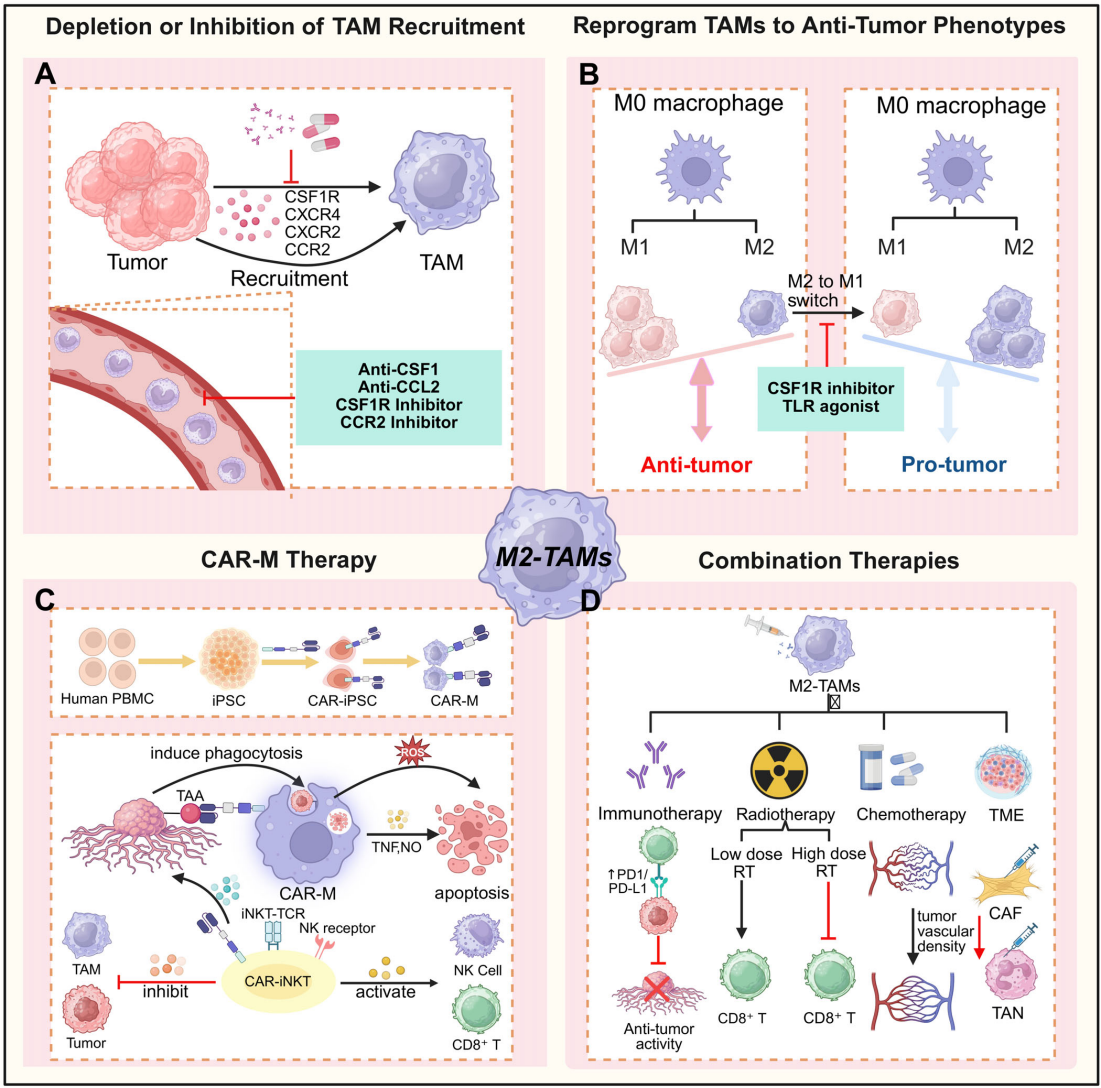
Therapeutic strategies targeting M2–TAMs. M2–TAMs‐targeted strategies in tumors. TAMs‐targeted strategies can be roughly divided as follows: (A) tumors secrete factors such as CSF1 and CCL2 to recruit TAMs. Therapeutic agents including anti‐CSF1, anti‐CCL2, CSF1R inhibitors, and CCR2 inhibitors can block these signaling axes, reducing TAMs accumulation in the TME. (B) Macrophages can polarize into either protumor (M2) or antitumor (M1) TAMs. Agents like CSF1R inhibitors and TLR agonists drive M2‐to‐M1 switching, redirecting TAMs toward an antitumor functional state. (C) CAR‐Ms are engineered by introducing CARs into iPSCs or PBMCs, enabling specific recognition and elimination of tumor cells. CAR‐M recognize tumor‐associated antigens (TAAs) to induce tumor phagocytosis, secrete proinflammatory cytokines (e.g., TNF‐α, NO), and promote tumor apoptosis. CAR‐NKT (equipped with iNKT‐TCR/NK receptors) can activate NK cells and CD8+ T cells, targeting both TAMs and tumor cells. (D) Strategies targeting M2–TAMs can synergize with other therapies: combination with immunotherapy: anti‐PD‐1/PD‐L1 blocks TAMs‐mediated T cell suppression. Combination with radiotherapy (RT): low‐dose RT enhances CD8+ T cell function, while high‐dose RT inhibits CD8+ T cell function. Combination with chemotherapy: reduces tumor vascular density. Combination with targeting other TME components: targeting cancer‐associated fibroblasts (CAFs) and tumor‐associated neutrophils (TANs).

The polarization and infiltration of TAMs are coordinately regulated by diverse microenvironmental mediators, including cytokines, chemokines, and growth factors originating from both neoplastic and stromal cellular components [21]. CSF1 and CCL2 represent two extensively characterized chemotactic factors that mediate macrophage recruitment and strongly promote M2‐polarized activation states. Therapeutic blockade of CCL2/CCR2 or CSF1/CSF1R signaling axis through small molecular inhibitors and monoclonal antibodies have emerged as promising approaches for attenuating TAMs‐mediated immunosuppression. Monoclonal antibodies and small‐molecule inhibitors targeting CSF1R have demonstrated significant reductions in TAMs abundance alongside increased CD8^+^/CD4^+^ T cell ratios [324] (Figure 6A). For instance, TD‐92, an erlotinib derivative, depletes TAMs while enhancing the efficacy of anti‐PD‐1 therapy [325]. Similarly, due to its pivotal role in mediating monocyte migration, the CCR2/CCL2 pathway emerges as a therapeutically attractive target. Inhibition of this pathway suppresses tumor growth, reduces M2‐like TAMs infiltration at both primary and metastatic sites, and promotes antitumor T cell responses [326, 327]. In glioblastoma models, CCR2/CCL2 blockade diminishes M2‐like TAMs infiltration, improves survival outcomes, and enhances the efficacy of combination therapies [328]. However, nonselective macrophage depletion raises safety concerns, as macrophages are vital for immune defense and tissue homeostasis [329]. To mitigate off‐target effects, current research focuses on developing strategies for selective targeting of M2‐like TAMs subpopulations. For instance, the M2 macrophage‐targeting peptide (M2pep) selectively eliminates M2‐like TAMs and tumor cells while preserving M1 subsets in preclinical models [330, 331, 332]. Although still under investigation, M2pep holds promise as a targeted adjunctive therapy pending further clinical validation.

### 4.2 Reprogramming TAMs to Antitumor Phenotypes

While depleting macrophages may offer short‐term benefits, such nonselective elimination carries significant risks, including notable deficits in infection resistance and disruption of tissue homeostasis. Therefore, reprogramming protumor M2‐like TAMs into antitumor M1‐like TAMs represents a more advantageous and potentially effective strategy (Figure 6B).

CSF1R inhibitor, beyond suppressing TAMs recruitment, have demonstrated the capacity to reprogram existing M2‐like TAMs toward an M1 phenotype. This phenotypic shift correlates with restrained tumor growth, enhanced cytotoxic T cell responses, and superior treatment outcomes when administered in combined with chemotherapy [333]. For instance, PLX3397 demonstrates antitumor activity by modulating TAMs polarization rather than depleting macrophage populations [314, 334]. Similarly, 3D185, a novel small‐molecule CSF1R inhibitor, has shown potent effects on TAMs reprogramming and tumor suppression in preclinical models [313]. TLRs serve as potent regulators of macrophage plasticity. Stimulation of immunostimulatory TLRs (TLR3/7/8/9) reverses M2‐like polarization, restoring M1‐like antitumor functionality [335, 336]. For instance, lefitolimod (MGN1703), a synthetic TLR9 agonist, remodels the immunosuppressive TME by enhancing cytotoxic CD8^+^ T cell infiltration and promoting immunostimulatory TAMs polarization [337]. Furthermore, exosomes derived from M1‐like TAMs can induce phenotypic shift of TAMs from an M2 to M1 phenotype, underscoring their promising role in TAMs reprogramming strategies [338].

### 4.3 Adoptive Cell Therapy With Engineered Macrophages

ECM, composed of highly organized macromolecular proteins, fiber molecules, proteoglycans, glycoproteins, and glycosaminoglycans, presents a significant physical barrier to various antitumor therapies, including chimeric antigen receptor (CAR)‐T and CAR‐NK treatments [339]. In contrast, macrophages demonstrate a unique capacity to infiltrate the TME through secretion of diverse MMPs that facilitate ECM degradation [340]. CAR‐macrophage (CAR‐M) present strategic advantages for TME reprogramming and systemic immune activation through their inherent phagocytic capacity and antigen presentation functions, potentially enabling durable tumor control [341]. This promising premise has spurred the exploration of CAR‐M as an innovative therapeutic platform. By genetically engineering macrophages, which are naturally versatile mediators of tissue homeostasis, inflammatory regulation, and immune surveillance [342], to express CARs, this approach directly exploits their innate phagocytic capacity and tumor‐microenvironment‐modulating potential for targeting solid tumors [343, 344]. Notwithstanding these theoretical benefits, substantial debate persists concerning CAR‐M therapeutic efficacy, safety profiles, and clinical implementation [345, 346]. Current understanding remains constrained, particularly regarding direct clinical outcomes and mechanistic insights into CAR‐M therapy across heterogeneous TME contexts [347, 348].

#### 4.3.1 The Mechanisms of CAR‐M Immunotherapy

CARs, artificial transmembrane receptors designed to target specific tumor antigens, have revolutionized adoptive cell therapy, particularly in hematologic malignancies [349, 350, 351]. Despite this success, CAR‐T therapies encounter therapeutic hurdles including limited efficacy in solid tumors, short persistence, and potential off‐target effects. Given their innate capabilities, including potent phagocytic activity, antigen presentation, cytokine secretion, and enhanced tumor infiltration, macrophages represent a compelling alternative platform for CAR‐based engineering [352, 353]. This innovation has positioned CAR‐M therapy as a frontrunner in next‐generation solid tumor immunotherapy [354, 355]. Preclinical studies have demonstrated that CAR‐M therapies effectively eliminate tumor cells both in vitro and in vivo through multiple mechanisms. CAR‐M exert direct cytotoxicity by secreting tumoricidal molecules such as TNF, NO, and ROS, which synergistically induce tumor cell apoptosis (Figure 6C). For example, Duan et al. designed a CAR‐M targeting VEGF receptor‐2 (VEGFR2) that, when activated by TLR4 and/or IFN‐γ signaling, produces high levels of TNF‐α, leading to significant tumor suppression [356]. Moreover, CAR‐M can induce tumor cell lysis via ADCC and ADP. By recognizing and binding to the antibody‐coated tumor cells through FcRs, CAR‐M can release cytotoxic substances or directly engulf and digest tumor cells.

Macrophages also orchestrate tumor eradication through dynamic crosstalk with other immune cells. Studies demonstrate that CAR‐M function not only as antigen‐presenting cells to prime tumor‐specific T cells, but also actively recruit multiple immune effectors into the TME. By secreting chemokines such as CXCL8 and CCL5, CAR‐M enhance the infiltration of antitumor neutrophils, Th1, Th17, CTLs, and NK cells, thereby amplifying coordinated antitumor immunity [357]. Furthermore, single‐cell sequencing analyses reveal that CAR‐M maintain an M1 phenotype within tumor tissue while exhibiting elevated expression of inflammation‐associated genes [358]. Activated M1 macrophages release proinflammatory cytokines such as IL‐1β, IL‐6, IL‐12, IL‐18, and TNF, which collectively establish a proinflammatory microenvironment and stimulate T cell activation, demonstrating comprehensive and coordinated immune functionality [359, 360]. Studies report that CAR‐M targeting HER2 and CD47 can operate synergistically with CD8^+^ T cells, inducing cytokine secretion that targets ovarian cancer cells in vitro [361].

Encouraging preclinical results have accelerated clinical interest, with multiple ongoing clinical trials are evaluating the safety profile and therapeutic potential of CAR‐M therapies for solid tumors. However, challenges remain, including limited antitumor potency, restricted proliferative capacity, high plasticity, and potential offer‐target toxicity [362, 363]. Future advancement in tumor immunology and CAR engineering will be critical for developing sophisticated next‐generation CAR‐M therapies with improved designs, manufacturing protocols, and combination strategies.

#### 4.3.2 Perspective on the Next Generation of CAR‐M

Advances in tumor immunology and CAR technology are driving the development of increasingly sophisticated next‐generation CAR‐M therapeutics for clinical evaluation. Critical considerations for enhancing CAR‐M therapies include refining CAR designs to maximize phagocytic efficiency, implementing combination strategies to achieve synergistic effects, streamlining manufacturing protocols, and developing allogeneic or “off‐the‐shelf” therapeutic products [345, 364].

Ongoing research has dedicated significant resources to improving the fundamental composition of CAR molecules, including the hinge and transmembrane regions, to bolster the mechanical strength of CAR molecules and augment the antitumor capabilities of CAR‐M. An examination centered on the interaction between CAR and antigen has the potential to facilitate the identification of CAR cells that possess unique sensitivities toward tumor recognition and function, ultimately paving the way for controlled immunotherapy for cancer through drug intervention [365,366]. New generations of CAR molecules are currently developed to harbor multiple tandem functional units that encompass specific cytokines, transcription factors, activating signaling molecules, inhibitors of phagocytosis checkpoint, and so on. With the new discovery of the epigenetic and metabolic regulators key for macrophage robustness and fitness [367, 368], CAR therapeutic will be armed with more layers of regulatory machinery that are inaccessible for traditional therapeutics. By integrating a comprehensive regulatory network, scientists endeavor to form the AND, OR, and NOT logic gates in new CAR designs to develop a smarter and precisely controlled living therapeutic material [369, 370].

Evidence has shown that induced pluripotent stem cell (iPSC)‐derived macrophages enable the scalable production of therapeutic cells, although further efforts are needed to improve and standardize the manufacturing protocol for the production of clinical‐grade products [371]. Additionally, as pluripotent cells are generally amenable to genetic manipulations, an expanding spectrum of molecules central to CAR‐M efficacy are edited and optimized to develop new therapeutics with customized sense‐and‐respond modules [372, 373]. In this regard, the application of innovative technology such as synthetic biology, in situ gene editing, and mRNA‐based cell engineering would provide a potent, effective, and flexible platform technology [363, 371, 374]. At the same time, biomaterials have demonstrated the capability to improve the effectiveness of engineered cells by transporting therapeutic carriers (referred to as “backpacks”), or facilitating in situ CAR editing through the delivery of mRNA, DNA, and CRISPR‐based gene‐editing systems [375]. Considering the uniqueness of biomaterials in translating the disease inputs such as pH, light, and hypoxia into functional outputs like cleavage, gel–sol transition, and conformational change, it is expected that marrying biomaterials with CAR technology will foster smarter, controllable, and cost‐effective immunotherapy [376].

Given the combined effect of the innate and adaptive immune response to cancer, it is logical to develop CAR‐M alongside CAR‐T cells, particularly those with increased immunogenicity through tumor vaccines. Recent data have shown that vaccine‐boosted CAR‐T cells exhibited the robust ability to exuberantly release IFN‐γ, deliver the costimulatory signals to and activate innate immune cells, setting the basis for improved immunotherapy [377]. Furthermore, it is worth exploring the potential of investigating the combination of CAR‐M with conventional therapies like chemotherapy, radiation, and other targeted drugs. These treatments have the potential to stimulate the release of tumor antigens and damage‐associated molecular patterns when eliminating cancer cells, leading to immunogenic cell death and effectively reactivating intratumoral macrophages [378, 379]. Thus, a well‐organized CAR‐M regimen may include orchestrating multiple therapeutics with optimized doses, schedules, and agents to achieve maximized antitumoral potential and patients’ well‐being [380].

Recent studies demonstrate that CAR‐NKT cells can simultaneously target tumor cells and TAMs, leading to enhanced antitumor efficacy. NKT cells express both TCRs and NK‐cell receptors on their surface and can produce a broad array of cytokines, including IL‐4 and IFN‐γ. These cells not only directly lyse and kill tumor cells but also exert antitumor effects by secreting cytokines and activating other immune cells [381, 382]. NKT cells demonstrate broad‐spectrum antitumor activity and can infiltrate the TME to eliminate TAMs, which are immune cells known to promote tumor growth and metastasis [383, 384]. Researchers are currently exploring the development of novel immunotherapies using engineered NKT cells for cancer patients. For instance, genetic modification of human NKT cells with CARs has enabled specific recognition and attack of neuroblastoma. The modified NKT cells can be further engineered to express IL‐15, a natural protein that supports NKT‐cell survival [385, 386].

### 4.4 Metabolic Reprogramming of TAMs as an Antitumor Therapy

Metabolic adaptations in macrophages govern their viability, differentiation, phenotypic polarization, and trafficking to TME. Given that tumor‐derived metabolites critically orchestrate macrophage reprogramming, a process fundamental to their effector functions, it follows that targeting metabolic pathways represents a compelling therapeutic strategy to reverse macrophage phenotypes in malignancy.

To maintain their accelerated proliferation, neoplastic cells undergo profound metabolic reprogramming characterized by dysregulated glucose metabolism—a recognized hallmark of malignancy. Consequently, targeting these aberrant glycolytic pathways represents a promising strategy to suppress oncogenesis. A key step in cancer glycolysis is the uptake of glucose, facilitated by glucose transporters (GLUTs) across the cell membrane [387]. The GLUT family members GLUT1, GLUT2, GLUT3, and GLUT4 have distinct physiological roles depending on the cell type and substrate [388]. GLUT1 is frequently overexpressed in cancers, including renal cell carcinomas with VHL gene loss. Inhibiting GLUT1 with specific small molecules has been shown to selectively kill these cancer cells. Cytochalasin B, the first identified GLUT1 inhibitor, blocks GLUT by binding to the transporter's internal site [389].

Hexokinase 2 (HK2) serves as a pivotal enzyme that initiates glycolysis in cancer cells by catalyzing the phosphorylation of glucose to glucose‐6‐phosphate. HK2 is broadly expressed in metabolically active tissues, with notably elevated levels observed in malignant cells. Its primary functions encompass catalyzing glycolysis, suppressing apoptosis, and reprogramming tumor metabolism [390]. Currently, the development of targeted HK2 inhibitors remains in a nascent phase. In experimental settings, small molecules including 2‐DG, 3‐bromopyruvate, lonidamine, and metformin have been extensively investigated as potential HK2 inhibitors. While these agents have demonstrated promising antitumor efficacy in cellular and animal models, their clinical translation continues to pose significant challenges.

The lipogenic phenotype represents a fundamental metabolic hallmark of malignancies [391], characterized by upregulated expression of lipogenic enzymes and key regulators governing de novo fatty acid synthesis in tumor cells, thereby conferring proliferative advantages [392]. Recent advances in lipid metabolism research have revealed promising immunomodulatory strategies through targeting fatty acid synthesis and catabolism in TAMs. Within the TME, PD‐1 immune checkpoint signaling promotes alternative activation of TAMs toward the M2 phenotype while concurrently suppressing their phagocytic capacity [44]. In mouse models of melanoma and colon cancer, cholesterol regulatory element binding protein 1 (SREBP1) mediates ab initio synthesis of fatty acids in M2‐like TAMs. Fatostatin, an SREBP1 inhibitor, selectively inhibits the activation of M2‐like TAMs and activates CD8^ +^ T cells, triggering a positive feedback loop favoring immune stimulation of the TME. The combination of fatostatin with anti‐PD‐1 drugs can increase the blocking efficiency of ICIs for tumor growth inhibition [393]. Carnitine palmitoyl transferase (CPT), a key rate‐limiting enzyme of FAO, can bind fatty acids and carnitine into the mitochondria, thereby regulating FAO and promoting the metabolic response in cancer. Etomoxir (a CPT‐1A inhibitor) inhibits FAO‐dependent ROS and NOD‐like receptor thermoprotein structural domain‐associated protein 3 in M2‐like TAMs, which in turn inhibits IL‐1β secretion and limits TAMs‐mediated tumor cell migration [394]. In mouse models of lung and CRC, the use of FAO inhibitors enhanced the antitumor effects of pericyte therapy by suppressing tumor‐infiltrating MDSC‐cell‐mediated immunosuppression [395].

Glutamine functions as a conditionally essential amino acid that assumes critical importance under pathological conditions and physiological stress [396]. Glutamine in cancer cells plays critical and diverse roles by providing not only a source of nitrogen for amino acid and nucleotide biosynthesis but also a source of carbon to replenish the tricarboxylic acid cycle and lipid biosynthesis pathways; thus, cancer cells are “addicted” to glutamine [397]. The regulatory influence of glutamine metabolism extends to macrophage differentiation, where it directs metabolic reprogramming during polarization toward antitumor (M1) or protumor (M2) phenotypes. TAMs exhibit remarkable functional plasticity along the M1–M2 polarization continuum. Glutamine restriction selectively disrupts M2 polarization through suppression of UDP‐GlcNAc biosynthesis and consequent impairment of N‐glycosylation for M2‐associated surface markers (including Relmα, CD206, and CD301), while preserving M1 phenotypic characteristics [398]. Consistent with this, glutaminolysis‐derived α‐KG promotes M2 activation by increasing FAO and Jmjd3‐dependent epigenetic reprogramming of M2‐related genes [399]. In contrast to the inhibition of glutaminolysis, pharmacological, or genetic targeting of GLUL in macrophages reprograms M2‐polarized macrophages to an M1‐polarized phenotype [400]. Glutamine metabolism and closely linked metabolic networks involving glutamine transporters, glutaminase, aminotransferase, and redox homeostasis are essential for cancer cell survival [401]. Targeting each step of glutamine metabolism has shown promising results in cancer treatment, prompting the discovery of druggable targets and the development of antitumor drug candidates [402].

In conclusion, clinical innovations targeting tumor metabolic reprogramming will become a major focus in cancer therapy research. However, at present, there are still challenges and limitations associated with drugs or therapeutic approaches targeting tumor metabolic reprogramming. We should conduct more basic and clinical studies to maximize their benefits in treating human cancers.

### 4.5 Exosome‐Based Tumor Suppression Strategies

Exosomes play a critical role in driving cancer progression and metastasis by enabling the transfer of genetic material and proteins between cancer cells and their surrounding microenvironment. At the same time, these naturally occurring vesicles offer a valuable platform for therapeutic development, including novel approaches to cancer treatment. The wide range of molecules and functional properties that exosomes can carry makes them a promising target for innovative cancer therapies. A deeper understanding of the mechanisms by which exosomes influence tumor initiation, progression, and drug resistance, coupled with the creation of targeted therapies, is anticipated to lead to significant advances in cancer treatment.

#### 4.5.1 Exosomes as Biomarker

Exosomes, found in various bodily fluids, contain nucleic acids, proteins, metabolites, and lipids derived from their original cells. These contents make them valuable indicators of cellular health and disease [403]. In clinical samples like plasma, serum, urine, and saliva, exosomes have been used to determine a patient's diagnosis, prognosis, disease progression, and response to chemotherapy. Importantly, increased exosome release is often associated with complex diseases, including inflammation and cancer. During disease, changes in cells can be monitored by analyzing the exosomes they shed. Differences in the levels of specific molecules can be identified and measured using transcriptomic, proteomic, and lipidomic techniques [404]. The application of exosomes in diagnostics is especially promising for the early discovery and outcome prediction of inflammatory diseases and cancer. For example, miRNAs from plasma exosomes in patients have been recognized as potential biomarkers for immunotherapy in NSCLC. A comparative study of patients with advanced EGFR/ALK wild‐type NSCLC treated with PD‐1/PD‐L1 inhibitors revealed over 150 differentially expressed exosomal microRNAs compared with healthy individuals. Specifically, low levels of exosomal has‐miR‐320d, hsa‐miR‐320c, and hsa‐miR‐320b were associated with better response to immunotherapy. Additionally, when exosomal hsa‐miR‐125b‐5p, which suppresses T cells, is reduced during treatment, patients may experience improved T‐cell function and a positive therapeutic outcome [403].

#### 4.5.2 Exosomes as Therapeutic Agents

Exosomes are gaining significant attention as therapeutic tools because of their biocompatibility, natural targeting capabilities, and ability to carry diverse bioactive cargo like RNA, proteins, and lipids [405]. They efficiently deliver these molecules to specific cells, modulating cellular activities, which shows great promise for gene therapy, immune modulation, and tissue repair [406]. Moreover, exosomes can be engineered to carry precise therapeutic agents, such as RNA‐based therapies, protein drugs, and antibodies, to enhance their treatment potential. Research into using exosomes to treat acute and chronic inflammation, as well as tumors, is actively underway. In summary, due to their broad potential as therapeutic agents, exosomes are poised to become a new class of precision medicine tools.

Focusing on the biogenesis and molecular composition of macrophage‐derived exosomes could yield critical insights for antitumor therapies [407]. Limiting the production and release of such exosomes may exert antitumor effects. For instance, PD‐L1 is a key immune‐evasion protein exploited by tumors; macrophages can promote PD‐L1 expression in cancer cells through exosomes‐mediated signaling. However, treatment of macrophages with GW4869, an inhibitor of exosomes release, blocks this process [408, 409]. It is important to recognize that systemic inhibition of exosomes formation could also disrupt physiological intercellular communication and function, potentially causing adverse effects. Therefore, therapeutic strategies targeting exosomes biogenesis require careful risk‐benefit evaluation. Regarding exosomes cargo, a compound called ovatodiolide has been shown to reduce levels of miR‐21 in exosomes from M2‐like TAMs, thereby inhibiting the development of bladder cancer [409]. This suggests that targeting specific miRNAs in macrophage‐derived exosomes could have clinical utility. Overall, in designing clinical trials for therapies based on macrophage exosomes, it is crucial to balance maximizing treatment benefits with minimizing potential adverse effects.

#### 4.5.3 Exosomes as Therapeutic Carries

Exosomes are emerging as promising natural delivery platforms, capable of carrying therapeutic payloads while preserving their intrinsic biological properties. Their stable architecture enables the loading of drugs through physical, chemical, or biological techniques, and their lipid bilayer protects encapsulated agents from enzymatic degradation such as by RNase, thereby reducing premature loss [410]. Exosomes can be administered through various routes, including intranasal, intravenous, intraperitoneal, and intracranial, and their ability to cross the blood–brain barrier (BBB) enables drug delivery to the brain [411]. As a nontoxic option, exosomes generally have lower immunogenicity and superior pharmacokinetic and pharmacodynamic profiles compared with synthetic nanoparticles [412]. These advantages have established exosomes as a major focus in drug delivery system research. The IL‐4 receptor (IL‐4R) expression was higher in M2‐like TAMs than in antitumor M1‐like TAMs. Gunassekaran et al. constructed a vector using the IL‐4R‐binding peptide IL‐4R Pep‐1 to target this specific receptor and successfully developed IL‐4R‐exosome. This modified exosome vector showed a strong efficacy in reducing the expression of M2‐like TAMs target genes. Furthermore, whole‐body fluorescence imaging showed that IL‐4R‐exosome could be significantly aggregated in tumor tissues, and could inhibit tumor growth, reduce M2‐like TAMs, and increase M1‐like TAMs. Reprogramming TAMs into M1‐like TAMs and improving antitumor immune response can effectively inhibit tumor progression, which provides strong support for tumor immunotherapy [413]. However, there are several challenges that need to be overcome to fully realize the potential of exosomes‐based therapies in cancer patients. These include the optimization of exosomes production and engineering, the identification of suitable cancer targets, and the development of strategies to overcome drug resistance and tumor heterogeneity [414]. This could allow for noninvasive monitoring of treatment response and disease progression. Overall, therapeutic targeting of exosomes holds great promise as a novel approach to cancer treatment, offering the potential for more effective and personalized therapies. With the continuous progress of science and technology and the deepening of clinical trials, we believe that exosomes will become one of the important tools for cancer treatment in the future.

### 4.6 Combination Therapies

TAMs do not act in isolation but interact dynamically with multiple components of the TME, including fibroblasts, epithelial cells, neutrophils, mesenchymal stem cells, MDSCs, and mast cells. Preclinical studies that examine TAMs alone, without accounting for the intricate signaling networks that connect these cellular populations, often produce results that do not translate effectively to the clinic. Therefore, research should focus on clarifying the specific contributions of each TME element and apply systems‐biology frameworks to model their evolving crosstalk during tumor progression and metastasis. Such integrated approaches may reveal new therapeutic opportunities [415]. Moreover, combining TAMs‐targeted interventions with other treatment modalities, such as chemotherapy, ICIs, and RT, generally produces better outcomes than monotherapy alone (Figure 6D).

#### 4.6.1 Targeting Multiple TME Components

*Targeting CAFs*. CAFs constitute a major stromal cell population in the TME and play a central role in driving tumor growth, invasion, and immune evasion [416, 417]. Unlike normal fibroblasts, CAFs remain in a perpetually activated state, resisting reversion to a quiescent phenotype or clearance through apoptosis [418, 419]. They secrete a range of growth factors (such as hepatocyte growth factor and FGF) and chemokines that not only enhance tumor cell proliferation and survival but also recruit additional stromal and immune cells into the tumor niche [420, 421]. Given their abundance and functional significance, CAFs have emerged as attractive therapeutic targets. For instance, fibroblast activation protein α (FAPα), a membrane‐bound serine protease selectively expressed in tumor stroma, has been identified as a therapeutically viable candidate for CAF‐targeted therapies. However, early‐phase clinical trials (Phase I/II) of FAP‐targeted antibodies (e.g., sibrotuzumab) demonstrated limited efficacy, underscoring the need for further preclinical and clinical research to refine CAF‐targeted strategies [422].

*Targeting Tumor‐Associated Neutrophils*. Tumor‐associated neutrophils (TANs) exhibit functional plasticity similar to TAMs, polarizing into either protumor (N2) or antitumor (N1) phenotypes [423, 424, 425, 426]. TANs actively participate in tumor initiation, metastatic dissemination, and immunosuppression [427, 428], with high intratumoral neutrophil density correlating with poor prognosis across multiple solid tumor types [429]. Therapeutic strategies targeting TANs typically focus on disrupting neutrophil trafficking, expansion, or phenotypic polarization. For instance, CXCR2 acts as a key regulator of neutrophil infiltration and tissue retention. Growing preclinical and clinical data indicate that blocking CXCR2 signaling can hinder neutrophil mobilization, reduce tumor‐promoting inflammation, and improve treatment outcomes [430].

#### 4.6.2 Combination With Conventional Chemotherapy

The combination of TAMs‐targeted interventions with conventional chemotherapy has garnered increasing attention due to the bidirectional interactions between TAMs and chemotherapeutic agents. The impact of TAMs on chemotherapy efficacy demonstrates substantial context‐dependence, varying with tumor type and drug mechanism. For instance, in ovarian cancer, cisplatin treatment can activate macrophages, elevating CCL20 levels and triggering CCR6‐mediated EMT in cancer cells, thereby compromising treatment efficacy [431]. Conversely, paclitaxel (PCX) reprograms M2‐like TAMs toward an M1‐like phenotype through TLR4 signaling, thereby enhancing PCX's antitumor efficacy [432]. M2‐like TAMs are frequently associated with chemotherapy resistance, tumor recurrence, and poor outcomes with standard regimens [433]. Therefore, coadministering TAMs‐modulating agents with chemotherapy may counteract these effects. Notably, inhibition of CSF1R signaling has been shown to increase intratumoral type I interferon production, which synergize with platinum‐based chemotherapies to improve antitumor efficacy [434]. The CSF1R inhibitor SNDX‐6352 functions through competitive binding to the CSF1R, modulating TAMs proliferation, differentiation, and survival mechanisms. Clinical evaluation (NCT04301778) revealed superior efficacy of SNDX‐6352 combined with durvalumab (MEDI4736) versus durvalumab monotherapy in pretreated intrahepatic cholangiocarcinoma patients, demonstrating improved overall survival without significant toxicity escalation, thereby underscoring the therapeutic potential of CSF1R blockade in immuno‐oncology [435].

TAMs infiltration density frequently correlates positively with tumor vascularization [436, 437]. Preclinical evidence indicates that combining chemotherapy with TAMs depletion can reduce tumor vascular density by roughly 50%. Removing TAMs from the TME drives a phenotypic shift in residual perivascular macrophages from proangiogenic to angiostatic states. This shift improves perfusion and enhances chemotherapeutic drug delivery to tumor sites, ultimately amplifying the overall chemotherapy response [438].

#### 4.6.3 Combination With Immunological Checkpoint Inhibitors

Malignant cells evade immune surveillance by expressing PD‐L1, which suppresses CTLs activity. TAMs play a key role in modulating PD‐L1 expression on tumor cells, establishing a TAMs‐dependent PD‐1/PD‐L1 axis that represents an attractive target for improving ICIs efficacy. Hypoxia and M2‐like TAMs polarization both drive PD‐L1 upregulation on tumor and immune cells, thereby impairing immunotherapy responses, as seen in NSCLCs [439]. Furthermore, TAMs themselves express PD‐L1, reinforcing their immunosuppressive phenotype and directly suppressing T cell function through PD‐1/PD‐L1 axis [440, 441]. Blocking of PD‐L1 not only reactivates exhausted T cells but also reprograms TAMs from an M2‐like to an M1‐like phenotype, restoring their antitumor potential. Therefore, combining TAMs‐targeted therapies with ICIs can synergistically enhance treatment outcomes and offers a promising approach to overcome resistance in solid tumors. In a clinical trial (NCT02323191), the CSF1R inhibitor emactuzumab was evaluated together with the PD‐L1‐blocking antibody atezolizumab in patients with advanced solid malignancies. The study assessed safety, pharmacokinetics, and clinical efficacy of the combination. Results showed a confirmed objective response rate of 12.5% in NSCLC patients who had previously received ICIs. The emactuzumab–atezolizumab combination exhibited an acceptable safety profile, supporting dual targeting of CSF1R and PD‐L1 as a therapeutically viable strategy [323].

#### 4.6.4 Combination With Radiotherapy

The combination of TAMs‐targeted strategies with radiotherapy (RT) holds significant clinical implications. The effect of RT on macrophage polarization remains an area of conflicting evidence. Klug et al. demonstrated in murine breast cancer models and human pancreatic cancer specimens that low‐dose RT (2 Gy) can repolarize M2‐like TAMs toward an M1‐like phenotype through induction of iNOS. iNOS expression correlated with vascular normalization and an influx of CD8^+^ T cells, indicating that repolarized macrophages exert both antitumor and proinflammatory effects. Upregulation of iNOS was also observed in murine prostate cancer following irradiation at doses up to 25 Gy [442, 443]. Similarly, TLR agonists further stimulated the M1 phenotype of macrophages and enhanced the antitumor effects of RT in a murine breast cancer model. Conversely, other studies have reported that tumor irradiation is associated with a more immunosuppressive TAMs phenotype. Jones et al. found that depleting macrophages with an anti‐CSF antibody markedly increased tumor ablation after irradiation (10 Gy) in murine tumors derived from colorectal and pancreatic cell lines [444]. Clearly, the impact of RT on TAMs appears to depend on multiple variables, including the model system, radiation dose, tissue type, and the time point examined after irradiation. Despite these conflicting reports, a pivotal role for TAMs in modulating the response to RT is well established.

In summary, rational combination therapies that cotarget TAMs and other TME components, or integrate TAMs modulation with established treatment modalities, hold great promise for maximizing antitumor efficacy and minimizing therapeutic resistance.

## 5 Clinical Translation

The clinical translation of therapies targeting TAMs has evolved through several phases, marked by both challenges and insights. The first therapeutic attempts aimed at nonspecifically depleting TAMs, for instance with clodronate liposomes. While these efforts proved the concept that targeting macrophages could inhibit tumor growth, the clinical responses were typically incomplete and short‐lived, constrained by both compensatory pathways and insufficient target specificity. The recognition of remarkable TAMs heterogeneity, moving beyond the simplistic M1/M2 dichotomy, has been a critical turning point. Single‐cell RNA sequencing (scRNA‐seq) has been instrumental in deciphering distinct TAMs subpopulations with protumor functions, revealing that targeted elimination of specific subsets can be more effective than broad depletion. Subsequently, more specific biologics emerged, while demonstrating the ability to modulate TAMs populations, its efficacy as a monotherapy was limited, leading to current combinatorial strategies with ICIs (e.g., anti‐PD‐1) to overcome compensatory resistance pathways. The field is now advancing toward even more sophisticated engineered therapies. Bispecific antibodies (e.g., simultaneously targeting CD47 and CD24) potently enhance macrophage phagocytosis. Furthermore, CAR‐M therapy represents a groundbreaking frontier, designed to actively direct macrophages to kill tumors, remodel the TME, and bridge innate and adaptive immunity.

### 5.1 Failures of Previous Clinical Trials Targeting TAMs and Considerations for Future Clinical Trials

In the 1970s, researchers first identified substantial macrophage infiltration within tumors and proposed their dual role in cancer biology [445]. A survey of human data carried out by Underwood et al. demonstrated that the density of macrophages was negatively correlated with patient prognosis [446]. Pioneering work by Van Rooijen et al. used liposomes to deliver drugs in mouse tumor models to selectively deplete TAMs, representing the first TAMs‐targeted clinical trial. This liposome‐encapsulated therapeutic achieved specific macrophage elimination and provided the first demonstration that TAMs depletion could inhibit tumor growth. However, due to the technological constraints of that era, TAMs depletion remained nonspecific, and the absence of reliable biomarkers complicated efficacy evaluation [447]. Building on these foundational studies, later investigations systematically explored the translational potential of TAMs depletion. For instance, Zeisberger et al. examined whether eliminating TAMs could inhibit tumor angiogenesis, progression, and metastasis. Bisphosphonates, well known for preventing skeletal metastases and managing bone turnover disorders, have also shown potential as antiangiogenic agents capable of restraining tumor growth and dissemination [448, 449, 450, 451]. By encapsulating clodrolip, researchers developed a macrophage‐depleting agent that effectively reduced phagocytic cell populations across various preclinical models [452, 453]. In murine F9 teratocarcinomas and human A673 rhabdomyosarcoma xenografts, clodrolip administration achieved 75–92% tumor growth inhibition (varying with treatment protocols) and significantly reduced intratumoral vascular density. These findings positioned clodrolip‐based macrophage depletion as a promising antiangiogenic adjunct, while also highlighting its utility as a research tool for dissecting macrophage and DCs roles in tumor progression. However, the therapeutic effects were incomplete, as tumors exhibited gradual regrowth following treatment discontinuation. This phenomenon is presumably attributed to compensatory proangiogenic activity derived from other stromal components, including mast cells, neutrophils, fibroblasts, additional DCs subpopulations, and pericytes. Consequently, macrophage depletion strategies should be positioned as adjuvant interventions to conventional chemotherapeutic and radiotherapeutic regimens, mirroring the complementary role historically associated with established antiangiogenic approaches [450].

The monocyte‐macrophage lineage exhibits remarkable diversity and plasticity. Early studies simplistically categorize TAMs into M1/M2‐like phenotype, significantly oversimplifying the complex process of macrophage activation. This binary classification fails to account for the combinatorial effects of multiple stimuli on macrophages, resulting in a spectrum of phenotypic diversity and functional complexity that extends far beyond simple polarization extremes. Notably, coexpression of both M1 and M2 associated gene signatures has been detected in macrophage subpopulations across nearly all cancer types [454]. Consequently, a functional spectrum model that correlates phenotypes with biological activities represents a more sophisticated approach for characterizing macrophage subpopulations [455, 456].

**FIGURE 7:**
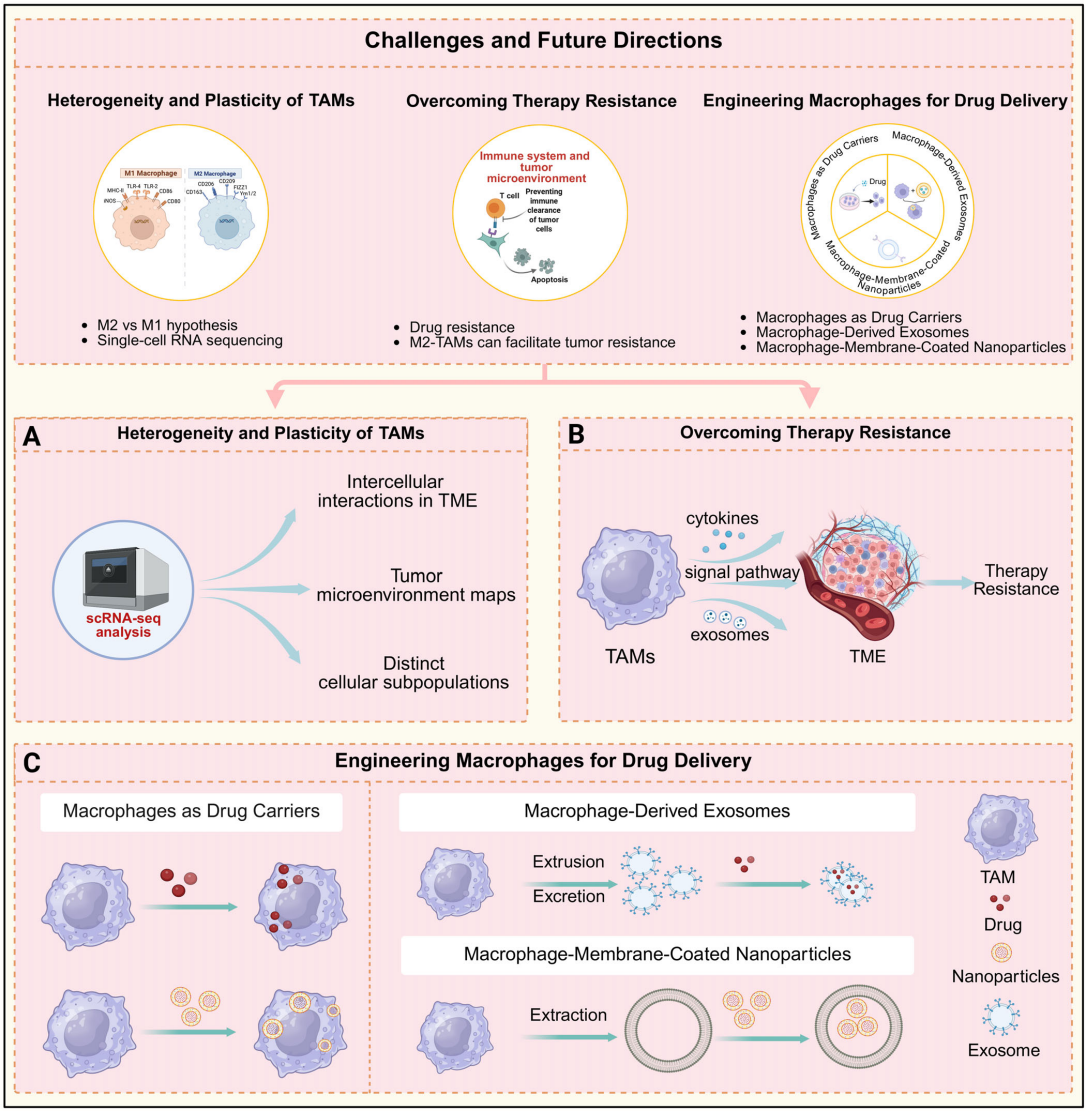
The challenges and future directions in TAMs‐targeted therapy. Challenges and future directions in research related to TAMs, structured into three main areas: (A) ScRNA‐seq analysis unravels intercellular interactions in the TME, constructs TME maps, and identifies distinct TAMs subpopulations, providing a mechanistic framework to understand TAMs functional diversity and guide personalized therapeutic strategies. (B) M2–TAMs secrete cytokines, activate signaling pathways, and release exosomes within the TME to promote tumor therapy resistance. Targeting these M2–TAMs‐mediated molecular cascades is critical for improving the efficacy of existing antitumor therapies. (C) Macrophages as drug carriers: macrophages are loaded with small molecules or nanoparticles to deliver therapeutics directly to the TME; macrophage‐derived exosomes: exosomes isolated from macrophages (through extrusion or excretion) serve as natural vehicles for targeted drug delivery; macrophage‐membrane‐coated nanoparticles: nanoparticles coated with macrophage membranes leverage macrophage tropism for TME‐targeted delivery, enhancing therapeutic specificity and efficacy.

scRNA‐seq has revolutionized the investigation of cellular heterogeneity by enabling comprehensive gene expression profiling at single‐cell resolution [457]. With significant advancements in experimental protocols and bioinformatics pipelines in recent years, scRNA‐seq has been extensively employed to investigate cellular heterogeneity across virtually all cancer types [458, 459]. This approach provides unprecedented insights into the multifaceted dynamics of malignant cells and their TME, including intricate cell–cell communication networks between neoplastic cells and diverse stromal populations. Beyond cataloging cellular diversity, scRNA‐seq deciphers molecular drivers of oncogenic transformation and malignant evolution while systematically cataloging the functional and transcriptional heterogeneity of distinct cellular subpopulations across spatially and temporally evolving TME landscapes [275] (Figure 7A). This approach is increasingly utilized to categorize macrophage heterogeneity in malignant tissues and precisely delineate TAMs subpopulations [276]. For instance, in CRC, scRNA‐seq analyses have uncovered two separate TAMs subpopulations: C1QC^+^ macrophages and SPP1^+^ macrophages. Evidence indicates that the targeted elimination of SPP1^+^ TAMs may improve the effectiveness of myeloid‐oriented immunotherapies [277]. In summary, this technological breakthrough provides unprecedented tools for deconvoluting tumor heterogeneity and holds significant promise for advancing cancer diagnosis and therapeutic development [460].

### 5.2 TAMs‐Based Biologics

RG7155, the pioneering TAMs‐targeted antibody advanced to clinical trials, functions as a CSF1R inhibitor that disrupts receptor homodimerization and subsequent signal transduction initiated by either CSF1 or its more recently characterized ligand IL‐34 [461]. This foundational investigation demonstrated that CSF1R antibodies could selectively deplete TAMs while promoting M2‐to‐M1 phenotype polarization. However, the monotherapy demonstrated restricted antitumor activity in solid tumors, while tumors exhibited a propensity to acquire treatment resistance (Figure 7B). To overcome these limitations, CSF1R antibodies are now being clinically evaluated in combination with PD‐1 inhibitors monoclonal antibodies. Studies by Zhu et al. have shown that blocking the CSF1/CSF1R signaling pathway in pancreatic tumors can eliminate CD206^+^ TAMs and reprogram the residual macrophages toward antitumor immunity [462]. While solitary CSF1/CSF1R inhibition moderately enhances the antitumor interferon response, promote the infiltration of CTLs, and suppress tumor progression. However, the therapeutic efficacy is limited by compensatory upregulation of immune checkpoint pathways, notably PD‐L1 expression on malignant cells and CTLA‐4 induction in T cells. The combination of CSF1/CSF1R blockade with PD‐1 and CTLA‐4 antagonists can significantly enhance the efficacy of immunotherapy and can induce volumetric reduction or regression of established pancreatic cancers. Bispecific antibody fusion proteins have been successfully developed to potently augment macrophage phagocytic activity against tumor cells. Yun Yang et al. targeted the CD47/SIRPα and CD24/Siglec‐10 signaling pathways by developing a bispecific antibody fusion protein, PPAB001, directed against both CD47 and CD24. In murine 4T1 syngeneic tumor models and human SK‐OV‐3 xenograft tumor models, PPAB001 demonstrated superior tumor growth inhibition compared with single‐targeting CD47 or CD24 therapies, while maintaining advantageous tolerability profiles with diminished hematological toxicity risks [463].

### 5.3 Clinical Trials of CAR‐M Therapy

#### 5.3.1 Clinical Responses and Preliminary Efficacy

CAR‐T cell therapies have revolutionized the standard treatment for hematological malignancies while confronting persistent challenges in solid tumor applications [464, 465]. Critical limitations including heterogeneous antigen expression, impaired tumor infiltration [466], poor proliferation and persistence [467], combined with the profoundly immunosuppressive TME, collectively limit CAR‐T cell efficacy [468, 469, 470]. As an alternative, CAR‐M present distinct advantages: they exert antitumor effects through direct phagocytosis, proinflammatory cytokine release, TME remodeling, and antigen presentation, thereby coordinately activating innate and adaptive immune responses. As of November 2020, two clinical trials based on the CAR‐M therapy have been approved by the United States Food and Drug Administration. The first one is CT‐0508, a drug candidate developed by CARISMA Therapeutics. Reiss reported interim results from a first‐in‐human Phase I study evaluated CT‐0508, a CAR‐M product targeting EGFR2 (HER2), in participants with malignancies exhibiting HER2 overexpression. This investigation enrolled 14 participants, predominantly with breast and gastroesophageal carcinomas, who received CT‐0508 administration without preceding lymphodepletion conditioning [471]. The study's primary endpoints focused on safety and manufacturability, while secondary endpoints evaluated cellular kinetics and antitumor efficacy. Serial biopsies confirmed that CT‐0508 successfully trafficked to tumor sites, reprogrammed the local microenvironment, and promoted significant CD8^+^T cell expansion. These early findings demonstrate the safety, feasibility, and tumor‐targeting potential of CAR‐M therapy, laying a foundation for the continued refinement and clinical development of myeloid‐based immunotherapies [471]. This first‐in‐human trial of CT‐0508 establishes a clinical proof‐of‐concept for CAR‐M therapy in HER2^+^ solid tumors. While limitations remain, the study demonstrates that macrophage‐based therapies can be safely administered, remodel the TME, and engage adaptive immunity. CAR‐Ms represent a promising new frontier in cancer immunotherapy, offering a unique mechanism of action that may complement existing T‐cell‐based approaches [344, 472].

The second is MaxCyte's MCY‐M11, which utilizes mRNA‐targeted PBMCs to express mesothelin‐CAR. It is intended for patients with relapsed or refractory ovarian cancer and peritoneal mesothelioma, and recruitment of volunteers for its Phase I clinical trial has been initiated [473].

#### 5.3.2 Clinical Challenges and Limitations

CAR‐M therapy has demonstrated considerable potential in preliminary investigations; however, its translational trajectory encounters several formidable challenges. A fundamental limitation concerns the abbreviated in vivo persistence of CAR‐M cells, particularly when utilizing transient mRNA delivery platforms, which may undermine sustained antitumor responses. Numerous strategies are under exploration to augment macrophage longevity and functional capacity postadministration, including implementation of autocrine cytokine signaling, introduction of genetic modifications to confer apoptosis resistance, and incorporation of memory‐like properties.

Effective targeting and penetration constitute another significant barrier. Although macrophages possess innate traffic toward inflammatory sites, immunosuppressive elements within the TME, such as IL‐10, TGF‐β, and hypoxic conditions, can disrupt migratory patterns or induce polarization toward tumor‐promoting M2‐like phenotypes. Contemporary research emphasizes refinement of delivery modalities (including intratumoral or intraperitoneal injection), augmentation of chemokine receptor signaling pathways, and development of protective mechanisms against inhibitory TME signals.

The intrinsic plasticity of macrophages, while advantageous for cellular reprogramming, introduces substantial clinical risks. Under TME pressure, CAR‐M cells may undergo phenotypic conversion to immunosuppressive, protumorigenic M2‐like states. Maintenance of proinflammatory M1 polarization presents particular difficulties in strongly immunosuppressive solid malignancies. This challenge has catalyzed the development of “armored” CAR‐M constructs capable of secreting inflammatory cytokines or expressing receptors that neutralize inhibitory signaling.

Substantial manufacturing and logistical obstacles persist. Most current CAR‐M paradigms rely on patient‐specific, fresh cell products with constrained production timelines, complicating broad implementation. The creation of universal, off‐the‐shelf CAR‐M platforms derived from iPSCs or immortalized cell lines could improve production scalability, though these technologies remain embryonic. Furthermore, regulatory frameworks for macrophage‐based therapeutics are less defined compared with CAR‐T products, adding another layer of complexity to clinical advancement.

Persistent safety considerations warrant careful attention. The broad tissue distribution of many solid tumor antigens necessitates rigorous assessment of on‐target, off‐tumor toxicities. Investigational mitigation strategies include refined targeting systems, dual‐antigen recognition mechanisms, and integrated safety controls such as molecular logic gates or elimination switches. Ultimately, the absence of validated predictive biomarkers for patient selection and inadequate immunocompetent preclinical models hinder both trial design and therapeutic optimization. Addressing these multifactorial challenges is imperative for realizing the complete clinical potential of CAR‐M intervention.

### 5.4 Macrophage‐Based Drug Carriers for Drug Delivery

Macrophages have gained significant attention as promising cellular carriers for targeted delivery of therapeutic agents and nanoparticles to the TME. Their natural biological properties, including biocompatibility, low immunogenicity, prolonged circulation time, ability to cross biological barriers, and capacity to migrate and accumulate at inflammatory sites such as tumors, make them well‐suited for this role. Macrophages, along with their membranes and secreted exosomes, represent an emerging platform for antitumor drug delivery, offering versatile and biomimetic strategies to enhance therapeutic precision and efficacy [474] (Figure 7C). For drugs encapsulated within macrophages, these properties enable active accumulation in tumors independent of the enhanced permeability and retention (EPR) effect, while reducing cytotoxicity and enhancing therapeutic efficacy through controlled release and extended circulation half‐life. A particularly compelling application involves cerebral drug delivery to intracranial neoplasms [475]. The selective permeability of the BBB restricts the translocation of pharmacological agents, significantly limiting drug delivery to intracranial tumors. During tumorigenesis, the BBB undergoes structural disruption mediated by the EPR effect, leading to enhanced vascular leakage and disrupts lymphatic drainage. However, elevated interstitial fluid pressure and the blood–tumor barrier still restrict therapeutic drug penetration into the tumor. Successful drug delivery to intracranial lesions therefore necessitates active transport mechanisms capable of traversing these biological barriers. The inflammatory response in tumors induces the overexpression of cell adhesion molecules on endothelial cells, facilitating interactions between macrophages and endothelial cells. This promotes the initial rolling, firm adhesion, and migration of macrophages, allowing them to cross the BBB and infiltrate the cerebral parenchyma. As the dominant immune constituents of glioblastomas, macrophages represent optimal biological vectors for nanoparticle delivery to the brain TME.

The principal cellular sources for macrophage drug carriers include BMDMs and the RAW264.7 macrophages. These cells exhibit inherent tumor‐targeting capabilities mediated by α4 and β1 integrins, which facilitate binding to VCAM‐1 expressed on malignant cells [476]. Previous investigations have successfully loaded doxorubicin (DOX) into RAW264.7 macrophages, establishing a biomimetic drug delivery system. This system demonstrated specific targeting toward 4T1 metastatic mammary carcinoma cells without compromising their tumorigenic properties, while significantly prolonging survival in murine models [477, 478].

The macrophage membrane surface abounds with lipids and proteins, thus presenting an opportunity for loading nanodrugs through chemical conjugation or physical adsorption [437]. This approach not only prolongs systemic circulation but also promotes intratumoral penetration, thereby enhancing therapeutic efficacy [436, 437]. Parodi et al. [438] advanced this concept by coating mesoporous silica nanoparticles with macrophage membranes, yielding macrophage‐coated nanoparticles (MCNPs) that evade immune clearance. These MCNPs selectively recognize inflammatory receptors on tumor vasculature through surface markers such as CD11a, promoting targeted accumulation within tumor tissues.

Exosomes derived from macrophages present another promising vehicle for targeted therapy, retaining favorable parental membrane characteristics that support cellular uptake and membrane fusion [478, 479]. To overcome therapeutic challenges in glioblastoma, including the BBB and tumor hypoxia, Wu et al. engineered exosome‐coated silica nanoparticles encapsulating catalase (CSI@Ex‐A) [480]. This construct demonstrated efficient BBB traversal and precise tumor targeting. Upon endocytosis, catalase release catalyzes hydrogen peroxide decomposition, generating oxygen and ameliorating hypoxic conditions [480]. Collectively, these findings corroborate the advantages of exosomal drug delivery, including superior biocompatibility, prolonged circulation half‐life, enhanced drug loading efficiency, and improved safety profiles [481].

Despite these advantages, using macrophages as carriers presents several limitations that pose challenges to their further development and clinical translation. A fundamental concern is the potential functional impairment caused by internalized therapeutic agents, which may disrupt essential cellular processes. The particles should not affect the migration capacity of macrophages or their ability to effectively deliver and release drugs at the target site. Another challenge is the lack of control over loading capacity, as macrophages can only internalize a limited number of particles. Furthermore, the stability of macrophages loaded with fragile or biodegradable drugs remains unclear, as these drugs may undergo intracellular degradation or inactivation, leading to premature release. These limitations present substantial translational challenges. However, with rapid advancements in cell‐based drug delivery research and the increasing number of clinical trials employing other cell types (e.g., red blood cells, T cells, NK cells, and stem cells), macrophages are expected to enter clinical applications within the next few years.

## 6 Conclusion and Future Perspectives

TAMs represent the predominant immune cell population within the TME, exhibiting profound functional and phenotypic heterogeneity while serving as pivotal regulators of tumor progression, metastasis, and response to immunotherapy. Given their plasticity and dual capacity to either promote tumor growth or orchestrate antitumor immunity, deciphering the precise mechanisms that control TAMs polarization and identifying macrophage‐specific therapeutic targets remain central to improving current and emerging immunotherapies. Early therapeutic efforts focused largely on depleting TAMs, but these approaches faced significant limitations, including disruption of physiological homeostasis through nonselective elimination of TRMs and potential induction of prometastatic sequelae following macrophage ablation. Such limitations underscore the need for more refined strategies that can suppress tumor‐promoting macrophage functions while preserving their physiological roles. Consequently, reprogramming TAMs from a protumor (M2‐like) to an antitumor (M1‐like) phenotype has emerged as a promising alternative. This approach may synergize with existing treatments such as ICIs, CAR macrophage (CAR‐M) therapies, and macrophage‐mediated drug delivery platforms, potentially reinvigorating antitumor immune responses. However, the clinical validity of such combination or reprogramming‐based regimens requires rigorous validation in well‐designed, large‐scale trials. Concurrently, nanoparticle‐based approaches for precision modulation of TAMs function have shown encouraging results in preclinical models, offering innovative means to selectively deliver immunomodulatory agents. However, the translation of these nanomedicines into clinical practice demands careful evaluation of their efficiency, safety, and long‐term tolerability in humans.

Despite these advances, several critical questions remain unresolved. While signaling pathways driving TAMs polarization are increasingly understood, the metabolic reprogramming of TAMs during malignant progression and its contribution to therapy resistance remains incompletely characterized. This gap in knowledge represents an important frontier for future research, especially given the known interplay between metabolic and immune signaling networks. Furthermore, emerging immune checkpoints such as T‐cell Ig and ITIM domain, V‐domain Ig suppressor of T‐cell activation, and lymphocyte activation gene‐3 have attracted significant attention as next‐generation immunotherapeutic targets. Integrating these checkpoints into TAMs‐targeted therapeutic strategies could help overcome resistance to existing ICIs, but the complex regulatory networks controlling these molecules require further clarification to enable precise and effective targeting. Furthermore, the integration of fundamental macrophage biology, advanced technologies such as single‐cell analytics, and translational immunotherapy research will accelerate the development of next‐generation, macrophage‐centric cancer therapies. By strategically harnessing the immunostimulatory potential of TAMs and incorporating them into multimodal treatment regimens, the field moves closer to delivering more durable, effective, and personalized cancer immunotherapies.

## Author Contributions

Wurihan Bao and Xiaojie Qu: writing original – draft and data curation. Yiqi Wang: investigation. Dan Huang: software. Huiling Zhang, Mingyuan Dong, and Han Sun: formal analysis. Zhaogang Yang and Xuefeng Li: review and editing, project administration, resources, funding acquisition, and supervision. All authors have read and approved the final paper.

## Funding

This work is supported by National Natural Science Foundation of China (82422041, X.L., 21HAA01203, Z.Y.) and Jilin Science‐Technology Committee (20230402042GH, Z.Y.).

## Conflicts of Interest

The authors declare no conflicts of interest.

## Ethics Statement

This study did not involve human participants and/or animals or informed consent. Thus, ethical clearance is not applicable to this article.

## Data Availability

No data were used for the research described in the article.

## References

[1] W. H. Fridman , L. Zitvogel , C. Sautès‐Fridman , et al., “The Immune Contexture in Cancer Prognosis and Treatment,” Nature Reviews Clinical Oncology 14, no. 12 (2017): 717–734. 10.1038/nrclinonc.2017.101 28741618

[2] D. Bruni , K. Angell Helen , and J. Galon , “The Immune Contexture and Immunoscore in Cancer Prognosis and Therapeutic Efficacy,” Nature Reviews Cancer 20, no. 11 (2020): 662–680. 32753728 10.1038/s41568-020-0285-7

[3] N. Cox , M. Pokrovskii , R. Vicario , et al., “Origins, Biology, and Diseases of Tissue Macrophages,” Annual Review of Immunology 39, no. 1 (2021): 313–344. 10.1146/annurev-immunol-093019-111748 PMC10786183 33902313

[4] S.‐Y. Wu and K. Watabe , “The Roles of Microglia/Macrophages in Tumor Progression of Brain Cancer and Metastatic Disease,” Frontiers in Bioscience (Landmark Edition) 22 (2017): 1805. 28410147 10.2741/4573 PMC5658785

[5] A. Christofides , L. Strauss , A. Yeo , et al., “The Complex Role of Tumor‐Infiltrating Macrophages,” Nature Immunology 23, no. 8 (2022): 1148–1156. 35879449 10.1038/s41590-022-01267-2 PMC10754321

[6] G. Teti , C. Biondo , and C. Beninati , “The Phagocyte, Metchnikoff, and the Foundation of Immunology,” Microbiology Spectrum 4, no. 2 (2016): 10. 10.1128/microbiolspec.MCHD-0009-2015 27227301

[7] M. T. Silva and M. Correia‐Neves , “Neutrophils and Macrophages: The Main Partners of Phagocyte Cell Systems,” Frontiers in Immunology 3 (2012): 174. 22783254 10.3389/fimmu.2012.00174 PMC3389340

[8] S. Yona and S. Gordon , “From the Reticuloendothelial to Mononuclear Phagocyte System—the Unaccounted Years,” Frontiers in Immunology 6 (2015): 328. 26191061 10.3389/fimmu.2015.00328 PMC4486871

[9] R. V. Furth , Z. A. Cohn , J. G. Hirsch , et al., “The Mononuclear Phagocyte System: A New Classification of Macrophages, Monocytes, and Their Precursor Cells,” Bulletin of the World Health Organization 46, no. 6 (1972): 845–852. 4538544 PMC2480884

[10] T. Lazarov , S. Juarez‐Carreño , N. Cox , et al., “Physiology and Diseases of Tissue‐Resident Macrophages,” Nature 618, no. 7966 (2023): 698–707. 37344646 10.1038/s41586-023-06002-x PMC10649266

[11] E. G. Perdiguero and F. Geissmann , “The Development and Maintenance of Resident Macrophages,” Nature Immunology 17, no. 1 (2016): 2–8. 26681456 10.1038/ni.3341 PMC4950995

[12] E. Mass , I. Ballesteros , M. Farlik , et al., “Specification of Tissue‐Resident Macrophages during Organogenesis,” Science 353 (2016): 6304. 10.1126/science.aaf4238 PMC5066309 27492475

[13] A. H. David , M. I. Katharine , and C. Pridans , “The Mononuclear Phagocyte System: The Relationship Between Monocytes and Macrophages,” Trends in Immunology 40, no. 2 (2019): 98–112. 30579704 10.1016/j.it.2018.11.007

[14] P. E. Gomez , K. Kay , and S. Christian , “The Origin of Tissue‐Resident Macrophages: When an Erythro‐Myeloid Progenitor Is an Erythro‐Myeloid Progenitor,” Immunity 43 (2015): 1023–1024. 26682973 10.1016/j.immuni.2015.11.022

[15] L. van de Laar , W. Saelens , S. De Prijck , et al., “Yolk Sac Macrophages, Fetal Liver, and Adult Monocytes Can Colonize an Empty Niche and Develop Into Functional Tissue‐Resident Macrophages,” Immunity 44, no. 4 (2016): 755–768. 26992565 10.1016/j.immuni.2016.02.017

[16] F. Ginhoux and M. Guilliams , “Tissue‐Resident Macrophage Ontogeny and Homeostasis,” Immunity 44, no. 3 (2016): 439–449. 26982352 10.1016/j.immuni.2016.02.024

[17] S. De Schepper , S. Verheijden , J. Aguilera‐Lizarraga , et al., “Self‐Maintaining Gut Macrophages Are Essential for Intestinal Homeostasis,” Cell 175, no. 2 (2018): 400–415. e13. 30173915 10.1016/j.cell.2018.07.048

[18] Q. Yang , N. Guo , Y. Zhou , et al., “The Role of Tumor‐Associated Macrophages (Tams) in Tumor Progression and Relevant Advance in Targeted Therapy,” Acta Pharm Sin B 10, no. 11 (2020): 2156–2170. 33304783 10.1016/j.apsb.2020.04.004 PMC7714989

[19] A. Mantovani , A. Sica , and M. Locati , “Macrophage Polarization Comes of Age,” Immunity 23, no. 4 (2005): 344–346. 16226499 10.1016/j.immuni.2005.10.001

[20] B. Bottazzi , E. Erba , N. Nobili , et al., “A Paracrine Circuit in the Regulation of the Proliferation of Macrophages Infiltrating Murine Sarcomas,” The Journal of Immunology 144, no. 6 (1990): 2409–2412. 2138198

[21] B.‐Z. Qian and W. Pollard Jeffrey , “Macrophage Diversity Enhances Tumor Progression and Metastasis,” Cell 141, no. 1 (2010): 39–51. 20371344 10.1016/j.cell.2010.03.014 PMC4994190

[22] K. Movahedi , D. Laoui , C. Gysemans , et al., “Different Tumor Microenvironments Contain Functionally Distinct Subsets of Macrophages Derived From Ly6c (High) Monocytes,” Cancer Research 70, no. 14 (2010): 5728–5739. 20570887 10.1158/0008-5472.CAN-09-4672

[23] J. Pittet Mikael , O. Michielin , and D. Migliorini , “Clinical Relevance of Tumour‐Associated Macrophages,” Nature reviews Clinical oncology 19, no. 6 (2022): 402–421. 10.1038/s41571-022-00620-6 35354979

[24] A. H. Klimp , E. G. E. De Vries , G. L. Scherphof , et al., “A Potential Role of Macrophage Activation in the Treatment of Cancer,” Critical reviews in oncology/hematology 44, no. 2 (2002): 143–161. 12413632 10.1016/s1040-8428(01)00203-7

[25] Y. Pan , Y. Yu , X. Wang , et al., “Tumor‐Associated Macrophages in Tumor Immunity,” Frontiers in Immunology 11 (2020): 583084. 33365025 10.3389/fimmu.2020.583084 PMC7751482

[26] J. Garbán Hermes and B. Bonavida , “Nitric Oxide Sensitizes Ovarian Tumor Cells to Fas‐Induced Apoptosis,” Gynecologic Oncology 73, no. 2 (1999): 257–264. 10329044 10.1006/gyno.1999.5374

[27] D. Duluc , M. Corvaisier , S. Blanchard , et al., “Interferon‐Γ Reverses the Immunosuppressive and Protumoral Properties and Prevents the Generation of Human Tumor‐Associated Macrophages,” International Journal of Cancer 125, no. 2 (2009): 367–373. 19378341 10.1002/ijc.24401

[28] S. Chen , W. T. Lai Seigmund , E. Brown Christine , et al., “Harnessing and Enhancing Macrophage Phagocytosis for Cancer Therapy,” Frontiers in Immunology 12 (2021): 635173. 33790906 10.3389/fimmu.2021.635173 PMC8006289

[29] H. Bruns , M. Büttner , M. Fabri , et al., “Vitamin D–Dependent Induction of Cathelicidin in Human Macrophages Results in Cytotoxicity Against High‐Grade B Cell Lymphoma,” Science Translational Medicine 7, no. 282 (2015): 282ra47. 10.1126/scitranslmed.aaa3230 25855493

[30] S. Das , E. Sarrou , S. Podgrabinska , et al., “Tumor Cell Entry Into the Lymph Node Is Controlled by Ccl1 Chemokine Expressed by Lymph Node Lymphatic Sinuses,” Journal of Experimental Medicine 210, no. 8 (2013): 1509–1528. 23878309 10.1084/jem.20111627 PMC3727324

[31] P.‐F. Ma , C.‐C. Gao , J. Yi , et al., “Cytotherapy With M1‐Polarized Macrophages Ameliorates Liver Fibrosis by Modulating Immune Microenvironment in Mice,” Journal of Hepatology 67, no. 4 (2017): 770–779. 28596109 10.1016/j.jhep.2017.05.022

[32] E. Cendrowicz , Z. Sas , E. Bremer , et al., “The Role of Macrophages in Cancer Development and Therapy,” Cancers 13, no. 08 (2021): 1946. 33919517 10.3390/cancers13081946 PMC8073377

[33] C. E. Lewis and J. W. Pollard , “Distinct Role of Macrophages in Different Tumor Microenvironments,” Cancer Research 66, no. 2 (2006): 605–612. 16423985 10.1158/0008-5472.CAN-05-4005

[34] T. Mitsudomi and Y. Yatabe , “Epidermal Growth Factor Receptor in Relation to Tumor Development: Egfr Gene and Cancer,” Febs Journal 277, no. 2 (2010): 301–308. 19922469 10.1111/j.1742-4658.2009.07448.x

[35] L. K. Huynh , C. J. Hipolito , and P. Ten Dijke , “A Perspective on the Development of Tgf‐Β Inhibitors for Cancer Treatment,” Biomolecules 9, no. 11 (2019), 10.3390/biom9110743. PMC6921009 31744193

[36] C. Liu , W. Zhang , J. Wang , et al., “Tumor‐Associated Macrophage‐Derived Transforming Growth Factor‐Β Promotes Colorectal Cancer Progression Through Hif1‐Trib3 Signaling,” Cancer Science 112, no. 10 (2021): 4198–4207. 34375482 10.1111/cas.15101 PMC8486199

[37] C. Guruvayoorappan , “Tumor versus Tumor‐Associated Macrophages: How Hot Is the Link?,” Integrative Cancer Therapies 7, no. 2 (2008): 90–95. 18550889 10.1177/1534735408319060

[38] P. Chen , Y. Huang , R. Bong , et al., “Tumor‐Associated Macrophages Promote Angiogenesis and Melanoma Growth via Adrenomedullin in a Paracrine and Autocrine Manner,” Clinical Cancer Research 17, no. 23 (2011): 7230–7239. 21994414 10.1158/1078-0432.CCR-11-1354

[39] A. Castro Brandyn , P. Flanigan , A. Jahangiri , et al., “Macrophage Migration Inhibitory Factor Downregulation: A Novel Mechanism of Resistance to Anti‐Angiogenic Therapy,” Oncogene 36, no. 26 (2017): 3749–3759. 28218903 10.1038/onc.2017.1 PMC5491354

[40] G. Zhang , X. Tao , B. Ji , et al., “Hypoxia‐Driven M2‐Polarized Macrophages Facilitate Cancer Aggressiveness and Temozolomide Resistance in Glioblastoma,” Oxid Med Cell Longev 2022 (2022): 1614336. 36046687 10.1155/2022/1614336 PMC9423979

[41] Y. Komohara , Y. Fujiwara , K. Ohnishi , et al., “Tumor‐Associated Macrophages: Potential Therapeutic Targets for Anti‐Cancer Therapy,” Advanced drug delivery reviews 99 (2016): 180–185. 26621196 10.1016/j.addr.2015.11.009

[42] K. Movahedi and J. O. A. Van Ginderachter , “The Ontogeny and Microenvironmental Regulation of Tumor‐Associated Macrophages,” Antioxidants & Redox Signaling 25, no. 14 (2016): 775–791. 27020982 10.1089/ars.2016.6704

[43] W. Sun , F. Q. Wei , W. J. Li , et al., “A Positive‐Feedback Loop Between Tumour Infiltrating Activated Treg Cells and Type 2‐Skewed Macrophages Is Essential for Progression of Laryngeal Squamous Cell Carcinoma,” British Journal of Cancer 117, no. 11 (2017): 1631–1643. 28949956 10.1038/bjc.2017.329 PMC5729431

[44] S. R. Gordon , R. L. Maute , B. W. Dulken , et al., “Pd‐1 Expression by Tumour‐Associated Macrophages Inhibits Phagocytosis and Tumour Immunity,” Nature 545, no. 7655 (2017): 495–499. 28514441 10.1038/nature22396 PMC5931375

[45] W. Li , F. Wu , S. Zhao , et al., “Correlation Between Pd‐1/Pd‐L1 Expression and Polarization in Tumor‐Associated Macrophages: A Key Player in Tumor Immunotherapy,” Cytokine & Growth Factor Reviews 67 (2022): 49–57. 35871139 10.1016/j.cytogfr.2022.07.004

[46] Y. Suhail , M. P. Cain , K. Vanaja , et al., “Systems Biology of Cancer Metastasis,” Cell Systems 9, no. 2 (2019): 109–127. 31465728 10.1016/j.cels.2019.07.003 PMC6716621

[47] S. Gerstberger , Q. Jiang , and K. Ganesh , “Metastasis,” Cell 186, no. 8 (2023): 1564–1579. 37059065 10.1016/j.cell.2023.03.003 PMC10511214

[48] D. Hanahan and R. A. Weinberg , “Hallmarks of Cancer: The Next Generation,” Cell 144, no. 5 (2011): 646–674. 21376230 10.1016/j.cell.2011.02.013

[49] S. Valastyan and R. A. Weinberg , “Tumor Metastasis: Molecular Insights and Evolving Paradigms,” Cell 147, no. 2 (2011): 275–292. 22000009 10.1016/j.cell.2011.09.024 PMC3261217

[50] T. Baslan and J. Hicks , “Unravelling Biology and Shifting Paradigms in Cancer With Single‐Cell Sequencing,” Nature Reviews Cancer 17, no. 9 (2017): 557–569. 28835719 10.1038/nrc.2017.58

[51] S. M. Morrissey , F. Zhang , C. Ding , et al., “Tumor‐Derived Exosomes Drive Immunosuppressive Macrophages in a Pre‐Metastatic Niche Through Glycolytic Dominant Metabolic Reprogramming,” Cell Metabolism 33, no. 10 (2021): 2040–2058. e10. 34559989 10.1016/j.cmet.2021.09.002 PMC8506837

[52] X. W. Chen , T. J. Yu , J. Zhang , et al., “Cyp4a in Tumor‐Associated Macrophages Promotes Pre‐Metastatic Niche Formation and Metastasis,” Oncogene 36, no. 35 (2017): 5045–5057. 28481877 10.1038/onc.2017.118 PMC5582214

[53] M. Qiu , K. Huang , Y. Liu , et al., “Modulation of Intestinal Microbiota by Glycyrrhizic Acid Prevents High‐Fat Diet‐Enhanced Pre‐Metastatic Niche Formation and Metastasis,” Mucosal Immunology 12, no. 4 (2019): 945–957. 30755716 10.1038/s41385-019-0144-6

[54] H. Kim , H. Chung , J. Kim , et al., “Macrophages‐Triggered Sequential Remodeling of Endothelium‐Interstitial Matrix to Form Pre‐Metastatic Niche in Microfluidic Tumor Microenvironment,” Adv Sci (Weinh) 6, no. 11 (2019): 1900195. 31179226 10.1002/advs.201900195 PMC6548952

[55] M. Umakoshi , S. Takahashi , G. Itoh , et al., “Macrophage‐Mediated Transfer of Cancer‐Derived Components to Stromal Cells Contributes to Establishment of a Pro‐Tumor Microenvironment,” Oncogene 38, no. 12 (2019): 2162–2176. 30459356 10.1038/s41388-018-0564-x

[56] E. Nasrollahzadeh , S. Razi , M. Keshavarz‐Fathi , et al., “Pro‐Tumorigenic Functions of Macrophages at the Primary, Invasive and Metastatic Tumor Site,” Cancer Immunology, Immunotherapy 69, no. 9 (2020): 1673–1697. 32500231 10.1007/s00262-020-02616-6 PMC11027658

[57] Y. Liu and X. Cao , “Characteristics and Significance of the Pre‐Metastatic Niche,” Cancer Cell 30, no. 5 (2016): 668–681. 27846389 10.1016/j.ccell.2016.09.011

[58] M. Wang , Z. Qin , J. Wan , et al., “Tumor‐Derived Exosomes Drive Pre‐Metastatic Niche Formation in Lung via Modulating Ccl1(+) Fibroblast and Ccr8(+) Treg Cell Interactions,” Cancer Immunology, Immunotherapy 71, no. 11 (2022): 2717–2730. 35428909 10.1007/s00262-022-03196-3 PMC10992578

[59] S. Liu , M. Jiang , Q. Zhao , et al., “Vascular Endothelial Growth Factor Plays a Critical Role in the Formation of the Pre‐Metastatic Niche via Prostaglandin E2,” Oncology Reports 32, no. 6 (2014): 2477–2484. 25333935 10.3892/or.2014.3516

[60] Y. Wang , Y. Li , J. Zhong , et al., “Tumor‐Derived Cav‐1 Promotes Pre‐Metastatic Niche Formation and Lung Metastasis in Breast Cancer,” Theranostics 13, no. 5 (2023): 1684–1697. 37056561 10.7150/thno.79250 PMC10086203

[61] V. Catalano , A. Turdo , S. Di Franco , et al., “Tumor and Its Microenvironment: A Synergistic Interplay,” Seminars in Cancer Biology 23, no. 6 (2013): 522–532. Pt B. 24012661 10.1016/j.semcancer.2013.08.007

[62] M. Werner‐Klein , A. Grujovic , C. Irlbeck , et al., “Interleukin‐6 Trans‐Signaling Is a Candidate Mechanism to Drive Progression of Human Dccs during Clinical Latency,” Nature Communications 11, no. 1 (2020): 4977. 10.1038/s41467-020-18701-4 PMC7536220 33020483

[63] T. Beltraminelli and M. De Palma , “Biology and Therapeutic Targeting of Tumour‐Associated Macrophages,” The Journal of Pathology 250, no. 5 (2020): 573–592. 32086811 10.1002/path.5403

[64] G. Denardo David and B. Ruffell , “Macrophages as Regulators of Tumour Immunity and Immunotherapy,” Nature Reviews Immunology 19, no. 6 (2019): 369–382. 10.1038/s41577-019-0127-6 PMC7339861 30718830

[65] A. Mantovani , F. Marchesi , A. Malesci , et al., “Tumour‐Associated Macrophages as Treatment Targets in Oncology,” Nature Reviews Clinical Oncology 14, no. 7 (2017): 399–416. 10.1038/nrclinonc.2016.217 PMC5480600 28117416

[66] S. K. Biswas , “Metabolic Reprogramming of Immune Cells in Cancer Progression,” Immunity 43, no. 3 (2015): 435–449. 26377897 10.1016/j.immuni.2015.09.001

[67] J. Lin , Y. Chen , Z. Zhang , et al., “Calreticulin, a Potential Coregulator of Immune Checkpoints and Biomarker Associated With Tumor Microenvironment and Clinical Prognostic Significance in Breast Invasive Carcinoma,” Environmental Toxicology 39, no. 5 (2024): 2717–2731. 38247288 10.1002/tox.24120

[68] L. Xiao , L. Zhang , C. Guo , et al., “Find Me and Eat Me Signals: Tools to Drive Phagocytic Processes for Modulating Antitumor Immunity,” Cancer Communications 44, no. 7 (2024): 791–832. 38923737 10.1002/cac2.12579 PMC11260773

[69] Galvez‐Cancino Felipe , P. Simpson Alexander , C. Costoya , et al., “Fcγ Receptors and Immunomodulatory Antibodies in Cancer,” Nature Reviews Cancer 24, no. 1 (2024): 51–71. 38062252 10.1038/s41568-023-00637-8

[70] I.‐M. Kur and A. Weigert , “Phosphatidylserine Externalization as Immune Checkpoint in Cancer,” Pflügers Archiv‐European Journal of Physiology 476, no. 12 (2024): 1789–1802. 38573347 10.1007/s00424-024-02948-7 PMC11582130

[71] M. Feng , W. Jiang , B. Y. S. Kim , et al., “Phagocytosis Checkpoints as New Targets for Cancer Immunotherapy,” Nature Reviews Cancer 19, no. 10 (2019): 568–586. 31462760 10.1038/s41568-019-0183-z PMC7002027

[72] W. H. Fridman , L. Zitvogel , C. Sautès‐Fridman , et al., “The Immune Contexture in Cancer Prognosis and Treatment,” Nature reviews Clinical oncology 14, no. 12 (2017): 717–734. 10.1038/nrclinonc.2017.101 28741618

[73] J. Fucikova , R. Spisek , G. Kroemer , et al., “Calreticulin and Cancer,” Cell Research 31 (2021): 5–16. 32733014 10.1038/s41422-020-0383-9 PMC7853084

[74] S. Basu , J. Binder Robert , T. Ramalingam , et al., “Cd91 Is a Common Receptor for Heat Shock Proteins Gp96, Hsp90, Hsp70, and Calreticulin,” Immunity 14, no. 3 (2001): 303–313. 11290339 10.1016/s1074-7613(01)00111-x

[75] A. Ogden Carol , A. Decathelineau , R. Hoffmann Peter , et al., “C1q and Mannose Binding Lectin Engagement of Cell Surface Calreticulin and Cd91 Initiates Macropinocytosis and Uptake of Apoptotic Cells,” The Journal of Experimental Medicine 194, no. 6 (2001): 781–796. 11560994 10.1084/jem.194.6.781 PMC2195958

[76] R. W. Vandivier , A. Ogden Carol , A. Fadok Valerie , et al., “Role of Surfactant Proteins a, D, and C1q in the Clearance of Apoptotic Cells in Vivo and in Vitro: Calreticulin and Cd91 as a Common Collectin Receptor Complex,” The Journal of Immunology 169, no. 7 (2002): 3978–3986. 12244199 10.4049/jimmunol.169.7.3978

[77] W. Pang Wendy , V. Pluvinage John , A. Price Elizabeth , et al., “Hematopoietic Stem Cell and Progenitor Cell Mechanisms in Myelodysplastic Syndromes,” Proceedings of the National Academy of Sciences 110, no. 8 (2013): 3011–3016. 10.1073/pnas.1222861110 PMC3581956 23388639

[78] P. Chao Mark , S. Jaiswal , R. Weissman‐Tsukamoto , et al., “Calreticulin Is the Dominant Pro‐Phagocytic Signal on Multiple Human Cancers and Is Counterbalanced by Cd47,” Science Translational Medicine 2, no. 63 (2010): 63ra94–63ra94. 10.1126/scitranslmed.3001375 PMC4126904 21178137

[79] I. Shachar , A. Barak , H. Lewinsky , et al., “Slamf Receptors on Normal and Malignant B Cells,” Clinical Immunology 204 (2019): 23–30. 30448442 10.1016/j.clim.2018.10.020

[80] L. Gautier Emmanuel , T. Shay , J. Miller , et al., “Gene‐Expression Profiles and Transcriptional Regulatory Pathways That Underlie the Identity and Diversity of Mouse Tissue Macrophages,” Nature Immunology 13, no. 11 (2012): 1118–1128. 23023392 10.1038/ni.2419 PMC3558276

[81] C. Miller Jennifer , D. Brown Brian , T. Shay , et al., “Deciphering the Transcriptional Network of the Dendritic Cell Lineage,” Nature Immunology 13, no. 9 (2012): 888–899. 22797772 10.1038/ni.2370 PMC3985403

[82] J. Chen , M.‐C. Zhong , H. Guo , et al., “Slamf7 Is Critical for Phagocytosis of Haematopoietic Tumour Cells via Mac‐1 Integrin,” Nature 544, no. 7651 (2017): 493–497. 28424516 10.1038/nature22076 PMC5565268

[83] A. Aderem and D. M. Underhill , “Mechanisms of Phagocytosis in Macrophages,” Annual Review of Immunology 17, no. 1 (1999): 593–623. 10.1146/annurev.immunol.17.1.593 10358769

[84] F. Nimmerjahn and J. V. Ravetch , “Fcγ Receptors as Regulators of Immune Responses,” Nature Reviews Immunology 8, no. 1 (2008): 34–47. 10.1038/nri2206 18064051

[85] J. E. Bakema and M. Van Egmond , “Fc Receptor‐Dependent Mechanisms of Monoclonal Antibody Therapy of Cancer,” Fc Receptors (2014): 373–392. 10.1007/978-3-319-07911-0_17 25116109

[86] P. Bruhns , “Properties of Mouse and Human Igg Receptors and Their Contribution to Disease Models,” Blood, The Journal of the American Society of Hematology 119, no. 24 (2012): 5640–5649. 10.1182/blood-2012-01-380121 22535666

[87] M. Daëron , “Fc Receptor Biology,” Annual Review of Immunology 15, no. 1 (1997): 203–234. 10.1146/annurev.immunol.15.1.203 9143687

[88] M. T. Crowley , P. S. Costello , C. J. Fitzer‐Attas , et al., “A Critical Role for Syk in Signal Transduction and Phagocytosis Mediated by Fcγ Receptors on Macrophages,” Journal of Experimental Medicine 186, no. 7 (1997): 1027–1039. 9314552 10.1084/jem.186.7.1027 PMC2199061

[89] Y. Mao and C. Finnemann Silvia , “Regulation of Phagocytosis by Rho Gtpases,” Small GTPases 6, no. 2 (2015): 89–99. 25941749 10.4161/21541248.2014.989785 PMC4601285

[90] A. Getahun and J. C. Cambier , “Of Itim S, Itam S, and Itam Is: Revisiting Immunoglobulin Fc Receptor Signaling,” Immunological Reviews 268, no. 1 (2015): 66–73. 26497513 10.1111/imr.12336 PMC4621791

[91] A. Beers Stephen , R. French Ruth , C. Chan HT , et al., “Antigenic Modulation Limits the Efficacy of Anti‐Cd20 Antibodies: Implications for Antibody Selection,” Blood, The Journal of the American Society of Hematology 115, no. 25 (2010): 5191–5201. 10.1182/blood-2010-01-263533 20223920

[92] A. Bergtold , D. Desai Dharmesh , A. Gavhane , et al., “Cell Surface Recycling of Internalized Antigen Permits Dendritic Cell Priming of B Cells,” Immunity 23, no. 5 (2005): 503–514. 16286018 10.1016/j.immuni.2005.09.013

[93] P. Budde , N. Bewarder , V. Weinrich , et al., “Tyrosine‐Containing Sequence Motifs of the Human Immunoglobulin G Receptors Fcriib1 and Fcriib2 Essential for Endocytosis and Regulation of Calcium Flux in B Cells,” Journal of Biological Chemistry 269, no. 48 (1994): 30636–30644. 7527034

[94] S. Nagata , “Apoptosis and Clearance of Apoptotic Cells,” Annual Review of Immunology 36, no. 1 (2018): 489–517. 10.1146/annurev-immunol-042617-053010 29400998

[95] V. A. Fadok , D. R. Voelker , P. A. Campbell , et al., “Exposure of Phosphatidylserine on the Surface of Apoptotic Lymphocytes Triggers Specific Recognition and Removal by Macrophages,” Journal Of Immunology (Baltimore, Md: 1950) 148, no. 7 (1992): 2207–2216. 1545126

[96] R. B. Birge , S. Boeltz , S. Kumar , et al., “Phosphatidylserine Is a Global Immunosuppressive Signal in Efferocytosis, Infectious Disease, and Cancer,” Cell Death & Differentiation 23, no. 6 (2016): 962–978. 26915293 10.1038/cdd.2016.11 PMC4987730

[97] A. Leventis Peter and S. Grinstein , “The Distribution and Function of Phosphatidylserine in Cellular Membranes,” Annual Review of Biophysics 39, no. 1 (2010): 407–427. 10.1146/annurev.biophys.093008.131234 20192774

[98] G. Van Meer , “Dynamic Transbilayer Lipid Asymmetry,” Cold Spring Harbor Perspectives in Biology 3, no. 5 (2011): a004671. 21436058 10.1101/cshperspect.a004671 PMC3101844

[99] A. Kharitonenkov , Z. Chen , I. Sures , et al., “A Family of Proteins That Inhibit Signalling Through Tyrosine Kinase Receptors,” Nature 386, no. 6621 (1997): 181–186. 9062191 10.1038/386181a0

[100] Y. Fujioka , T. Matozaki , T. Noguchi , et al., “A Novel Membrane Glycoprotein, Shps‐1, That Binds the Sh2‐Domain‐Containing Protein Tyrosine Phosphatase Shp‐2 in Response to Mitogens and Cell Adhesion,” Molecular and Cellular Biology 16, no. 12 (1996): 6887–6899. 8943344 10.1128/mcb.16.12.6887 PMC231692

[101] A. Veillette , E. Thibaudeau , and S. Latour , “High Expression of Inhibitory Receptor Shps‐1 and Its Association With Protein‐Tyrosine Phosphatase Shp‐1 in Macrophages,” Journal of Biological Chemistry 273, no. 35 (1998): 22719–22728. 9712903 10.1074/jbc.273.35.22719

[102] Z. Jiang , H. Sun , J. Yu , et al., “Targeting Cd47 for Cancer Immunotherapy,” Journal of Hematology & Oncology 14, no. 1 (2021): 180. 34717705 10.1186/s13045-021-01197-w PMC8557524

[103] C. Jiang , H. Sun , Z. Jiang , et al., “Targeting the Cd47/Sirpα Pathway in Malignancies: Recent Progress, Difficulties and Future Perspectives,” Frontiers in Oncology 14 (2024): 1378647. 39040441 10.3389/fonc.2024.1378647 PMC11261161

[104] L. R. Silverman , D. R. Mckenzie , B. L. Peterson , et al., “Further Analysis of Trials With Azacitidine in Patients With Myelodysplastic Syndrome: Studies 8421, 8921, and 9221 by the Cancer and Leukemia Group B,” Journal of Clinical Oncology 24, no. 24 (2006): 3895–3903. 16921040 10.1200/JCO.2005.05.4346

[105] L. R. Silverman , E. P. Demakos , B. L. Peterson , et al., “Randomized Controlled Trial of Azacitidine in Patients With the Myelodysplastic Syndrome: A Study of the Cancer and Leukemia Group B,” Journal of Clinical Oncology 20, no. 10 (2002): 2429–2440. 12011120 10.1200/JCO.2002.04.117

[106] A. Sallman David , M. Al Malki Monzr , S. Asch Adam , et al., “Magrolimab in Combination With Azacitidine in Patients With Higher‐Risk Myelodysplastic Syndromes: Final Results of a Phase Ib Study,” Journal of Clinical Oncology 41, no. 15 (2023): 2815–2826. 36888930 10.1200/JCO.22.01794 PMC10414740

[107] Z. Yang , Y. Peng , W. Guo , et al., “Pd‐L1 and Cd47 Co‐Expression Predicts Survival and Enlightens Future Dual‐Targeting Immunotherapy in Non‐Small Cell Lung Cancer,” Thorac Cancer 12, no. 11 (2021): 1743–1751. 33979899 10.1111/1759-7714.13989 PMC8169290

[108] Y. C. Chen , W. Shi , J. J. Shi , et al., “Progress of Cd47 Immune Checkpoint Blockade Agents in Anticancer Therapy: A Hematotoxic Perspective,” Journal of Cancer Research and Clinical Oncology 148, no. 1 (2022): 1–14. 34609596 10.1007/s00432-021-03815-z PMC11800891

[109] Q. Chen , X. Guo , and W. Ma , “Opportunities and Challenges of Cd47‐Targeted Therapy in Cancer Immunotherapy,” Oncology Research 32, no. 1 (2023): 49–60. 38188674 10.32604/or.2023.042383 PMC10767231

[110] I. Mellman , G. Coukos , and G. Dranoff , “Cancer Immunotherapy Comes of Age,” Nature 480, no. 7378 (2011): 480–489. 22193102 10.1038/nature10673 PMC3967235

[111] L. Mazzarella , B. A. Duso , D. Trapani , et al., “The Evolving Landscape of ‘Next‐Generation’ Immune Checkpoint Inhibitors: A Review,” European Journal of Cancer 117 (2019): 14–31. 31229946 10.1016/j.ejca.2019.04.035

[112] M. Moehler , H. Xiao , I. Blum Steven , et al., “Health‐Related Quality of Life With Nivolumab Plus Chemotherapy versus Chemotherapy in Patients With Advanced Gastric/Gastroesophageal Junction Cancer or Esophageal Adenocarcinoma From Checkmate 649,” Journal of Clinical Oncology 41, no. 35 (2023): 5388–5399. 37713657 10.1200/JCO.23.00170 PMC10713185

[113] Y. J. Bang , Y. K. Kang , D. V. Catenacci , et al., “Pembrolizumab Alone or in Combination With Chemotherapy as First‐Line Therapy for Patients With Advanced Gastric or Gastroesophageal Junction Adenocarcinoma: Results From the Phase Ii Nonrandomized Keynote‐059 Study,” Gastric Cancer 22, no. 4 (2019): 828–837. 30911859 10.1007/s10120-018-00909-5 PMC6570680

[114] C. Robert , L. Thomas , I. Bondarenko , et al., “Ipilimumab Plus Dacarbazine for Previously Untreated Metastatic Melanoma,” New England Journal of Medicine 364, no. 26 (2011): 2517–2526. 21639810 10.1056/NEJMoa1104621

[115] Y. Jiang , M. Chen , H. Nie , et al., “Pd‐1 and Pd‐L1 in Cancer Immunotherapy: Clinical Implications and Future Considerations,” Human Vaccines & Immunotherapeutics 15, no. 5 (2019): 1111–1122. 30888929 10.1080/21645515.2019.1571892 PMC6605868

[116] Z. Zhu , H. Zhang , B. Chen , et al., “Pd‐L1‐Mediated Immunosuppression in Glioblastoma Is Associated With the Infiltration and M2‐Polarization of Tumor‐Associated Macrophages,” Frontiers in Immunology 11 (2020): 588552. 33329573 10.3389/fimmu.2020.588552 PMC7734279

[117] H. Yang , Q. Zhang , M. Xu , et al., “Ccl2‐Ccr2 Axis Recruits Tumor Associated Macrophages to Induce Immune Evasion Through Pd‐1 Signaling in Esophageal Carcinogenesis,” Molecular Cancer 19, no. 1 (2020): 41. 32103760 10.1186/s12943-020-01165-x PMC7045401

[118] B. Tan , X. Shi , J. Zhang , et al., “Inhibition of Rspo‐Lgr4 Facilitates Checkpoint Blockade Therapy by Switching Macrophage Polarization,” Cancer Research 78, no. 17 (2018): 4929–4942. 29967265 10.1158/0008-5472.CAN-18-0152

[119] N. Liu , J. Zhang , M. Yin , et al., “Inhibition of Xct Suppresses the Efficacy of Anti‐Pd‐1/L1 Melanoma Treatment Through Exosomal Pd‐L1‐Induced Macrophage M2 Polarization,” Molecular Therapy 29, no. 7 (2021): 2321–2334. 33744468 10.1016/j.ymthe.2021.03.013 PMC8261162

[120] W. Xie , L. J. Medeiros , S. Li , et al., “Pd‐1/Pd‐L1 Pathway and Its Blockade in Patients With Classic Hodgkin Lymphoma and Non‐Hodgkin Large‐Cell Lymphomas,” Current Hematologic Malignancy Reports 15, no. 4 (2020): 372–381. 32394185 10.1007/s11899-020-00589-y

[121] L. Xia , Y. Liu , and Y. Wang , “Pd‐1/Pd‐L1 Blockade Therapy in Advanced Non‐Small‐Cell Lung Cancer: Current Status and Future Directions,” The Oncologist 24, no. 1 (2019): S31–s41. 30819829 10.1634/theoncologist.2019-IO-S1-s05 PMC6394772

[122] K. Masuda , H. Horinouchi , M. Tanaka , et al., “Efficacy of Anti‐Pd‐1 Antibodies in Nsclc Patients With an Egfr Mutation and High Pd‐L1 Expression,” Journal of Cancer Research and Clinical Oncology 147, no. 1 (2021): 245–251. 32705363 10.1007/s00432-020-03329-0 PMC7810613

[123] D. J. Olson , Z. Eroglu , B. Brockstein , et al., “Pembrolizumab plus Ipilimumab Following Anti‐Pd‐1/L1 Failure in Melanoma,” Journal of Clinical Oncology 39, no. 24 (2021): 2647–2655. 33945288 10.1200/JCO.21.00079 PMC8376314

[124] P. Du Rusquec , O. De Calbiac , and M. Robert , “Clinical Utility of Pembrolizumab in the Management of Advanced Solid Tumors: An Evidence‐Based Review on the Emerging New Data,” Cancer Manag Res 11 (2019): 4297–4312. 31190995 10.2147/CMAR.S151023 PMC6527794

[125] Y. Agata , A. Kawasaki , H. Nishimura , et al., “Expression of the Pd‐1 Antigen on the Surface of Stimulated Mouse T and B Lymphocytes,” International Immunology 8, no. 5 (1996): 765–772. 8671665 10.1093/intimm/8.5.765

[126] J. Inokuchi and M. Eto , “Profile of Pembrolizumab in the Treatment of Patients With Unresectable or Metastatic Urothelial Carcinoma,” Cancer Manag Res 11 (2019): 4519–4528. 31191013 10.2147/CMAR.S167708 PMC6526676

[127] G. Kwok , T. C. Yau , J. W. Chiu , et al., “Pembrolizumab (Keytruda),” Human Vaccines & Immunotherapeutics 12, no. 11 (2016): 2777–2789. 27398650 10.1080/21645515.2016.1199310 PMC5137544

[128] M. Reck , D. Rodríguez‐Abreu , A. G. Robinson , et al., “Pembrolizumab versus Chemotherapy for Pd‐L1‐Positive Non‐Small‐Cell Lung Cancer,” New England Journal of Medicine 375, no. 19 (2016): 1823–1833. 27718847 10.1056/NEJMoa1606774

[129] Y. Ishida , Y. Agata , K. Shibahara , et al., “Induced Expression of Pd‐1, a Novel Member of the Immunoglobulin Gene Superfamily, Upon Programmed Cell Death,” Embo Journal 11, no. 11 (1992): 3887–3895. 1396582 10.1002/j.1460-2075.1992.tb05481.x PMC556898

[130] S. L. Topalian , J. M. Taube , and D. M. Pardoll , “Neoadjuvant Checkpoint Blockade for Cancer Immunotherapy,” Science 367, no. 6477 (2020), 10.1126/science.aax0182. PMC7789854 32001626

[131] V. Longo , O. Brunetti , A. Azzariti , et al., “Strategies to Improve Cancer Immune Checkpoint Inhibitors Efficacy, Other than Abscopal Effect: A Systematic Review,” Cancers (Basel) 11, no. 4 (2019), 10.3390/cancers11040539. PMC6521062 30991686

[132] T. N. Gide , J. S. Wilmott , R. A. Scolyer , et al., “Primary and Acquired Resistance to Immune Checkpoint Inhibitors in Metastatic Melanoma,” Clinical Cancer Research 24, no. 6 (2018): 1260–1270. 29127120 10.1158/1078-0432.CCR-17-2267

[133] C. M. Fares , E. M. Van Allen , C. G. Drake , et al., “Mechanisms of Resistance to Immune Checkpoint Blockade: Why Does Checkpoint Inhibitor Immunotherapy Not Work for all Patients?,” American Society of Clinical Oncology Educational Book 39 (2019): 147–164. 31099674 10.1200/EDBK_240837

[134] A. Carretero‐González , D. Lora , I. Ghanem , et al., “Analysis of Response Rate With Anti Pd1/Pd‐L1 Monoclonal Antibodies in Advanced Solid Tumors: A Meta‐Analysis of Randomized Clinical Trials,” Oncotarget 9, no. 9 (2018): 8706–8715. 29492229 10.18632/oncotarget.24283 PMC5823578

[135] Y. Nakamura , “Biomarkers for Immune Checkpoint Inhibitor‐Mediated Tumor Response and Adverse Events,” Front Med (Lausanne) 6 (2019): 119. 31192215 10.3389/fmed.2019.00119 PMC6549005

[136] X. Chen , Q. Lu , H. Zhou , et al., “A Membrane‐Associated Mhc‐I Inhibitory Axis for Cancer Immune Evasion,” Cell 186, no. 18 (2023): 3903–3920. e21. 37557169 10.1016/j.cell.2023.07.016 PMC10961051

[137] K. Yamamoto , A. Venida , J. Yano , et al., “Autophagy Promotes Immune Evasion of Pancreatic Cancer by Degrading Mhc‐I,” Nature 581, no. 7806 (2020): 100–105. 32376951 10.1038/s41586-020-2229-5 PMC7296553

[138] K. Dhatchinamoorthy , J. D. Colbert , and K. L. Rock , “Cancer Immune Evasion Through Loss of Mhc Class I Antigen Presentation,” Frontiers in Immunology 12 (2021): 636568. 33767702 10.3389/fimmu.2021.636568 PMC7986854

[139] X. Wu , T. Li , R. Jiang , et al., “Targeting Mhc‐I Molecules for Cancer: Function, Mechanism, and Therapeutic Prospects,” Molecular Cancer 22, no. 1 (2023): 194. 38041084 10.1186/s12943-023-01899-4 PMC10693139

[140] R. W. Lentz , M. D. Colton , S. S. Mitra , et al., “Innate Immune Checkpoint Inhibitors: The Next Breakthrough in Medical Oncology?,” Molecular Cancer Therapeutics 20, no. 6 (2021): 961–974. 33850005 10.1158/1535-7163.MCT-21-0041 PMC9028741

[141] C. L. Dulberger , C. P. Mcmurtrey , A. Hölzemer , et al., “Human Leukocyte Antigen F Presents Peptides and Regulates Immunity Through Interactions With Nk Cell Receptors,” Immunity 46, no. 6 (2017): 1018–1029. e7. 28636952 10.1016/j.immuni.2017.06.002 PMC5523829

[142] T. Zeller , I. A. Münnich , R. Windisch , et al., “Perspectives of Targeting Lilrb1 in Innate and Adaptive Immune Checkpoint Therapy of Cancer,” Frontiers in Immunology 14 (2023): 1240275. 37781391 10.3389/fimmu.2023.1240275 PMC10533923

[143] N. T. Young , E. C. Waller , R. Patel , et al., “The Inhibitory Receptor Lilrb1 Modulates the Differentiation and Regulatory Potential of Human Dendritic Cells,” Blood 111, no. 6 (2008): 3090–3096. 18094328 10.1182/blood-2007-05-089771

[144] X. Kang , J. Kim , M. Deng , et al., “Inhibitory Leukocyte Immunoglobulin‐Like Receptors: Immune Checkpoint Proteins and Tumor Sustaining Factors,” Cell Cycle 15, no. 1 (2016): 25–40. 26636629 10.1080/15384101.2015.1121324 PMC4825776

[145] A. Naji , C. Menier , G. Maki , et al., “Neoplastic B‐Cell Growth Is Impaired by Hla‐G/Ilt2 Interaction,” Leukemia 26, no. 8 (2012): 1889–1892. 22441169 10.1038/leu.2012.62

[146] Z. Hu , Q. Zhang , Z. He , et al., “Mhc1/Lilrb1 Axis as an Innate Immune Checkpoint for Cancer Therapy,” Frontiers in Immunology 15 (2024): 1421092. 38911856 10.3389/fimmu.2024.1421092 PMC11190085

[147] D. T. W. Van , H.‐M. Chen , P.‐Y. Pan , et al., “Lilrb Receptor‐Mediated Regulation of Myeloid Cell Maturation and Function,” Cancer Immunology, Immunotherapy 66 (2017): 1079–1087. 28638976 10.1007/s00262-017-2023-x PMC5591173

[148] T. Sinthuwiwat , S. Buranapraditkun , W. Kamolvisit , et al., “A Lilrb1 Variant With a Decreased Ability to Phosphorylate Shp‐1 Leads to Autoimmune Diseases,” Scientific Reports 12, no. 1 (2022): 15420. 36104364 10.1038/s41598-022-19334-x PMC9474825

[149] P. Bruhns , F. Vely , O. Malbec , et al., “Molecular Basis of the Recruitment of the Sh2 Domain‐Containing Inositol 5‐Phosphatases Ship1 and Ship2 by Fcgamma Riib,” Journal of Biological Chemistry 275, no. 48 (2000): 37357–37364. 11016922 10.1074/jbc.M003518200

[150] E. Bernit , E. Jean , B. Marlot , et al., “Hla‐F and Lilrb1 Genetic Polymorphisms Associated With Alloimmunisation in Sickle Cell Disease,” International Journal of Molecular Sciences 24, no. 17 (2023), 10.3390/ijms241713591. PMC10487752 37686397

[151] M. Daëron , S. Jaeger , and L. Du Pasquier , “Immunoreceptor Tyrosine‐Based Inhibition Motifs: A Quest in the Past and Future,” Immunological Reviews 224 (2008): 11–43. 18759918 10.1111/j.1600-065X.2008.00666.x

[152] J. Middelburg , S. Ghaffari , T. A. W. Schoufour , et al., “The Mhc‐E Peptide Ligands for Checkpoint Cd94/Nkg2a Are Governed by Inflammatory Signals, Whereas Lilrb1/2 Receptors Are Peptide Indifferent,” Cell reports 42, no. 12 (2023). 10.1016/j.celrep.2023.113516 38048225

[153] G.‐Y. Chen , K. Brown Nicholas , P. Zheng , et al., “Siglec‐G/10 in Self–Nonself Discrimination of Innate and Adaptive Immunity,” Glycobiology 24 (2014): 800–806. 24996822 10.1093/glycob/cwu068 PMC4116048

[154] S. J. Pirruccello and L. TW , “The Human B Cell‐Associated Antigen Cd24 Is a Single Chain Sialoglycoprotein,” Journal of immunology (Baltimore, Md: 1950) 136, no. 10 (1986): 3779–3784. 2939133

[155] T. Springer , G. Galfre , D. S. Secher , et al., “Monoclonal Xenogeneic Antibodies to Murine Cell Surface Antigens: Identification of Novel Leukocyte Differentiation Antigens,” European Journal of Immunology 8, no. 8 (1978): 539–551. 81133 10.1002/eji.1830080802

[156] A. Barkal Amira , E. Brewer Rachel , M. Markovic , et al., “Cd24 Signalling Through Macrophage Siglec‐10 Is a Target for Cancer Immunotherapy,” Nature 572, no. 7769 (2019): 392–396. 31367043 10.1038/s41586-019-1456-0 PMC6697206

[157] W. Chen , C. Han , B. Xie , et al., “Induction of Siglec‐G by Rna Viruses Inhibits the Innate Immune Response by Promoting Rig‐I Degradation,” Cell 152, no. 3 (2013): 467–478. 23374343 10.1016/j.cell.2013.01.011

[158] G. Y. Chen , X. Chen , S. King , et al., “Amelioration of Sepsis by Inhibiting Sialidase‐Mediated Disruption of the Cd24‐Siglecg Interaction,” Nature Biotechnology 29, no. 5 (2011): 428–435. 10.1038/nbt.1846 PMC4090080 21478876

[159] G. Y. Chen , J. Tang , P. Zheng , et al., “Cd24 and Siglec‐10 Selectively Repress Tissue Damage‐Induced Immune Responses,” Science 323, no. 5922 (2009): 1722–1725. 19264983 10.1126/science.1168988 PMC2765686

[160] T. Toubai , G. Hou , N. Mathewson , et al., “Siglec‐G‐Cd24 Axis Controls the Severity of Graft‐Versus‐Host Disease in Mice,” Blood 123, no. 22 (2014): 3512–3523. 24695850 10.1182/blood-2013-12-545335 PMC4041170

[161] P. R. Crocker , J. C. Paulson , and A. Varki , “Siglecs and Their Roles in the Immune System,” Nature Reviews Immunology 7, no. 4 (2007): 255–266. 10.1038/nri2056 17380156

[162] C. L. Abram and C. A. Lowell , “Shp1 Function in Myeloid Cells,” J Leukoc Biol 102, no. 3 (2017): 657–675. 28606940 10.1189/jlb.2MR0317-105R PMC5557645

[163] J. Dietrich , M. Cella , and M. Colonna , “Ig‐Like Transcript 2 (Ilt2)/Leukocyte Ig‐Like Receptor 1 (Lir1) Inhibits Tcr Signaling and Actin Cytoskeleton Reorganization,” Journal of Immunology 166, no. 4 (2001): 2514–2521. 10.4049/jimmunol.166.4.2514 11160312

[164] A. A. Barkal , R. E. Brewer , M. Markovic , et al., “Cd24 Signalling Through Macrophage Siglec‐10 Is a Target for Cancer Immunotherapy,” Nature 572, no. 7769 (2019): 392–396. 31367043 10.1038/s41586-019-1456-0 PMC6697206

[165] P. A. Oldenborg , A. Zheleznyak , Y. F. Fang , et al., “Role of Cd47 as a Marker of Self on Red Blood Cells,” Science 288, no. 5473 (2000): 2051–2054. 10856220 10.1126/science.288.5473.2051

[166] A. N. Barclay and T. K. Van Den Berg , “The Interaction Between Signal Regulatory Protein Alpha (Sirpα) and Cd47: Structure, Function, and Therapeutic Target,” Annual Review of Immunology 32 (2014): 25–50. 10.1146/annurev-immunol-032713-120142 24215318

[167] R. Medzhitov and A. Janeway Jr Charles , “Decoding the Patterns of Self and Nonself by the Innate Immune System,” Science 296, no. 5566 (2002): 298–300. 11951031 10.1126/science.1068883

[168] M. P. Chao , I. L. Weissman , and R. Majeti , “The Cd47‐Sirpα Pathway in Cancer Immune Evasion and Potential Therapeutic Implications,” Current Opinion in Immunology 24, no. 2 (2012): 225–232. 22310103 10.1016/j.coi.2012.01.010 PMC3319521

[169] D. Leone Robert and D. Powell Jonathan , “Metabolism of Immune Cells in Cancer,” Nature Reviews Cancer 20, no. 9 (2020): 516–531. 32632251 10.1038/s41568-020-0273-y PMC8041116

[170] B. Yingjie , L. I. Wei , D. M. Kremer , et al., “Cancer Slc43a2 Alters T Cell Methionine Metabolism and Histone Methylation,” Nature 585, no. 7824 (2020): 277–282. 32879489 10.1038/s41586-020-2682-1 PMC7486248

[171] D. Buck Michael , T. Sowell Ryan , M. Kaech Susan , et al., “Metabolic Instruction of Immunity,” Cell 169, no. 4 (2017): 570–586. 28475890 10.1016/j.cell.2017.04.004 PMC5648021

[172] A. Viola , F. Munari , R. Sánchez‐Rodríguez , et al., “The Metabolic Signature of Macrophage Responses,” Frontiers in Immunology 10 (2019): 1462. 31333642 10.3389/fimmu.2019.01462 PMC6618143

[173] Z. Cai , W. Li , M. Brenner , et al., “Branched‐Chain Ketoacids Derived From Cancer Cells Modulate Macrophage Polarization and Metabolic Reprogramming,” Frontiers in Immunology 13 (2022): 966158. 36311795 10.3389/fimmu.2022.966158 PMC9606345

[174] Q. Zhao , Z. Chu , L. Zhu , et al., “2‐Deoxy‐D‐Glucose Treatment Decreases Anti‐Inflammatory M2 Macrophage Polarization in Mice With Tumor and Allergic Airway Inflammation,” Frontiers in Immunology 8 (2017): 637. 28620389 10.3389/fimmu.2017.00637 PMC5451502

[175] G. G. Chen , P. Y. M. Woo , S. C. P. Ng , et al., “Impact of Metformin on Immunological Markers: Implication in Its Anti‐Tumor Mechanism,” Pharmacology & Therapeutics 213 (2020): 107585. 32473961 10.1016/j.pharmthera.2020.107585

[176] S. Wang , R. Liu , Q. Yu , et al., “Metabolic Reprogramming of Macrophages during Infections and Cancer,” Cancer Letters 452 (2019): 14–22. 30905817 10.1016/j.canlet.2019.03.015

[177] P. Su , Q. Wang , E. Bi , et al., “Enhanced Lipid Accumulation and Metabolism Are Required for the Differentiation and Activation of Tumor‐Associated Macrophages,” Cancer Research 80, no. 7 (2020): 1438–1450. 32015091 10.1158/0008-5472.CAN-19-2994 PMC7127942

[178] Y. Chen , J. Zhang , W. Cui , et al., “Cd36, a Signaling Receptor and Fatty Acid Transporter That Regulates Immune Cell Metabolism and Fate,” Journal of Experimental Medicine 219, no. 6 (2022), 10.1084/jem.20211314. PMC9022290 35438721

[179] W. Xiang , R. Shi , X. Kang , et al., “Monoacylglycerol Lipase Regulates Cannabinoid Receptor 2‐Dependent Macrophage Activation and Cancer Progression,” Nature Communications 9, no. 1 (2018): 2574. 10.1038/s41467-018-04999-8 PMC6030061 29968710

[180] L. Liang , H. He , S. Jiang , et al., “Tiam2 Contributes to Osimertinib Resistance, Cell Motility, and Tumor‐Associated Macrophage M2‐Like Polarization in Lung Adenocarcinoma,” International Journal of Molecular Sciences 23, no. 18 (2022), 10.3390/ijms231810415. PMC9499457 36142328

[181] P. Salvador and R.‐P. Sergio , “Macrophage Polarization and Reprogramming in Acute Inflammation: A Redox Perspective,” Antioxidants 11, no. 7 (2022): 1394. 35883885 10.3390/antiox11071394 PMC9311967

[182] S. C. Huang , A. M. Smith , B. Everts , et al., “Metabolic Reprogramming Mediated by the Mtorc2‐Irf4 Signaling Axis Is Essential for Macrophage Alternative Activation,” Immunity 45, no. 4 (2016): 817–830. 27760338 10.1016/j.immuni.2016.09.016 PMC5535820

[183] R. Geiger , J. C. Rieckmann , T. Wolf , et al., “L‐Arginine Modulates T Cell Metabolism and Enhances Survival and Anti‐Tumor Activity,” Cell 167, no. 3 (2016): 829–842. e13. 27745970 10.1016/j.cell.2016.09.031 PMC5075284

[184] M. M. Kaneda , K. S. Messer , N. Ralainirina , et al., “Pi3kγ Is a Molecular Switch That Controls Immune Suppression,” Nature 539, no. 7629 (2016): 437–442. 27642729 10.1038/nature19834 PMC5479689

[185] P. Foubert , M. M. Kaneda , and J. A. Varner , “Pi3kγ Activates Integrin Α(4) and Promotes Immune Suppressive Myeloid Cell Polarization during Tumor Progression,” Cancer Immunology Research 5, no. 11 (2017): 957–968. 28963139 10.1158/2326-6066.CIR-17-0143 PMC6422969

[186] M. M. Kaneda , P. Cappello , A. V. Nguyen , et al., “Macrophage Pi3kγ Drives Pancreatic Ductal Adenocarcinoma Progression,” Cancer Discovery 6, no. 8 (2016): 870–885. 27179037 10.1158/2159-8290.CD-15-1346 PMC5091937

[187] A. C. Thomas and J. T. Mattila , “Of Mice and Men: Arginine Metabolism in Macrophages,” Frontiers in Immunology 5 (2014): 479. 25339954 10.3389/fimmu.2014.00479 PMC4188127

[188] M. Kaneda Megan , S. Messer Karen , R. Natacha , et al., “Pi3kγ Is a Molecular Switch That Controls Immune Suppression,” Nature 539, no. 7629 (2016): 437–442. 27642729 10.1038/nature19834 PMC5479689

[189] J. Q. Zhang , S. Zeng , G. A. Vitiello , et al., “Macrophages and Cd8(+) T Cells Mediate the Antitumor Efficacy of Combined Cd40 Ligation and Imatinib Therapy in Gastrointestinal Stromal Tumors,” Cancer Immunology Research 6, no. 4 (2018): 434–447. 29467128 10.1158/2326-6066.CIR-17-0345 PMC6203303

[190] P. S. Liu , Y. T. Chen , X. Li , et al., “Cd40 Signal Rewires Fatty Acid and Glutamine Metabolism for Stimulating Macrophage Anti‐Tumorigenic Functions,” Nature Immunology 24, no. 3 (2023): 452–462. 36823405 10.1038/s41590-023-01430-3 PMC9977680

[191] E. De Visser Karin and A. Joyce Johanna , “The Evolving Tumor Microenvironment: From Cancer Initiation to Metastatic Outgrowth,” Cancer Cell 41, no. 3 (2023): 374–403. 36917948 10.1016/j.ccell.2023.02.016

[192] J. Zuo , H. Yan , S. Qin , et al., “Extracellular Vesicles in Cancer Drug Resistance: Mechanistic Insights and Therapeutic Implications,” MedComm–Oncology 3, no. 4 (2024): e94.

[193] S. Baig Mirza , A. Roy , S. Rajpoot , et al., “Tumor‐Derived Exosomes in the Regulation of Macrophage Polarization,” Inflammation Research 69 (2020): 435–451. 32162012 10.1007/s00011-020-01318-0

[194] F. Ruivo Carolina , B. Adem , M. Silva , et al., “The Biology of Cancer Exosomes: Insights and New Perspectives,” Cancer Research 77, no. 23 (2017): 6480–6488. 29162616 10.1158/0008-5472.CAN-17-0994

[195] A. Mori Marcelo , G. Ludwig Raissa , R. Garcia‐Martin , et al., “Extracellular Mirnas: From Biomarkers to Mediators of Physiology and Disease,” Cell Metabolism 30, no. 4 (2019): 656–673. 31447320 10.1016/j.cmet.2019.07.011 PMC6774861

[196] S. Zhao , Y. Mi , B. Guan , et al., “Tumor‐Derived Exosomal Mir‐934 Induces Macrophage M2 Polarization to Promote Liver Metastasis of Colorectal Cancer,” Journal of Hematology & Oncology 13 (2020): 1–19. 33213490 10.1186/s13045-020-00991-2 PMC7678301

[197] H. Shinohara , Y. Kuranaga , M. Kumazaki , et al., “Regulated Polarization of Tumor‐Associated Macrophages by Mir‐145 via Colorectal Cancer–Derived Extracellular Vesicles,” The Journal of Immunology 199, no. 4 (2017): 1505–1515. 28696255 10.4049/jimmunol.1700167

[198] D. Gerloff , J. Lützkendorf , K. C. Moritz Rose , et al., “Melanoma‐Derived Exosomal Mir‐125b‐5p Educates Tumor Associated Macrophages (Tams) by Targeting Lysosomal Acid Lipase a (Lipa),” Cancers 12, no. 2 (2020): 464. 32079286 10.3390/cancers12020464 PMC7072270

[199] T. Lee Jeannie and M. S. Bartolomei , “X‐Inactivation, Imprinting, and Long Noncoding Rnas in Health and Disease,” Cell 152, no. 6 (2013): 1308–1323. 23498939 10.1016/j.cell.2013.02.016

[200] A. Fatica and I. Bozzoni , “Long Non‐Coding Rnas: New Players in Cell Differentiation and Development,” Nature Reviews Genetics 15, no. 1 (2014): 7–21. 10.1038/nrg3606 24296535

[201] Y. He , X.‐M. Meng , C. Huang , et al., “Long Noncoding Rnas: Novel Insights Into Hepatocelluar Carcinoma,” Cancer Letters 344, no. 1 (2014): 20–27. 24183851 10.1016/j.canlet.2013.10.021

[202] T. Kogure , K. Yan Irene , W.‐L. Lin , et al., “Extracellular Vesicle–Mediated Transfer of a Novel Long Noncoding Rna Tuc339: A Mechanism of Intercellular Signaling in Human Hepatocellular Cancer,” Genes & Cancer 4, no. 7–8 (2013): 261–272. 24167654 10.1177/1947601913499020 PMC3807642

[203] X. Li , Y. I. Lei , M. Wu , et al., “Regulation of Macrophage Activation and Polarization by Hcc‐Derived Exosomal Lncrna Tuc339,” International Journal of Molecular Sciences 19, no. 10 (2018): 2958. 30274167 10.3390/ijms19102958 PMC6213212

[204] Y. Liang , X. Song , Y. Li , et al., “Lncrna Bcrt1 Promotes Breast Cancer Progression by Targeting Mir‐1303/Ptbp3 Axis,” Molecular Cancer 19 (2020): 1–20. 32384893 10.1186/s12943-020-01206-5 PMC7206728

[205] L. S. Kristensen , T. Jakobsen , H. Hager , et al., “The Emerging Roles of Circrnas in Cancer and Oncology,” Nature Reviews Clinical Oncology 19, no. 3 (2022): 188–206. 10.1038/s41571-021-00585-y 34912049

[206] Q. Sun , X. Lei , and X. Yang , “Circrnas as Upstream Regulators of Mirna//Hmga2 Axis in Human Cancer,” Pharmacology & Therapeutics 263 (2024): 108711. 39222752 10.1016/j.pharmthera.2024.108711

[207] Q. Lin , Q. Qi , S. Hou , et al., “Exosomal Circular Rna Hsa_Circ_007293 Promotes Proliferation, Migration, Invasion, and Epithelial‐Mesenchymal Transition of Papillary Thyroid Carcinoma Cells Through Regulation of the Microrna‐653‐5p/Paired Box 6 Axis,” Bioengineered 12, no. 2 (2021): 10136–10149. 34866540 10.1080/21655979.2021.2000745 PMC8809932

[208] T. Michel , F. Hentges , and J. Zimmer , “Consequences of the Crosstalk Between Monocytes/Macrophages and Natural Killer Cells,” Frontiers in Immunology 3 (2013): 403. 23316194 10.3389/fimmu.2012.00403 PMC3539656

[209] S. Sivori , D. Pende , L. Quatrini , et al., “Nk Cells and Ilcs in Tumor Immunotherapy,” Molecular Aspects of Medicine 80 (2021): 100870. 32800530 10.1016/j.mam.2020.100870

[210] H. Gonzalez , C. Hagerling , and Z. Werb , “Roles of the Immune System in Cancer: From Tumor Initiation to Metastatic Progression,” Genes & Development 32, no. 19–20 (2018): 1267–1284. 30275043 10.1101/gad.314617.118 PMC6169832

[211] T. Krneta , A. Gillgrass , S. Poznanski , et al., “M2‐Polarized and Tumor‐Associated Macrophages Alter Nk Cell Phenotype and Function in a Contact‐Dependent Manner,” Journal of Leucocyte Biology 101, no. 1 (2017): 285–295. 10.1189/jlb.3A1215-552R 27493241

[212] S. Platonova , J. Cherfils‐Vicini , D. Damotte , et al., “Profound Coordinated Alterations of Intratumoral Nk Cell Phenotype and Function in Lung Carcinoma,” Cancer Research 71, no. 16 (2011): 5412–5422. 21708957 10.1158/0008-5472.CAN-10-4179

[213] M. Kloss , P. Decker , M. Baltz Katrin , et al., “Interaction of Monocytes With Nk Cells Upon Toll‐Like Receptor‐Induced Expression of the Nkg2d Ligand Mica,” The Journal of Immunology 181, no. 10 (2008): 6711–6719. 18981088 10.4049/jimmunol.181.10.6711

[214] L. Peng , J. Zhang , Y. Teng , et al., “Tumor‐Associated Monocytes/Macrophages Impair Nk‐Cell Function via Tgfβ1 in Human Gastric Cancer,” Cancer Immunology Research 5, no. 3 (2017): 248–256. 28148545 10.1158/2326-6066.CIR-16-0152

[215] R. Castriconi , C. Cantoni , C. M. Della , et al., “Transforming Growth Factor Β1 Inhibits Expression of Nkp30 and Nkg2d Receptors: Consequences for the Nk‐Mediated Killing of Dendritic Cells,” Proceedings of the National Academy of Sciences 100, no. 7 (2003): 4120–4125. 10.1073/pnas.0730640100 PMC153058 12646700

[216] M. Gallazzi , D. Baci , L. Mortara , et al., “Prostate Cancer Peripheral Blood Nk Cells Show Enhanced Cd9, Cd49a, Cxcr4, Cxcl8, Mmp‐9 Production and Secrete Monocyte‐Recruiting and Polarizing Factors,” Frontiers in Immunology 11 (2021): 586126. 33569050 10.3389/fimmu.2020.586126 PMC7868409

[217] S. Malekghasemi , J. Majidi , A. Baghbanzadeh , et al., “Tumor‐Associated Macrophages: Protumoral Macrophages in Inflammatory Tumor Microenvironment,” Advanced Pharmaceutical Bulletin 10, no. 4 (2020): 556. 33062602 10.34172/apb.2020.066 PMC7539304

[218] A. Ponzetta , R. Carriero , S. Carnevale , et al., “Neutrophils Driving Unconventional T Cells Mediate Resistance Against Murine Sarcomas and Selected Human Tumors,” Cell 178, no. 2 (2019): 346–360. e24. 31257026 10.1016/j.cell.2019.05.047 PMC6630709

[219] L. Wu and H.‐F. Zhang Xiang , “Tumor‐Associated Neutrophils and Macrophages—Heterogenous but Not Chaotic,” Frontiers in Immunology 11 (2020): 553967. 33343560 10.3389/fimmu.2020.553967 PMC7738476

[220] J. Kim and J.‐S. Bae , “Tumor‐Associated Macrophages and Neutrophils in Tumor Microenvironment,” Mediators of Inflammation 1 (2016): 6058147. 10.1155/2016/6058147 PMC4757693 26966341

[221] S.‐L. Zhou , Z.‐J. Zhou , Z.‐Q. Hu , et al., “Tumor‐Associated Neutrophils Recruit Macrophages and T‐Regulatory Cells to Promote Progression of Hepatocellular Carcinoma and Resistance to Sorafenib,” Gastroenterology 150, no. 7 (2016): 1646–1658. e17. 26924089 10.1053/j.gastro.2016.02.040

[222] P. Tsou , H. Katayama , E. J. Ostrin , et al., “The Emerging Role of B Cells in Tumor Immunity,” Cancer Research 76, no. 19 (2016): 5597–5601. 27634765 10.1158/0008-5472.CAN-16-0431

[223] H. Nelson Brad , “Cd20+ B Cells: The Other Tumor‐Infiltrating Lymphocytes,” The Journal of Immunology 185, no. 9 (2010): 4977–4982. 20962266 10.4049/jimmunol.1001323

[224] J. Gunderson Andrew , M. M. Kaneda , T. Tsujikawa , et al., “Bruton Tyrosine Kinase–Dependent Immune Cell Cross‐Talk Drives Pancreas Cancer,” Cancer Discovery 6, no. 3 (2016): 270–285. 26715645 10.1158/2159-8290.CD-15-0827 PMC4783268

[225] C. Fremd , F. Schuetz , C. Sohn , et al., “B Cell‐Regulated Immune Responses in Tumor Models and Cancer Patients,” Oncoimmunology 2, no. 7 (2013): e25443. 24073382 10.4161/onci.25443 PMC3782133

[226] S. I. Wang , W. Liu , D. Ly , et al., “Tumor‐Infiltrating B Cells: Their Role and Application in Anti‐Tumor Immunity in Lung Cancer,” Cellular & Molecular Immunology 16, no. 1 (2019): 6–18. 29628498 10.1038/s41423-018-0027-x PMC6318290

[227] T. Audzevich , R. Bashford‐Rogers , A. Mabbott Neil , et al., “Pre/Pro‐B Cells Generate Macrophage Populations during Homeostasis and Inflammation,” Proceedings of the National Academy of Sciences 114, no. 20 (2017): E3954–E3963. 10.1073/pnas.1616417114 PMC5441795 28461481

[228] S. R. Dannenmann , J. Thielicke , M. Stöckli , et al., “Tumor‐Associated Macrophages Subvert T‐Cell Function and Correlate With Reduced Survival in Clear Cell Renal Cell Carcinoma,” Oncoimmunology 2, no. 3 (2013): e23562. 23687622 10.4161/onci.23562 PMC3655740

[229] S. Gordon and F. O. Martinez , “Alternative Activation of Macrophages: Mechanism and Functions,” Immunity 32, no. 5 (2010): 593–604. 20510870 10.1016/j.immuni.2010.05.007

[230] Z. He and S. Zhang , “Tumor‐Associated Macrophages and Their Functional Transformation in the Hypoxic Tumor Microenvironment,” Frontiers in Immunology 12 (2021): 741305. 34603327 10.3389/fimmu.2021.741305 PMC8481680

[231] H. M. Knochelmann , C. J. Dwyer , S. R. Bailey , et al., “When Worlds Collide: Th17 and Treg Cells in Cancer and Autoimmunity,” Cellular & Molecular Immunology 15, no. 5 (2018): 458–469. 29563615 10.1038/s41423-018-0004-4 PMC6068176

[232] D. Waniczek , Z. Lorenc , M. Śnietura , et al., “Tumor‐Associated Macrophages and Regulatory T Cells Infiltration and the Clinical Outcome in Colorectal Cancer,” Archivum Immunologiae Et Therapiae Experimentalis 65, no. 5 (2017): 445–454. 28343267 10.1007/s00005-017-0463-9 PMC5602054

[233] L. La Fleur , J. Botling , and F. He , “Targeting Marco and Il37r on Immunosuppressive Macrophages in Lung Cancer Blocks Regulatory T Cells and Supports Cytotoxic Lymphocyte Function,” Cancer Research 81, no. 4 (2021): 956–967. 33293426 10.1158/0008-5472.CAN-20-1885

[234] Q. Zhu , X. Wu , Y. Wu , et al., “Interaction Between Treg Cells and Tumor‐Associated Macrophages in the Tumor Microenvironment of Epithelial Ovarian Cancer,” Oncology Reports 36, no. 6 (2016): 3472–3478. 27748885 10.3892/or.2016.5136

[235] Q. Wu , W. Zhou , S. Yin , et al., “Blocking Triggering Receptor Expressed on Myeloid Cells‐1‐Positive Tumor‐Associated Macrophages Induced by Hypoxia Reverses Immunosuppression and Anti‐Programmed Cell Death Ligand 1 Resistance in Liver Cancer,” Hepatology 70, no. 1 (2019): 198–214. 30810243 10.1002/hep.30593 PMC6618281

[236] T. Fujimura , Y. Kambayashi , Y. Fujisawa , et al., “Tumor‐Associated Macrophages: Therapeutic Targets for Skin Cancer,” Frontiers in oncology 8 (2018): 3. 29410946 10.3389/fonc.2018.00003 PMC5787130

[237] S. Furudate , T. Fujimura , Y. Kambayashi , et al., “Immunomodulatory Effect of Imiquimod Through Ccl22 Produced by Tumor‐Associated Macrophages in B16f10 Melanomas,” Anticancer Research 37, no. 7 (2017): 3461–3471. 28668835 10.21873/anticanres.11714

[238] D. Wang , L. Yang , D. Yue , et al., “Macrophage‐Derived Ccl22 Promotes an Immunosuppressive Tumor Microenvironment via Il‐8 in Malignant Pleural Effusion,” Cancer Letters 452 (2019): 244–253. 30928379 10.1016/j.canlet.2019.03.040

[239] J. M. Carbó , T. E. León , J. Font‐Díaz , et al., “Pharmacologic Activation of Lxr Alters the Expression Profile of Tumor‐Associated Macrophages and the Abundance of Regulatory T Cells in the Tumor Microenvironment,” Cancer Research 81, no. 4 (2021): 968–985. 33361391 10.1158/0008-5472.CAN-19-3360

[240] H. Raskov , A. Orhan , J. P. Christensen , et al., “Cytotoxic Cd8(+) T Cells in Cancer and Cancer Immunotherapy,” British Journal of Cancer 124, no. 2 (2021): 359–367. 32929195 10.1038/s41416-020-01048-4 PMC7853123

[241] J. Reiser and A. Banerjee , “Effector, Memory, and Dysfunctional Cd8(+) T Cell Fates in the Antitumor Immune Response,” Journal of Immunology Research 2016 (2016): 8941260. 27314056 10.1155/2016/8941260 PMC4893440

[242] E. Peranzoni , J. Lemoine , L. Vimeux , et al., “Macrophages Impede Cd8 T Cells From Reaching Tumor Cells and Limit the Efficacy of Anti‐Pd‐1 Treatment,” PNAS 115, no. 17 (2018): E4041–E4050. 29632196 10.1073/pnas.1720948115 PMC5924916

[243] E. M. Garrido‐Martin , T. W. P. Mellows , J. Clarke , et al., “M1(Hot) Tumor‐Associated Macrophages Boost Tissue‐Resident Memory T Cells Infiltration and Survival in Human Lung Cancer,” Journal for ImmunoTherapy of Cancer 8, no. 2 (2020), 10.1136/jitc-2020-000778. PMC7375465 32699181

[244] X. Dai , L. Lu , S. Deng , et al., “Usp7 Targeting Modulates Anti‐Tumor Immune Response by Reprogramming Tumor‐Associated Macrophages in Lung Cancer,” Theranostics 10, no. 20 (2020): 9332–9347. 32802195 10.7150/thno.47137 PMC7415808

[245] W. Fang , T. Zhou , H. Shi , et al., “Progranulin Induces Immune Escape in Breast Cancer via Up‐Regulating Pd‐L1 Expression on Tumor‐Associated Macrophages (Tams) and Promoting Cd8(+) T Cell Exclusion,” Journal of Experimental & Clinical Cancer Research 40, no. 1 (2021): 4. 33390170 10.1186/s13046-020-01786-6 PMC7780622

[246] H. Lin , S. Wei , E. M. Hurt , et al., “Host Expression of Pd‐L1 Determines Efficacy of Pd‐L1 Pathway Blockade‐Mediated Tumor Regression,” Journal of Clinical Investigation 128, no. 2 (2018): 805–815. 29337305 10.1172/JCI96113 PMC5785251

[247] M. Pascual‐García , E. Bonfill‐Teixidor , E. Planas‐Rigol , et al., “Lif Regulates Cxcl9 in Tumor‐Associated Macrophages and Prevents Cd8(+) T Cell Tumor‐Infiltration Impairing Anti‐Pd1 Therapy,” Nature Communications 10, no. 1 (2019): 2416. 10.1038/s41467-019-10369-9 PMC6559950 31186412

[248] J. S. Dolina , N. Van Braeckel‐Budimir , G. D. Thomas , et al., “Cd8(+) T Cell Exhaustion in Cancer,” Frontiers in Immunology 12 (2021): 715234. 34354714 10.3389/fimmu.2021.715234 PMC8330547

[249] I. S. Chan and A. J. Ewald , “The Changing Role of Natural Killer Cells in Cancer Metastasis,” Journal of Clinical Investigation 132, no. 6 (2022), 10.1172/jci143762. PMC8920322 35289318

[250] Y. Li , A. Sharma , J. Maciaczyk , et al., “Recent Development in Nkt‐Based Immunotherapy of Glioblastoma: From Bench to Bedside,” International Journal of Molecular Sciences 23, no. 3 (2022), 10.3390/ijms23031311. PMC8835986 35163235

[251] H. Bayatipoor , S. Mehdizadeh , R. Jafarpour , et al., “Role of Nkt Cells in Cancer Immunotherapy‐From Bench to Bed,” Medical Oncology 40, no. 1 (2022): 29. 36460881 10.1007/s12032-022-01888-5

[252] R. M. Mcewen‐Smith , M. Salio , and V. Cerundolo , “The Regulatory Role of Invariant Nkt Cells in Tumor Immunity,” Cancer Immunology Research 3, no. 5 (2015): 425–435. 25941354 10.1158/2326-6066.CIR-15-0062 PMC4430818

[253] K. Mortezaee and J. Majidpoor , “Nk and Cells With Nk‐Like Activities in Cancer Immunotherapy‐Clinical Perspectives,” Medical Oncology 39, no. 9 (2022): 131. 35716327 10.1007/s12032-022-01735-7

[254] L. Song , S. Asgharzadeh , J. Salo , et al., “Valpha24‐Invariant Nkt Cells Mediate Antitumor Activity via Killing of Tumor‐Associated Macrophages,” Journal of Clinical Investigation 119, no. 6 (2009): 1524–1536. 19411762 10.1172/JCI37869 PMC2689106

[255] E. A. Bae , H. Seo , I. K. Kim , et al., “Roles of Nkt Cells in Cancer Immunotherapy,” Archives of Pharmacal Research 42, no. 7 (2019): 543–548. 30859410 10.1007/s12272-019-01139-8

[256] R. Reantragoon , A. J. Corbett , I. G. Sakala , et al., “Antigen‐Loaded Mr1 Tetramers Define T Cell Receptor Heterogeneity in Mucosal‐Associated Invariant T Cells,” Journal of Experimental Medicine 210, no. 11 (2013): 2305–2320. 24101382 10.1084/jem.20130958 PMC3804952

[257] L. Le Bourhis , E. Martin , I. Pguillet , et al., “Antimicrobial Activity of Mucosal‐Associated Invariant T Cells,” Nature Immunology 11, no. 8 (2010): 701–708. 20581831 10.1038/ni.1890

[258] S. Huang , E. Martin , S. Kim , et al., “Mr1 Antigen Presentation to Mucosal‐Associated Invariant T Cells Was Highly Conserved in Evolution,” PNAS 106, no. 20 (2009): 8290–8295. 19416870 10.1073/pnas.0903196106 PMC2688861

[259] L. Zheng , S. Qin , W. Si , et al., “Pan‐Cancer Single‐Cell Landscape of Tumor‐Infiltrating T Cells,” Science 374, no. 6574 (2021): abe6474. 34914499 10.1126/science.abe6474

[260] C. Zheng , L. Zheng , J. K. Yoo , et al., “Landscape of Infiltrating T Cells in Liver Cancer Revealed by Single‐Cell Sequencing,” Cell 169, no. 7 (2017): 1342–1356. e16. 28622514 10.1016/j.cell.2017.05.035

[261] A. Kurioka , J. E. Ussher , C. Cosgrove , et al., “Mait Cells Are Licensed Through Granzyme Exchange to Kill Bacterially Sensitized Targets,” Mucosal Immunology 8, no. 2 (2015): 429–440. 25269706 10.1038/mi.2014.81 PMC4288950

[262] E. Billerbeck , Y. H. Kang , L. Walker , et al., “Analysis of Cd161 Expression on Human Cd8+ T Cells Defines a Distinct Functional Subset With Tissue‐Homing Properties,” PNAS 107, no. 7 (2010): 3006–3011. 20133607 10.1073/pnas.0914839107 PMC2840308

[263] N. A. Gherardin , L. Loh , L. Admojo , et al., “Enumeration, Functional Responses and Cytotoxic Capacity of Mait Cells in Newly Diagnosed and Relapsed Multiple Myeloma,” Scientific Reports 8, no. 1 (2018): 4159. 29515123 10.1038/s41598-018-22130-1 PMC5841305

[264] T. Leng , H. D. Akther , C. P. Hackstein , et al., “Tcr and Inflammatory Signals Tune Human Mait Cells to Exert Specific Tissue Repair and Effector Functions,” Cell reports 28, no. 12 (2019): 3077–3091. e5. 31533032 10.1016/j.celrep.2019.08.050 PMC6899450

[265] J. E. Ussher , M. Bilton , E. Attwod , et al., “Cd161++ Cd8+ T Cells, Including the Mait Cell Subset, Are Specifically Activated by Il‐12+Il‐18 in a Tcr‐Independent Manner,” European Journal of Immunology 44, no. 1 (2014): 195–203. 24019201 10.1002/eji.201343509 PMC3947164

[266] J. Dias , E. Leeansyah , and J. K. Sandberg , “Multiple Layers of Heterogeneity and Subset Diversity in Human Mait Cell Responses to Distinct Microorganisms and to Innate Cytokines,” PNAS 114, no. 27 (2017): E5434–E5443. 28630305 10.1073/pnas.1705759114 PMC5502643

[267] J. Kelly , Y. Minoda , T. Meredith , et al., “Chronically Stimulated Human Mait Cells Are Unexpectedly Potent Il‐13 Producers,” Immunology and Cell Biology 97, no. 8 (2019): 689–699. 31323167 10.1111/imcb.12281 PMC6790710

[268] M. Duan , S. Goswami , J. Y. Shi , et al., “Activated and Exhausted Mait Cells Foster Disease Progression and Indicate Poor Outcome in Hepatocellular Carcinoma,” Clinical Cancer Research 25, no. 11 (2019): 3304–3316. 30723143 10.1158/1078-0432.CCR-18-3040

[269] B. Ruf , M. Bruhns , S. Babaei , et al., “Tumor‐Associated Macrophages Trigger Mait Cell Dysfunction at the Hcc Invasive Margin,” Cell 186, no. 17 (2023): 3686–3705. e32. 37595566 10.1016/j.cell.2023.07.026 PMC10461130

[270] B. Silva‐Santos , K. Serre , and H. Norell , “Γδ T Cells in Cancer,” Nature Reviews Immunology 15, no. 11 (2015): 683–691. 10.1038/nri3904 26449179

[271] B. Tamuli , S. Sharma , M. Patkar , et al., “Key Players of Immunosuppression in Epithelial Malignancies: Tumor‐Infiltrating Myeloid Cells and Γδ T Cells,” Cancer Rep (Hoboken) 7, no. 5 (2024): e2066. 38703051 10.1002/cnr2.2066 PMC11069128

[272] J. A. Mathews , D. I. Kasahara , L. Ribeiro , et al., “Γδ T Cells Are Required for M2 Macrophage Polarization and Resolution of Ozone‐Induced Pulmonary Inflammation in Mice,” PLoS ONE 10, no. 7 (2015): e0131236. 26135595 10.1371/journal.pone.0131236 PMC4489797

[273] M. Miyashita , T. Shimizu , E. Ashihara , et al., “Strategies to Improve the Antitumor Effect of Γδ T Cell Immunotherapy for Clinical Application,” International Journal of Molecular Sciences 22, no. 16 (2021): 8910. 34445615 10.3390/ijms22168910 PMC8396358

[274] Y. Mao , S. Yin , J. Zhang , et al., “A New Effect of Il‐4 on Human Γδ T Cells: Promoting Regulatory Vδ1 T Cells via Il‐10 Production and Inhibiting Function of Vδ2 T Cells,” Cellular & Molecular Immunology 13, no. 2 (2016): 217–228. 25942601 10.1038/cmi.2015.07 PMC4786628

[275] D. Wesch , D. Kabelitz , and H. H. Oberg , “Tumor Resistance Mechanisms and Their Consequences on Γδ T Cell Activation,” Immunological Reviews 298, no. 1 (2020): 84–98. 33048357 10.1111/imr.12925

[276] D. I. Gabrilovich , V. Bronte , S. H. Chen , et al., “The Terminology Issue for Myeloid‐Derived Suppressor Cells,” Cancer Research 67, no. 1 (2007): 425. author reply 26. 17210725 10.1158/0008-5472.CAN-06-3037 PMC1941787

[277] J. C. Rodrigues , G. C. Gonzalez , L. Zhang , et al., “Normal Human Monocytes Exposed to Glioma Cells Acquire Myeloid‐Derived Suppressor Cell‐Like Properties,” Neuro‐oncol 12, no. 4 (2010): 351–365. 20308313 10.1093/neuonc/nop023 PMC2940603

[278] Y. Zhao , J. Du , and X. Shen , “Targeting Myeloid‐Derived Suppressor Cells in Tumor Immunotherapy: Current, Future and Beyond,” Frontiers In Immunology 14 (2023): 1157537. 37006306 10.3389/fimmu.2023.1157537 PMC10063857

[279] T. Condamine , I. Ramachandran , J. I. Youn , et al., “Regulation of Tumor Metastasis by Myeloid‐Derived Suppressor Cells,” Annual Review of Medicine 66 (2015): 97–110. 10.1146/annurev-med-051013-052304 PMC4324727 25341012

[280] J. E. Talmadge and D. I. Gabrilovich , “History of Myeloid‐Derived Suppressor Cells,” Nature Reviews Cancer 13, no. 10 (2013): 739–752. 24060865 10.1038/nrc3581 PMC4358792

[281] D. Marvel and D. I. Gabrilovich , “Myeloid‐Derived Suppressor Cells in the Tumor Microenvironment: Expect the Unexpected,” Journal of Clinical Investigation 125, no. 9 (2015): 3356–3364. 26168215 10.1172/JCI80005 PMC4588239

[282] S. Ugel , F. De Sanctis , S. Mandruzzato , et al., “Tumor‐Induced Myeloid Deviation: When Myeloid‐Derived Suppressor Cells Meet Tumor‐Associated Macrophages,” Journal of Clinical Investigation 125, no. 9 (2015): 3365–3376. 26325033 10.1172/JCI80006 PMC4588310

[283] V. Kumar , S. Patel , E. Tcyganov , et al., “The Nature of Myeloid‐Derived Suppressor Cells in the Tumor Microenvironment,” Trends in Immunology 37, no. 3 (2016): 208–220. 26858199 10.1016/j.it.2016.01.004 PMC4775398

[284] T. Celià‐Terrassa and M. K. Jolly , “Cancer Stem Cells and Epithelial‐to‐Mesenchymal Transition in Cancer Metastasis,” Cold Spring Harbor Perspectives in Medicine 10, no. 7 (2020), 10.1101/cshperspect.a036905. PMC7328448 31570380

[285] T. Celià‐Terrassa and Y. Kang , “Distinctive Properties of Metastasis‐Initiating Cells,” Genes & Development 30, no. 8 (2016): 892–908. 27083997 10.1101/gad.277681.116 PMC4840296

[286] T. Oskarsson , E. Batlle , and J. Massagu , “Metastatic Stem Cells: Sources, Niches, and Vital Pathways,” Cell Stem Cell 14, no. 3 (2014): 306–321. 24607405 10.1016/j.stem.2014.02.002 PMC3998185

[287] L. Müller , A. Tunger , I. Plesca , et al., “Bidirectional Crosstalk Between Cancer Stem Cells and Immune Cell Subsets,” Frontiers in Immunology 11 (2020): 140. 32117287 10.3389/fimmu.2020.00140 PMC7013084

[288] D. Bayik and J. D. Lathia , “Cancer Stem Cell‐Immune Cell Crosstalk in Tumour Progression,” Nature Reviews Cancer 21, no. 8 (2021): 526–536. 34103704 10.1038/s41568-021-00366-w PMC8740903

[289] R. G. Stein , S. Ebert , L. Schlahsa , et al., “Cognate Nonlytic Interactions Between Cd8(+) T Cells and Breast Cancer Cells Induce Cancer Stem Cell‐Like Properties,” Cancer Research 79, no. 7 (2019): 1507–1519. 30692216 10.1158/0008-5472.CAN-18-0387

[290] K. Li , H. Shi , B. Zhang , et al., “Myeloid‐Derived Suppressor Cells as Immunosuppressive Regulators and Therapeutic Targets in Cancer,” Signal Transduct Target Ther 6, no. 1 (2021): 362. 34620838 10.1038/s41392-021-00670-9 PMC8497485

[291] D. Roy , S. Bose , S. Pati , et al., “Gfi1/Hdac1‐Axis Differentially Regulates Immunosuppressive Cd73 in Human Tumor‐Associated Foxp3(+) Th17 and Inflammation‐Linked Th17 Cells,” European Journal of Immunology 51, no. 5 (2021): 1206–1217. 33555624 10.1002/eji.202048892

[292] F. Zeppernick , R. Ahmadi , B. Campos , et al., “Stem Cell Marker Cd133 Affects Clinical Outcome in Glioma Patients,” Clinical Cancer Research 14, no. 1 (2008): 123–129. 18172261 10.1158/1078-0432.CCR-07-0932

[293] C. Raggi , H. S. Mousa , M. Correnti , et al., “Cancer Stem Cells and Tumor‐Associated Macrophages: A Roadmap for Multitargeting Strategies,” Oncogene 35, no. 6 (2016): 671–682. 25961921 10.1038/onc.2015.132

[294] T. M. Yeung , S. C. Gandhi , and W. F. Bodmer , “Hypoxia and Lineage Specification of Cell Line‐Derived Colorectal Cancer Stem Cells,” PNAS 108, no. 11 (2011): 4382–4387. 21368208 10.1073/pnas.1014519107 PMC3060223

[295] H. Lu , K. R. Clauser , W. L. Tam , et al., “A Breast Cancer Stem Cell Niche Supported by Juxtacrine Signalling From Monocytes and Macrophages,” Nature Cell Biology 16, no. 11 (2014): 1105–1117. 25266422 10.1038/ncb3041 PMC4296514

[296] S. Wan , E. Zhao , and I. Kryczek , “Tumor‐Associated Macrophages Produce Interleukin 6 and Signal via Stat3 to Promote Expansion of Human Hepatocellular Carcinoma Stem Cells,” Gastroenterology 147, no. 6 (2014): 1393–1404. 25181692 10.1053/j.gastro.2014.08.039 PMC4253315

[297] Y. Tan , M. Wang , Y. Zhang , et al., “Tumor‐Associated Macrophages: A Potential Target for Cancer Therapy,” Frontiers in oncology 11 (2021): 693517. 34178692 10.3389/fonc.2021.693517 PMC8222665

[298] N. B. Hao , M. H. Lü , Y. H. Fan , et al., “Macrophages in Tumor Microenvironments and the Progression of Tumors,” Clinical & Developmental Immunology 2012 (2012): 948098. 22778768 10.1155/2012/948098 PMC3385963

[299] S. Luo , G. Yang , P. Ye , et al., “Macrophages Are a Double‐Edged Sword: Molecular Crosstalk Between Tumor‐Associated Macrophages and Cancer Stem Cells,” Biomolecules 12, no. 6 (2022), 10.3390/biom12060850. PMC9221070 35740975

[300] A. Wu , J. Wei , L. Y. Kong , et al., “Glioma Cancer Stem Cells Induce Immunosuppressive Macrophages/Microglia,” Neuro‐Oncology 12, no. 11 (2010): 1113–1125. 20667896 10.1093/neuonc/noq082 PMC3098021

[301] A. De Boeck , B. Y. Ahn , C. D'mello , et al., “Glioma‐Derived Il‐33 Orchestrates an Inflammatory Brain Tumor Microenvironment That Accelerates Glioma Progression,” Nature Communications 11, no. 1 (2020): 4997. 10.1038/s41467-020-18569-4 PMC7536425 33020472

[302] K. Gabrusiewicz , X. Li , J. Wei , et al., “Glioblastoma Stem Cell‐Derived Exosomes Induce M2 Macrophages and Pd‐L1 Expression on Human Monocytes,” Oncoimmunology 7, no. 4 (2018): e1412909. 29632728 10.1080/2162402X.2017.1412909 PMC5889290

[303] P. Allavena , E. Digifico , and C. Belgiovine , “Macrophages and Cancer Stem Cells: A Malevolent Alliance,” Molecular Medicine 27, no. 1 (2021): 121. 34583655 10.1186/s10020-021-00383-3 PMC8480058

[304] F. Zhang , P. Li , S. Liu , et al., “Β‐Catenin‐Ccl2 Feedback Loop Mediates Crosstalk Between Cancer Cells and Macrophages That Regulates Breast Cancer Stem Cells,” Oncogene 40, no. 39 (2021): 5854–5865. 34345015 10.1038/s41388-021-01986-0

[305] B. Guan , H. Li , J. Yao , et al., “Ccl3‐Ccr5 Axis Promotes Cell Migration and Invasion of Colon Adenocarcinoma via Akt Signaling Pathway,” Environmental Toxicology 38, no. 1 (2023): 172–184. 36346222 10.1002/tox.23675

[306] M. Novak , M. Koprivnikar Krajnc , B. Hrastar , et al., “Ccr5‐Mediated Signaling Is Involved in Invasion of Glioblastoma Cells in Its Microenvironment,” International Journal of Molecular Sciences 21, no. 12 (2020), 10.3390/ijms21124199. PMC7352708 32545571

[307] N. N. V. Radharani , A. S. Yadav , R. Nimma , et al., “Tumor‐Associated Macrophage Derived Il‐6 Enriches Cancer Stem Cell Population and Promotes Breast Tumor Progression via Stat‐3 Pathway,” Cancer Cell International 22, no. 1 (2022): 122. 35300689 10.1186/s12935-022-02527-9 PMC8932105

[308] H. R. Cho , N. Kumari , and H. Thi Vu , “Increased Antiangiogenic Effect by Blocking Ccl2‐Dependent Macrophages in a Rodent Glioblastoma Model: Correlation Study With Dynamic Susceptibility Contrast Perfusion Mri,” Scientific Reports 9, no. 1 (2019): 11085. 31366997 10.1038/s41598-019-47438-4 PMC6668454

[309] C. T. Shih , C. W. Shiau , Y. L. Chen , et al., “Td‐92, a Novel Erlotinib Derivative, Depletes Tumor‐Associated Macrophages in Non‐Small Cell Lung Cancer via Down‐Regulation of Csf‐1r and Enhances the Anti‐Tumor Effects of Anti‐Pd‐1,” Cancer Letters 498 (2021): 142–151. 33232786 10.1016/j.canlet.2020.10.043

[310] C. Ngambenjawong , M. Cieslewicz , J. G. Schellinger , et al., “Synthesis and Evaluation of Multivalent M2pep Peptides for Targeting Alternatively Activated M2 Macrophages,” J Control Release 224 (2016): 103–111. 26772876 10.1016/j.jconrel.2015.12.057 PMC4747818

[311] J. Zhu , H. Huang , H. Chen , et al., “Plerixafor and Granulocyte‐Colony‐Stimulating Factor for Mobilization of Hematopoietic Stem Cells for Autologous Transplantation in Chinese Patients With Non‐Hodgkin's Lymphoma: A Randomized Phase 3 Study,” Transfusion 58, no. 1 (2018): 81–87. 29238988 10.1111/trf.14426

[312] B. Bockorny , V. Semenisty , T. Macarulla , et al., “Bl‐8040, a Cxcr4 Antagonist, in Combination With Pembrolizumab and Chemotherapy for Pancreatic Cancer: The Combat Trial,” Nature Medicine 26, no. 6 (2020): 878–885. 10.1038/s41591-020-0880-x 32451495

[313] X. Peng , P. Hou , Y. Chen , et al., “Preclinical Evaluation of 3d185, a Novel Potent Inhibitor of Fgfr1/2/3 and Csf‐1r, in Fgfr‐Dependent and Macrophage‐Dominant Cancer Models,” Journal of Experimental & Clinical Cancer Research 38, no. 1 (2019): 372. 31438996 10.1186/s13046-019-1357-y PMC6704710

[314] T. Fujiwara , M. A. Yakoub , A. Chandler , et al., “Csf1/Csf1r Signaling Inhibitor Pexidartinib (Plx3397) Reprograms Tumor‐Associated Macrophages and Stimulates T‐Cell Infiltration in the Sarcoma Microenvironment,” Molecular Cancer Therapeutics 20, no. 8 (2021): 1388–1399. 34088832 10.1158/1535-7163.MCT-20-0591 PMC9336538

[315] M. H. Schleimann , M. L. Kobberø , L. K. Vibholm , et al., “Tlr9 Agonist Mgn1703 Enhances B Cell Differentiation and Function in Lymph Nodes,” EBioMedicine 45 (2019): 328–340. 31300344 10.1016/j.ebiom.2019.07.005 PMC6642412

[316] K. A. Reiss , M. G. Angelos , E. C. Dees , et al., “Car‐Macrophage Therapy for Her2‐Overexpressing Advanced Solid Tumors: A Phase 1 Trial,” Nature Medicine 31, no. 4 (2025): 1171–1182. 10.1038/s41591-025-03495-z 39920391

[317] Y. Chen , Z. Yu , X. Tan , et al., “Car‐Macrophage: A New Immunotherapy Candidate Against Solid Tumors,” Biomedicine & Pharmacotherapy 139 (2021): 111605. 33901872 10.1016/j.biopha.2021.111605

[318] A. M. Scott , G. Wiseman , S. Welt , et al., “A Phase I Dose‐Escalation Study of Sibrotuzumab in Patients With Advanced or Metastatic Fibroblast Activation Protein‐Positive Cancer,” Clinical Cancer Research 9, no. 5 (2003): 1639–1647. 12738716

[319] M. Provencio , E. Nadal , J. L. González‐Larriba , et al., “Perioperative Nivolumab and Chemotherapy in Stage Iii Non‐Small‐Cell Lung Cancer,” New England Journal of Medicine 389, no. 6 (2023): 504–513. 37379158 10.1056/NEJMoa2215530

[320] J. Naidoo , S. Antonia , Y. L. Wu , et al., “Brief Report: Durvalumab After Chemoradiotherapy in Unresectable Stage Iii Egfr‐Mutant Nsclc: A Post Hoc Subgroup Analysis From Pacific,” Journal of Thoracic Oncology 18, no. 5 (2023): 657–663. 36841540 10.1016/j.jtho.2023.02.009

[321] H. A. Tawbi , D. Schadendorf , E. J. Lipson , et al., “Relatlimab and Nivolumab versus Nivolumab in Untreated Advanced Melanoma,” New England Journal of Medicine 386, no. 1 (2022): 24–34. 34986285 10.1056/NEJMoa2109970 PMC9844513

[322] C. G. Kim , M. H. Hong , D. Kim , et al., “A Phase Ii Open‐Label Randomized Clinical Trial of Preoperative Durvalumab or Durvalumab Plus Tremelimumab in Resectable Head and Neck Squamous Cell Carcinoma,” Clinical Cancer Research 30, no. 10 (2024): 2097–2110. 38457288 10.1158/1078-0432.CCR-23-3249

[323] C. Gomez‐Roca , P. Cassier , D. Zamarin , et al., “Anti‐Csf‐1r Emactuzumab in Combination With Anti‐Pd‐L1 Atezolizumab in Advanced Solid Tumor Patients Naïve or Experienced for Immune Checkpoint Blockade,” Journal for ImmunoTherapy of Cancer 10, no. 5 (2022): e004076. 35577503 10.1136/jitc-2021-004076 PMC9114963

[324] J. Boutilier Ava and F. Elsawa Sherine , “Macrophage Polarization States in the Tumor Microenvironment,” International Journal of Molecular Sciences 22, no. 13 (2021): 6995. 34209703 10.3390/ijms22136995 PMC8268869

[325] C.‐T. Shih , C.‐W. Shiau , Y.‐L. Chen , et al., “Td‐92, a Novel Erlotinib Derivative, Depletes Tumor‐Associated Macrophages in Non‐Small Cell Lung Cancer via Down‐Regulation of Csf‐1r and Enhances the Anti‐Tumor Effects of Anti‐Pd‐1,” Cancer Letters 498 (2021): 142–151. 33232786 10.1016/j.canlet.2020.10.043

[326] X. Li , W. Yao , Y. A. Yuan , et al., “Targeting of Tumour‐Infiltrating Macrophages via Ccl2/Ccr2 Signalling as a Therapeutic Strategy Against Hepatocellular Carcinoma,” Gut 66, no. 1 (2017): 157–167. 26452628 10.1136/gutjnl-2015-310514

[327] M. Li , L. He , J. Zhu , et al., “Targeting Tumor‐Associated Macrophages for Cancer Treatment,” Cell & Bioscience 12, no. 1 (2022): 85. 35672862 10.1186/s13578-022-00823-5 PMC9172100

[328] R. Cho Hye , N. Kumari , H. Thi Vu , et al., “Increased Antiangiogenic Effect by Blocking Ccl2‐Dependent Macrophages in a Rodent Glioblastoma Model: Correlation Study With Dynamic Susceptibility Contrast Perfusion Mri,” Scientific Reports 9, no. 1 (2019): 11085. 31366997 10.1038/s41598-019-47438-4 PMC6668454

[329] S. Gordon and R. Taylor Philip , “Monocyte and Macrophage Heterogeneity,” Nature Reviews Immunology 5 (2005): 953–964. 10.1038/nri1733 16322748

[330] C. Ngambenjawong , M. Cieslewicz , G. Schellinger Joan , et al., “Synthesis and Evaluation of Multivalent M2pep Peptides for Targeting Alternatively Activated M2 Macrophages,” Journal of Controlled Release 224 (2016): 103–111. 26772876 10.1016/j.jconrel.2015.12.057 PMC4747818

[331] B. Kakoschky , T. Pleli , C. Schmithals , et al., “Selective Targeting of Tumor Associated Macrophages in Different Tumor Models,” PLoS ONE 13, no. 2 (2018): e0193015. 29447241 10.1371/journal.pone.0193015 PMC5814016

[332] M. Cieslewicz , J. Tang , L. Yu Jonathan , et al., “Targeted Delivery of Proapoptotic Peptides to Tumor‐Associated Macrophages Improves Survival,” Proceedings of the National Academy of Sciences 110, no. 40 (2013): 15919–15924. 10.1073/pnas.1312197110 PMC3791765 24046373

[333] R. Wesolowski , N. Sharma , L. Reebel , et al., “Phase Ib Study of the Combination of Pexidartinib (Plx3397), a Csf‐1r Inhibitor, and Paclitaxel in Patients With Advanced Solid Tumors,” Therapeutic Advances in Medical Oncology 11 (2019): 1758835919854238. 31258629 10.1177/1758835919854238 PMC6589951

[334] J. Y. Ao , X. D. Zhu , Z. T. Chai , et al., “Colony‐Stimulating Factor 1 Receptor Blockade Inhibits Tumor Growth by Altering the Polarization of Tumor‐Associated Macrophages in Hepatocellular Carcinoma,” Molecular Cancer Therapeutics 16, no. 8 (2017): 1544–1554. 28572167 10.1158/1535-7163.MCT-16-0866

[335] A. Vidyarthi , N. Khan , T. Agnihotri , et al., “Tlr‐3 Stimulation Skews M2 Macrophages to M1 Through Ifn‐Αβ Signaling and Restricts Tumor Progression,” Frontiers in Immunology 9 (2018): 1650. 30072995 10.3389/fimmu.2018.01650 PMC6060442

[336] K. Kapp , B. Volz , D. Oswald , et al., “Beneficial Modulation of the Tumor Microenvironment and Generation of Anti‐Tumor Responses by Tlr9 Agonist Lefitolimod Alone and in Combination With Checkpoint Inhibitors,” Oncoimmunology 8, no. 12 (2019): e1659096. 31741757 10.1080/2162402X.2019.1659096 PMC6844329

[337] Z. Liu , Y. Xie , Y. Xiong , et al., “Tlr 7/8 Agonist Reverses Oxaliplatin Resistance in Colorectal Cancer via Directing the Myeloid‐Derived Suppressor Cells to Tumoricidal M1‐Macrophages,” Cancer Letters 469 (2020): 173–185. 31629935 10.1016/j.canlet.2019.10.020

[338] W. Nie , G. Wu , J. Zhang , et al., “Responsive Exosome Nano‐Bioconjugates for Synergistic Cancer Therapy,” Angewandte Chemie International Edition 59, no. 5 (2020): 2018–2022. 31746532 10.1002/anie.201912524

[339] J. Wei , M. Hu , K. Huang , et al., “Roles of Proteoglycans and Glycosaminoglycans in Cancer Development and Progression,” International Journal of Molecular Sciences 21, no. 17 (2020), 10.3390/ijms21175983. PMC7504257 32825245

[340] J. Okrzeja , A. Karwowska , and A. Błachnio‐Zabielska , “The Role of Obesity, Inflammation and Sphingolipids in the Development of an Abdominal Aortic Aneurysm,” Nutrients 14, no. 12 (2022): 2438. 35745168 10.3390/nu14122438 PMC9229568

[341] M. Liu , J. Liu , Z. Liang , et al., “Car‐Macrophages and Car‐T Cells Synergistically Kill Tumor Cells in Vitro,” Cells 11, no. 22 (2022): 3692. 36429120 10.3390/cells11223692 PMC9688246

[342] K. Hadiloo , S. Taremi , M. Heidari , et al., “The Car Macrophage Cells, a Novel Generation of Chimeric Antigen‐Based Approach Against Solid Tumors,” Biomarker Research 11, no. 1 (2023): 103. 38017494 10.1186/s40364-023-00537-x PMC10685521

[343] S. Y. Li , Y. L. Guo , J. W. Tian , et al., “Anti‐Tumor Strategies by Harnessing the Phagocytosis of Macrophages,” Cancers (Basel) 15, no. 10 (2023): 2717. 37345054 10.3390/cancers15102717 PMC10216167

[344] Y. Liu , H. Tan , J. Dai , et al., “Targeting Macrophages in Cancer Immunotherapy: Frontiers and Challenges,” Journal of Advanced Research 76 (2025): 695–713. 39778768 10.1016/j.jare.2024.12.043 PMC12793739

[345] Y. R. Na , S. W. Kim , and S. H. Seok , “A New Era of Macrophage‐Based Cell Therapy,” Experimental & Molecular Medicine 55, no. 9 (2023): 1945–1954. 37653035 10.1038/s12276-023-01068-z PMC10545778

[346] S. M. Abdin , D. Paasch , M. Morgan , et al., “Cars and Beyond: Tailoring Macrophage‐Based Cell Therapeutics to Combat Solid Malignancies,” Journal for ImmunoTherapy of Cancer 9, no. 8 (2021): e002741. 34462325 10.1136/jitc-2021-002741 PMC8407221

[347] Q. Wang , X. Shao , Y. Zhang , et al., “Role of Tumor Microenvironment in Cancer Progression and Therapeutic Strategy,” Cancer medicine 12, no. 10 (2023): 11149–11165. 36807772 10.1002/cam4.5698 PMC10242329

[348] M. Kciuk , E. B. Yahya , I. Mohamed , et al., “Recent Advances in Molecular Mechanisms of Cancer Immunotherapy,” Cancers (Basel) 15, no. 10 (2023): 2721. 37345057 10.3390/cancers15102721 PMC10216302

[349] A. Chow , K. Perica , A. Klebanoff Christopher , et al., “Clinical Implications of T Cell Exhaustion for Cancer Immunotherapy,” Nature Reviews Clinical Oncology 19, no. 12 (2022): 775–790. 10.1038/s41571-022-00689-z PMC10984554 36216928

[350] Q. Deng , G. Han , N. Puebla‐Osorio , et al., “Characteristics of Anti‐Cd19 Car T Cell Infusion Products Associated With Efficacy and Toxicity in Patients With Large B Cell Lymphomas,” Nature Medicine 26, no. 12 (2020): 1878–1887. 10.1038/s41591-020-1061-7 PMC8446909 33020644

[351] G. Ferrer , Á.‐E. Damiana , and E. Manel , “Biological and Molecular Factors Predicting Response to Adoptive Cell Therapies in Cancer,” JNCI: Journal of the National Cancer Institute 114, no. 7 (2022): 930–939. 35438170 10.1093/jnci/djac088 PMC9275759

[352] A. Mantovani , P. Allavena , F. Marchesi , et al., “Macrophages as Tools and Targets in Cancer Therapy,” Nature Reviews Drug Discovery 21, no. 11 (2022): 799–820. 35974096 10.1038/s41573-022-00520-5 PMC9380983

[353] S. Chen , F. U. H. Saeed Abdullah , Q. Liu , et al., “Macrophages in Immunoregulation and Therapeutics,” Signal Transduction and Targeted Therapy 8, no. 1 (2023): 207. 37211559 10.1038/s41392-023-01452-1 PMC10200802

[354] C. Sloas , S. Gill , and M. Klichinsky , “Engineered Car‐Macrophages as Adoptive Immunotherapies for Solid Tumors,” Frontiers in Immunology 12 (2021): 783305. 34899748 10.3389/fimmu.2021.783305 PMC8652144

[355] Q. Liu , J. Li , H. Zheng , et al., “Adoptive Cellular Immunotherapy for Solid Neoplasms beyond Car‐T,” Molecular Cancer 22, no. 1 (2023): 28. 36750830 10.1186/s12943-023-01735-9 PMC9903509

[356] Z. Duan , Z. Li , Z. Wang , et al., “Chimeric Antigen Receptor Macrophages Activated Through Tlr4 or Ifn‐Γ Receptors Suppress Breast Cancer Growth by Targeting Vegfr2,” Cancer Immunology, Immunotherapy 72, no. 10 (2023): 3243–3257. 37438548 10.1007/s00262-023-03490-8 PMC10992605

[357] C. Sloas , S. Gill , and M. Klichinsky , “Engineered Car‐Macrophages as Adoptive Immunotherapies for Solid Tumors,” Frontiers in Immunology 12 (2021): 783305. 34899748 10.3389/fimmu.2021.783305 PMC8652144

[358] M. Klichinsky , M. Ruella , O. Shestova , et al., “Human Chimeric Antigen Receptor Macrophages for Cancer Immunotherapy,” Nature Biotechnology 38, no. 8 (2020): 947–953. 10.1038/s41587-020-0462-y PMC7883632 32361713

[359] Z. Niu , G. Chen , W. Chang , et al., “Chimeric Antigen Receptor‐Modified Macrophages Trigger Systemic Anti‐Tumour Immunity,” Journal of Pathology 253, no. 3 (2021): 247–257. 33140856 10.1002/path.5585

[360] L. Zhang , L. Tian , X. Dai , et al., “Pluripotent Stem Cell‐Derived Car‐Macrophage Cells With Antigen‐Dependent Anti‐Cancer Cell Functions,” Journal of Hematology & Oncology 13, no. 1 (2020): 153. 33176869 10.1186/s13045-020-00983-2 PMC7656711

[361] Y. Chen , X. Zhu , H. Liu , et al., “The Application of Her2 and Cd47 Car‐Macrophage in Ovarian Cancer,” Journal of Translational Medicine 21, no. 1 (2023): 654. 37740183 10.1186/s12967-023-04479-8 PMC10517545

[362] G. Ferrer , D. Álvarez‐Errico , and M. Esteller , “Biological and Molecular Factors Predicting Response to Adoptive Cell Therapies in Cancer,” JNCI: Journal of the National Cancer Institute 114, no. 7 (2022): 930–939. 35438170 10.1093/jnci/djac088 PMC9275759

[363] L. Labanieh and C. L. Mackall , “Car Immune Cells: Design Principles, Resistance and the Next Generation,” Nature 614, no. 7949 (2023): 635–648. 36813894 10.1038/s41586-023-05707-3

[364] D. J. Irvine , M. V. Maus , D. J. Mooney , et al., “The Future of Engineered Immune Cell Therapies,” Science 378, no. 6622 (2022): 853–858. 36423279 10.1126/science.abq6990 PMC9919886

[365] R. Na Yi , W. Kim Sang , and H. Seok Seung , “A New Era of Macrophage‐Based Cell Therapy,” Experimental & Molecular Medicine 55, no. 9 (2023): 1945–1954. 37653035 10.1038/s12276-023-01068-z PMC10545778

[366] C. He , J. Mansilla‐Soto , N. Khanra , et al., “Cd19 Car Antigen Engagement Mechanisms and Affinity Tuning,” Science Immunology 8, no. 81 (2023): eadf1426. 36867678 10.1126/sciimmunol.adf1426 PMC10228544

[367] X. Wang , S. Su , Y. Zhu , et al., “Metabolic Reprogramming via Acod1 Depletion Enhances Function of Human Induced Pluripotent Stem Cell‐Derived Car‐Macrophages in Solid Tumors,” Nature Communications 14, no. 1 (2023): 5778. 10.1038/s41467-023-41470-9 PMC10507032 37723178

[368] W. Zhang , Q. Han , Y. Ding , et al., “Bcl6 Drives Stem‐Like Memory Macrophages Differentiation to Foster Tumor Progression,” Cellular and Molecular Life Sciences 80, no. 1 (2022): 14. 36542153 10.1007/s00018-022-04660-0 PMC9771855

[369] J. H. Cho , J. J. Collins , and W. W. Wong , “Universal Chimeric Antigen Receptors for Multiplexed and Logical Control of T Cell Responses,” Cell 173, no. 6 (2018): 1426–1438. e11. 29706540 10.1016/j.cell.2018.03.038 PMC5984158

[370] J. Kwon , J. Kang , A. Jo , et al., “Single‐Cell Mapping of Combinatorial Target Antigens for Car Switches Using Logic Gates,” Nature Biotechnology 41 (2023): 1593–1605. 11. 10.1038/s41587-023-01686-y 36797491

[371] D. Xue , S. Lu , H. Zhang , et al., “Induced Pluripotent Stem Cell‐Derived Engineered T Cells, Natural Killer Cells, Macrophages, and Dendritic Cells in Immunotherapy,” Trends in Biotechnology 41, no. 7 (2023): 907–922. 36858941 10.1016/j.tibtech.2023.02.003

[372] S. Ferrari , E. Valeri , A. Conti , et al., “Genetic Engineering Meets Hematopoietic Stem Cell Biology for Next‐Generation Gene Therapy,” Cell Stem Cell 30, no. 5 (2023): 549–570. 37146580 10.1016/j.stem.2023.04.014

[373] J. Li , P. Chen , and W. Ma , “The Next Frontier in Immunotherapy: Potential and Challenges of Car‐Macrophages,” Experimental hematology & oncology 13, no. 1 (2024): 76. 39103972 10.1186/s40164-024-00549-9 PMC11302330

[374] K. T. Roybal and W. A. Lim , “Synthetic Immunology: Hacking Immune Cells to Expand Their Therapeutic Capabilities,” Annual Review of Immunology 35 (2017): 229–253. 10.1146/annurev-immunol-051116-052302 PMC5555230 28446063

[375] P. Yousefpour , K. Ni , and D. J. Irvine , “Targeted Modulation of Immune Cells and Tissues Using Engineered Biomaterials,” Nat Rev Bioeng 1, no. 2 (2023): 107–124. 37772035 10.1038/s44222-022-00016-2 PMC10538251

[376] E. M. Bressler , S. Adams , R. Liu , et al., “Boolean Logic in Synthetic Biology and Biomaterials: Towards Living Materials in Mammalian Cell Therapeutics,” Clinical and Translational Medicine 13, no. 7 (2023): e1244. 37386762 10.1002/ctm2.1244 PMC10310979

[377] L. Ma , A. Hostetler , D. M. Morgan , et al., “Vaccine‐Boosted Car T Crosstalk With Host Immunity to Reject Tumors With Antigen Heterogeneity,” Cell 186, no. 15 (2023): 3148–3165. e20. 37413990 10.1016/j.cell.2023.06.002 PMC10372881

[378] M. E. Rodriguez‐Ruiz , I. Vitale , K. J. Harrington , et al., “Immunological Impact of Cell Death Signaling Driven by Radiation on the Tumor Microenvironment,” Nature Immunology 21, no. 2 (2020): 120–134. 31873291 10.1038/s41590-019-0561-4

[379] Q. Wang , Y. Wang , J. Ding , et al., “A Bioorthogonal System Reveals Antitumour Immune Function of Pyroptosis,” Nature 579, no. 7799 (2020): 421–426. 32188939 10.1038/s41586-020-2079-1

[380] L. Galluzzi , J. Humeau , A. Buqu , et al., “Immunostimulation With Chemotherapy in the Era of Immune Checkpoint Inhibitors,” Nature Reviews Clinical Oncology 17, no. 12 (2020): 725–741. 10.1038/s41571-020-0413-z 32760014

[381] T. Sklarz , P. Guan , M. Gohil , et al., “Mtorc2 Regulates Multiple Aspects of Nkt‐Cell Development and Function,” European Journal of Immunology 47, no. 3 (2017): 516–526. 28078715 10.1002/eji.201646343 PMC5656007

[382] D. Samarkanova , S. Cox , D. Hernandez , et al., “Cord Blood Platelet Rich Plasma Derivatives for Clinical Applications in Non‐Transfusion Medicine,” Frontiers in immunology 11 (2020): 942. 32536916 10.3389/fimmu.2020.00942 PMC7266986

[383] B. Motais , S. Charvátová , Z. Walek , et al., “Selection, Expansion, and Unique Pretreatment of Allogeneic Human Natural Killer Cells With Anti‐Cd38 Monoclonal Antibody for Efficient Multiple Myeloma Treatment,” Cells 10, no. 5 (2021), 10.3390/cells10050967. PMC8143171 33919155

[384] L. J. Burns , D. J. Weisdorf , T. E. Defor , et al., “Enhancement of the Anti‐Tumor Activity of a Peripheral Blood Progenitor Cell Graft by Mobilization With Interleukin 2 Plus Granulocyte Colony‐Stimulating Factor in Patients With Advanced Breast Cancer,” Experimental Hematology 28, no. 1 (2000): 96–103. 10658681 10.1016/s0301-472x(99)00129-0

[385] Y. M. Liao , T. H. Hung , J. K. Tung , et al., “Low Expression of Il‐15 and Nkt in Tumor Microenvironment Predicts Poor Outcome of Mycn‐Non‐Amplified Neuroblastoma,” J Pers Med 11, no. 2 (2021), 10.3390/jpm11020122. PMC7918138 33668573

[386] A. Heczey , A. N. Courtney , A. Montalbano , et al., “Anti‐Gd2 Car‐Nkt Cells in Patients With Relapsed or Refractory Neuroblastoma: An Interim Analysis,” Nature Medicine 26, no. 11 (2020): 1686–1690. 10.1038/s41591-020-1074-2 33046868

[387] S. M. Chang and M. G. Vander Heiden , “Inhibiting Gluttony in Cancer,” Cell Chem Biol 29, no. 3 (2022): 353–355. 35303439 10.1016/j.chembiol.2022.03.004

[388] M. Mueckler and B. Thorens , “The Slc2 (Glut) Family of Membrane Transporters,” Molecular Aspects of Medicine 34, no. 2–3 (2013): 121–138. 23506862 10.1016/j.mam.2012.07.001 PMC4104978

[389] T. Robichaud , A. N. Appleyard , R. B. Herbert , et al., “Determinants of Ligand Binding Affinity and Cooperativity at the Glut1 Endofacial Site,” Biochemistry 50, no. 15 (2011): 3137–3148. 21384913 10.1021/bi1020327 PMC3465710

[390] G. Wang , Y. Lai , X. Chen , et al., “Hexokinase 2 Promotes Tumor Development and Progression,” American Journal of Cancer Research 15, no. 10 (2025): 4499–4515. 41244129 10.62347/ZYNN3077 PMC12616175

[391] F. Baenke , B. Peck , H. Miess , et al., “Hooked on Fat: The Role of Lipid Synthesis in Cancer Metabolism and Tumour Development,” Disease Models & Mechanisms 6, no. 6 (2013): 1353–1363. 24203995 10.1242/dmm.011338 PMC3820259

[392] J. A. Menendez and R. Lupu , “Fatty Acid Synthase and the Lipogenic Phenotype in Cancer Pathogenesis,” Nature Reviews Cancer 7, no. 10 (2007): 763–777. 17882277 10.1038/nrc2222

[393] C. Liu , M. Chikina , R. Deshpande , et al., “Treg Cells Promote the Srebp1‐Dependent Metabolic Fitness of Tumor‐Promoting Macrophages via Repression of Cd8(+) T Cell‐Derived Interferon‐Γ,” Immunity 51, no. 2 (2019): 381–397. e6. 31350177 10.1016/j.immuni.2019.06.017 PMC6703933

[394] Q. Zhang , H. Wang , C. Mao , et al., “Fatty Acid Oxidation Contributes to Il‐1β Secretion in M2 Macrophages and Promotes Macrophage‐Mediated Tumor Cell Migration,” Molecular Immunology 94 (2018): 27–35. 29248877 10.1016/j.molimm.2017.12.011 PMC5801116

[395] F. Hossain , A. A. Al‐Khami , D. Wyczechowska , et al., “Inhibition of Fatty Acid Oxidation Modulates Immunosuppressive Functions of Myeloid‐Derived Suppressor Cells and Enhances Cancer Therapies,” Cancer Immunology Research 3, no. 11 (2015): 1236–1247. 26025381 10.1158/2326-6066.CIR-15-0036 PMC4636942

[396] V. Cruzat , M. Macedo Rogero , K. Noel Keane , et al., “Glutamine: Metabolism and Immune Function, Supplementation and Clinical Translation,” Nutrients 10, no. 11 (2018), 10.3390/nu10111564. PMC6266414 30360490

[397] B. J. Altman , Z. E. Stine , and C. V. Dang , “From Krebs to Clinic: Glutamine Metabolism to Cancer Therapy,” Nature Reviews Cancer 16, no. 11 (2016): 749. 10.1038/nrc.2016.114 28704361

[398] A. K. Jha , S. C. Huang , A. Sergushichev , et al., “Network Integration of Parallel Metabolic and Transcriptional Data Reveals Metabolic Modules That Regulate Macrophage Polarization,” Immunity 42, no. 3 (2015): 419–430. 25786174 10.1016/j.immuni.2015.02.005

[399] P. S. Liu , H. Wang , X. Li , et al., “Α‐Ketoglutarate Orchestrates Macrophage Activation Through Metabolic and Epigenetic Reprogramming,” Nature Immunology 18, no. 9 (2017): 985–994. 28714978 10.1038/ni.3796

[400] E. M. Palmieri , A. Menga , R. Martín‐Pérez , et al., “Pharmacologic or Genetic Targeting of Glutamine Synthetase Skews Macrophages Toward an M1‐Like Phenotype and Inhibits Tumor Metastasis,” Cell reports 20, no. 7 (2017): 1654–1666. 28813676 10.1016/j.celrep.2017.07.054 PMC5575233

[401] J. M. Matés , F. J. Di Paola , J. A. Campos‐Sandoval , et al., “Therapeutic Targeting of Glutaminolysis as an Essential Strategy to Combat Cancer,” Seminars in Cell & Developmental Biology 98 (2020): 34–43. 31100352 10.1016/j.semcdb.2019.05.012

[402] G. Ma , Z. Zhang , P. Li , et al., “Reprogramming of Glutamine Metabolism and Its Impact on Immune Response in the Tumor Microenvironment,” Cell Communication and Signaling 20, no. 1 (2022): 114. 35897036 10.1186/s12964-022-00909-0 PMC9327201

[403] R. Yadav , A. V. Singh , S. Kushwaha , et al., “Emerging Role of Exosomes as a Liquid Biopsy Tool for Diagnosis, Prognosis & Monitoring Treatment Response of Communicable & Non‐Communicable Diseases,” Indian Journal of Medical Research 159, no. 2 (2024): 163–180. 38577857 10.4103/ijmr.ijmr_2344_22 PMC11050750

[404] S. Li , M. Yi , B. Dong , et al., “The Role of Exosomes in Liquid Biopsy for Cancer Diagnosis and Prognosis Prediction,” International Journal of Cancer 148, no. 11 (2021): 2640–2651. 33180334 10.1002/ijc.33386 PMC8049049

[405] B. Corradetti , D. Gonzalez , I. Mendes Pinto , et al., “Editorial: Exosomes as Therapeutic Systems,” Frontiers in Cell and Developmental Biology 9 (2021): 714743. 34368165 10.3389/fcell.2021.714743 PMC8335480

[406] M. Li , S. Li , C. Du , et al., “Exosomes From Different Cells: Characteristics, Modifications, and Therapeutic Applications,” European Journal of Medicinal Chemistry 207 (2020): 112784. 33007722 10.1016/j.ejmech.2020.112784

[407] A. T. H. Wu , P. Srivastava , V. K. Yadav , et al., “Ovatodiolide, Isolated From Anisomeles Indica, Suppresses Bladder Carcinogenesis Through Suppression of Mtor/Β‐Catenin/Cdk6 and Exosomal Mir‐21 Derived From M2 Tumor‐Associated Macrophages,” Toxicology and Applied Pharmacology 401 (2020): 115109. 32544403 10.1016/j.taap.2020.115109

[408] À. M. Bellmunt , L. López‐Puerto , J. Lorente , et al., “Involvement of Extracellular Vesicles in the Macrophage‐Tumor Cell Communication in Head and Neck Squamous Cell Carcinoma,” PLoS ONE 14, no. 11 (2019): e0224710. 31697737 10.1371/journal.pone.0224710 PMC6837305

[409] K. Trajkovic , C. Hsu , S. Chiantia , et al., “Ceramide Triggers Budding of Exosome Vesicles Into Multivesicular Endosomes,” Science 319 (2008): 5867. 10.1126/science.1153124 18309083

[410] R. Tenchov , J. M. Sasso , X. Wang , et al., “Exosomes─Nature's Lipid Nanoparticles, a Rising Star in Drug Delivery and Diagnostics,” ACS Nano 16, no. 11 (2022): 17802–17846. 36354238 10.1021/acsnano.2c08774 PMC9706680

[411] D. Wu , Q. Chen , X. Chen , et al., “The Blood‐Brain Barrier: Structure, Regulation, and Drug Delivery,” Signal Transduct Target Ther 8, no. 1 (2023): 217. 37231000 10.1038/s41392-023-01481-w PMC10212980

[412] Y. Zhang , Q. Liu , X. Zhang , et al., “Recent Advances in Exosome‐Mediated Nucleic Acid Delivery for Cancer Therapy,” J Nanobiotechnology 20, no. 1 (2022): 279. 35701788 10.1186/s12951-022-01472-z PMC9194774

[413] G. R. Gunassekaran , S. M. Poongkavithai Vadevoo , M. C. Baek , et al., “M1 Macrophage Exosomes Engineered to Foster M1 Polarization and Target the Il‐4 Receptor Inhibit Tumor Growth by Reprogramming Tumor‐Associated Macrophages Into M1‐Like Macrophages,” Biomaterials 278 (2021): 121137. 34560422 10.1016/j.biomaterials.2021.121137

[414] L. Xiao , U. Erb , K. Zhao , et al., “Efficacy of Vaccination With Tumor‐Exosome Loaded Dendritic Cells Combined With Cytotoxic Drug Treatment in Pancreatic Cancer,” Oncoimmunology 6, no. 6 (2017): e1319044. 28680753 10.1080/2162402X.2017.1319044 PMC5486185

[415] B. Laura , C. Marie‐May , W. Jeffrey , et al., “Cessation of Ccl2 Inhibition Accelerates Breast Cancer Metastasis by Promoting Angiogenesis,” Nature 515, no. 7525 (2014): 130–133. 25337873 10.1038/nature13862

[416] R. Kalluri and M. Zeisberg , “Fibroblasts in Cancer,” Nature Reviews Cancer 6, no. 5 (2006): 392–401. 16572188 10.1038/nrc1877

[417] S. E. Kuzet and C. Gaggioli , “Fibroblast Activation in Cancer: When Seed Fertilizes Soil,” Cell and Tissue Research 365, no. 3 (2016): 607–619. 27474009 10.1007/s00441-016-2467-x

[418] H. Yamaguchi and R. Sakai , “Direct Interaction Between Carcinoma Cells and Cancer Associated Fibroblasts for the Regulation of Cancer Invasion,” Cancers (Basel) 7, no. 4 (2015): 2054–2062. 26473929 10.3390/cancers7040876 PMC4695876

[419] P. Zhang , C. Zhang , X. Li , et al., “Immunotherapy for Gastric Cancer: Advances and Challenges,” MedComm–Oncology 3, no. 4 (2024): e92.

[420] D. Hanahan and M. Coussens Lisa , “Accessories to the Crime: Functions of Cells Recruited to the Tumor Microenvironment,” Cancer Cell 21, no. 3 (2012): 309–322. 22439926 10.1016/j.ccr.2012.02.022

[421] A. Ray and P. Cleary Margot , “The Potential Role of Leptin in Tumor Invasion and Metastasis,” Cytokine & Growth Factor Reviews 38 (2017): 80–97. 29158066 10.1016/j.cytogfr.2017.11.002 PMC5720178

[422] E. Sounni Nor and A. Noel , “Targeting the Tumor Microenvironment for Cancer Therapy,” Clinical Chemistry 59, no. 1 (2013): 85–93. 23193058 10.1373/clinchem.2012.185363

[423] S. Jaillon , A. Ponzetta , D. Di Mitri , et al., “Neutrophil Diversity and Plasticity in Tumour Progression and Therapy,” Nature Reviews Cancer 20, no. 9 (2020): 485–503. 32694624 10.1038/s41568-020-0281-y

[424] B. Eruslanov Evgeniy , “Phenotype and Function of Tumor‐Associated Neutrophils and Their Subsets in Early‐Stage Human Lung Cancer,” Cancer Immunology, Immunotherapy 66, no. 8 (2017): 997–1006. 28283697 10.1007/s00262-017-1976-0 PMC5522629

[425] E. Shaul Merav and Z. G. Fridlender , “Cancer‐Related Circulating and Tumor‐Associated Neutrophils–Subtypes, Sources and Function,” The FEBS journal 285 (2018): 4316–4342. 29851227 10.1111/febs.14524

[426] M. A. Giese , L. E. Hind , and A. Huttenlocher , “Neutrophil Plasticity in the Tumor Microenvironment,” The Journal of the American Society of Hematology 133, no. 20 (2019): 2159–2167. 10.1182/blood-2018-11-844548 PMC6524564 30898857

[427] W. Treffers Louise , H. Hiemstra Ida , T. W. Kuijpers , et al., “Neutrophils in Cancer,” Immunological Reviews 273, no. 1 (2016): 312–328. 27558343 10.1111/imr.12444

[428] Y. Zhang , L. Guoqiang , M. Sun , et al., “Targeting and Exploitation of Tumor‐Associated Neutrophils to Enhance Immunotherapy and Drug Delivery for Cancer Treatment,” Cancer Biology & Medicine 17, no. 1 (2020): 32–43. 32296575 10.20892/j.issn.2095-3941.2019.0372 PMC7142839

[429] E. Shaul Merav and Z. G. Fridlender , “Tumour‐Associated Neutrophils in Patients With Cancer,” Nature Reviews Clinical oncology 16, no. 10 (2019): 601–620. 10.1038/s41571-019-0222-4 31160735

[430] A. Ocana , C. Nieto‐Jiménez , A. Pandiella , et al., “Neutrophils in Cancer: Prognostic Role and Therapeutic Strategies,” Molecular Cancer 16 (2017): 1–7. 28810877 10.1186/s12943-017-0707-7 PMC5558711

[431] W. Liu , W. Wang , X. Wang , et al., “Cisplatin‐Stimulated Macrophages Promote Ovarian Cancer Migration via the Ccl20‐Ccr6 Axis,” Cancer Letters 472 (2020): 59–69. 31866467 10.1016/j.canlet.2019.12.024

[432] C. W. Wanderley , D. F. Colon , M. Luiz Joao Paulo , et al., “Paclitaxel Reduces Tumor Growth by Reprogramming Tumor‐Associated Macrophages to an M1 Profile in a Tlr4‐Dependent Manner,” Cancer Research 78 (2018): 5891–5900. 30104241 10.1158/0008-5472.CAN-17-3480

[433] Y. Li , Z. Shen , Z. Chai , et al., “Targeting Ms4a4a on Tumour‐Associated Macrophages Restores Cd8+ T‐Cell‐Mediated Antitumour Immunity,” Gut 72, no. 12 (2023): 2307–2320. 37507218 10.1136/gutjnl-2022-329147 PMC10715532

[434] C. Salvagno , M. Ciampricotti , S. Tuit , et al., “Therapeutic Targeting of Macrophages Enhances Chemotherapy Efficacy by Unleashing Type I Interferon Response,” Nature Cell Biology 21, no. 4 (2019): 511–521. 30886344 10.1038/s41556-019-0298-1 PMC6451630

[435] D. Wolff , C. Cutler , S. J. Lee , et al., “Axatilimab in Recurrent or Refractory Chronic Graft‐Versus‐Host Disease,” New England Journal of Medicine 391 (2024): 1002–1014. 39292927 10.1056/NEJMoa2401537

[436] S. Song , H. Xia , M. Guo , et al., “Role of Macrophage in Nanomedicine‐Based Disease Treatment,” Drug Delivery 28, no. 1 (2021): 752–766. 33860719 10.1080/10717544.2021.1909175 PMC8079019

[437] A. L. Facklam , L. R. Volpatti , and D. G. Anderson , “Biomaterials for Personalized Cell Therapy,” Advanced Materials 32, no. 13 (2020): e1902005. 31495970 10.1002/adma.201902005

[438] A. B. Kuznetsova , E. P. Kolesova , A. Parodi , et al., “Reprogramming Tumor‐Associated Macrophage Using Nanocarriers: New Perspectives to Halt Cancer Progression,” Pharmaceutics 16, no. 5 (2024), 10.3390/pharmaceutics16050636. PMC11124960 38794298

[439] R. Sumitomo , T. Hirai , M. Fujita , et al., “Pd‐L1 Expression on Tumor‐Infiltrating Immune Cells Is Highly Associated With M2 Tam and Aggressive Malignant Potential in Patients With Resected Non‐Small Cell Lung Cancer,” Lung Cancer 136 (2019): 136–144. 31499335 10.1016/j.lungcan.2019.08.023

[440] Y. Yin , B. Liu , Y. Cao , et al., “Colorectal Cancer‐Derived Small Extracellular Vesicles Promote Tumor Immune Evasion by Upregulating Pd‐L1 Expression in Tumor‐Associated Macrophages,” Advanced Science 9, no. 9 (2022): 2102620. 35356153 10.1002/advs.202102620 PMC8948581

[441] P. Hartley Genevieve , C. Lyndah , T. Ammons Dylan , et al., “Programmed Cell Death Ligand 1 (Pd‐L1) Signaling Regulates Macrophage Proliferation and Activation,” Cancer Immunology Research 6, no. 10 (2018): 1260–1273. 30012633 10.1158/2326-6066.CIR-17-0537

[442] Z. Zhang , X. Liu , D. Chen , et al., “Radiotherapy Combined With Immunotherapy: The Dawn of Cancer Treatment,” Signal Transduct Target Ther 7, no. 1 (2022): 258. 35906199 10.1038/s41392-022-01102-y PMC9338328

[443] R. A. Mcmahon , C. D'souza , P. J. Neeson , et al., “Innate Immunity: Looking beyond T‐Cells in Radiation and Immunotherapy Combinations,” Neoplasia 46 (2023): 100940. 37913654 10.1016/j.neo.2023.100940 PMC10637988

[444] , “Correction: Short‐Course Radiotherapy Promotes Pro‐Inflammatory Macrophages via Extracellular Vesicles in Human Rectal Cancer.” Journal for ImmunoTherapy of Cancer 8, no. 2 (2020), 10.1136/jitc-2020-000667corr1. PMC7437887 32817359

[445] R. Evans and P. Alexander , “Role of Macrophages in Tumour Immunity: I. Co‐Operation Between Macrophages and Lymphoid Cells in Syngeneic Tumour Immunity,” Immunology 23, no. 4 (1972): 615. 5084104 PMC1407972

[446] L. Milas , J. Wike , N. Hunter , et al., “Macrophage Content of Murine Sarcomas and Carcinomas: Associations With Tumor Growth Parameters and Tumor Radiocurability,” Cancer Research 47, no. 4 (1987): 1069–1075. 3802091

[447] M. J. Buiting Antoinette and V. Rooijen Nico , “Liposome Mediated Depletion of Macrophages: An Approach for Fundamental Studies,” Journal of Drug Targeting 2, no. 5 (1994): 357–362. 7704479 10.3109/10611869408996810

[448] J. Rogers Michael , S. Gordon , H. L. Benford , et al., “Cellular and Molecular Mechanisms of Action of Bisphosphonates,” Cancer: Interdisciplinary International Journal of the American Cancer Society 88, no. S12 (2000): 2961–2978. 10.1002/1097-0142(20000615)88:12+<2961::aid-cncr12>3.3.co;2-c 10898340

[449] J. R. Ross , Y. Saunders , P. M. Edmonds , et al., “A Systematic Review of the Role of Bisphosphonates in Metastatic Disease,” Health Technology Assessment (Winchester, England) 8, no. 4 (2004). III‐. 10.3310/hta8040 14960258

[450] S. M. Zeisberger , B. Odermatt , C. Marty , et al., “Clodronate‐Liposome‐Mediated Depletion of Tumour‐Associated Macrophages: A New and Highly Effective Antiangiogenic Therapy Approach,” British Journal of Cancer 95, no. 3 (2006): 272–281. 16832418 10.1038/sj.bjc.6603240 PMC2360657

[451] E. Giraudo , M. Inoue , and D. Hanahan , “An Amino‐Bisphosphonate Targets Mmp‐9–Expressing Macrophages and Angiogenesis to Impair Cervical Carcinogenesis,” The Journal of Clinical Investigation 114, no. 5 (2004): 623–633. 15343380 10.1172/JCI22087 PMC514591

[452] P. Seiler , P. Aichele , B. Odermatt , et al., “Crucial Role of Marginal Zone Macrophages and Marginal Zone Metallophils in the Clearance of Lymphocytic Choriomeningitis Virus Infection,” European Journal of Immunology 27, no. 10 (1997): 2626–2633. 9368619 10.1002/eji.1830271023

[453] W. Tyner Jeffrey , O. Uchida , N. Kajiwara , et al., “Ccl5‐Ccr5 Interaction Provides Antiapoptotic Signals for Macrophage Survival during Viral Infection,” Nature Medicine 11, no. 11 (2005): 1180–1187. 10.1038/nm1303 PMC6322907 16208318

[454] K. Mulder , A. A. Patel , T. Kong Wan , et al., “Cross‐Tissue Single‐Cell Landscape of Human Monocytes and Macrophages in Health and Disease,” Immunity 54, no. 8 (2021): 1883–1900. e5. 34331874 10.1016/j.immuni.2021.07.007

[455] M. Nahrendorf and F. K. Swirski , “Abandoning M1/M2 for a Network Model of Macrophage Function,” Circulation Research 119, no. 3 (2016): 414–417. 27458196 10.1161/CIRCRESAHA.116.309194 PMC4965179

[456] D. M. Mosser and J. P. Edwards , “Exploring the Full Spectrum of Macrophage Activation,” Nature Reviews Immunology 8, no. 12 (2008): 958–969. 10.1038/nri2448 PMC2724991 19029990

[457] A. Giladi , F. Paul , Y. Herzog , et al., “Single‐Cell Characterization of Haematopoietic Progenitors and Their Trajectories in Homeostasis and Perturbed Haematopoiesis,” Nature Cell Biology 20, no. 7 (2018): 836–846. 29915358 10.1038/s41556-018-0121-4

[458] D. A. Lawson , K. Kessenbrock , R. T. Davis , et al., “Tumour Heterogeneity and Metastasis at Single‐Cell Resolution,” Nature Cell Biology 20, no. 12 (2018): 1349–1360. 30482943 10.1038/s41556-018-0236-7 PMC6477686

[459] X. Ren , L. Zhang , Y. Zhang , et al., “Insights Gained From Single‐Cell Analysis of Immune Cells in the Tumor Microenvironment,” Annual Review of Immunology 39, no. 1 (2021): 583–609. 10.1146/annurev-immunol-110519-071134 33637019

[460] M. A. Ruo‐Yu , B. Annabel , and Q. Bin‐Zhi , “Macrophage Diversity in Cancer Revisited in the Era of Single‐Cell Omics,” Trends in Immunology 43, no. 7 (2022): 546–563. 35690521 10.1016/j.it.2022.04.008

[461] C. A. Gomez‐Roca , P. A. Cassier , A. Italiano , et al., “Phase I Study of Rg7155, a Novel Anti‐Csf1r Antibody, in Patients With Advanced/Metastatic Solid Tumors,” American Society of Clinical Oncology (2015).

[462] Y. Zhu , L. Knolhoff Brett , M. A. Meyer , et al., “Csf1/Csf1r Blockade Reprograms Tumor‐Infiltrating Macrophages and Improves Response to T‐Cell Checkpoint Immunotherapy in Pancreatic Cancer Models,” Cancer Research 74, no. 18 (2014): 5057–5069. 25082815 10.1158/0008-5472.CAN-13-3723 PMC4182950

[463] Y. Yang , H. E. Wu , Y. Yang , et al., “Dual Blockade of Cd47 and Cd24 Signaling Using a Novel Bispecific Antibody Fusion Protein Enhances Macrophage Immunotherapy,” Molecular Therapy‐Oncolytics 31 (2023): 100747. 38046893 10.1016/j.omto.2023.100747 PMC10689933

[464] H. June Carl , S. O'connor Roddy , U. Kawalekar Omkar , et al., “Car T Cell Immunotherapy for Human Cancer,” Science 359, no. 6382 (2018): 1361–1365. 29567707 10.1126/science.aar6711

[465] L. Beatty Gregory , R. Haas Andrew , V. Maus Marcela , et al., “Mesothelin‐Specific Chimeric Antigen Receptor Mrna‐Engineered T Cells Induce Antitumor Activity in Solid Malignancies,” Cancer Immunology Research 2, no. 2 (2014): 112–120. 24579088 10.1158/2326-6066.CIR-13-0170 PMC3932715

[466] D. Marin , Y. E. Li , R. Basar , et al., “Safety, Efficacy and Determinants of Response of Allogeneic Cd19‐Specific Car‐Nk Cells in Cd19+ B Cell Tumors: A Phase 1/2 Trial,” Nature Medicine 30, no. 3 (2024): 772–784. 10.1038/s41591-023-02785-8 PMC10957466 38238616

[467] R. H. Andrew , L. Tanyi Janos , H. O'hara Mark , et al., “Phase I Study of Lentiviral‐Transduced Chimeric Antigen Receptor‐Modified T Cells Recognizing Mesothelin in Advanced Solid Cancers,” Molecular Therapy 27, no. 11 (2019): 1919–1929. 31420241 10.1016/j.ymthe.2019.07.015 PMC6838875

[468] H. Yu , E. Sotillo , C. Harrington , et al., “Repeated Loss of Target Surface Antigen After Immunotherapy in Primary Mediastinal Large B Cell Lymphoma,” American Journal of Hematology 92, no. 1 (2016): E11. 27779774 10.1002/ajh.24594 PMC8620941

[469] M. O'rourke Donald , P. Nasrallah MacLean , A. Desai , et al., “A Single Dose of Peripherally Infused Egfrviii‐Directed Car T Cells Mediates Antigen Loss and Induces Adaptive Resistance in Patients With Recurrent Glioblastoma,” Science Translational Medicine 9, no. 399 (2017): eaaa0984. 28724573 10.1126/scitranslmed.aaa0984 PMC5762203

[470] M. Poorebrahim , J. Melief , Y. Pico De Coaña , et al., “Counteracting Car T Cell Dysfunction,” Oncogene 40, no. 2 (2021): 421–435. 33168929 10.1038/s41388-020-01501-x PMC7808935

[471] A. Reiss Kim , G. Angelos Mathew , and E. C. Dees , “Car‐Macrophage Therapy for Her2‐Overexpressing Advanced Solid Tumors: A Phase 1 Trial,” Nature Medicine (2025): 1–12. 10.1038/s41591-025-03495-z 39920391

[472] W. Ma and C. Jamieson , “Chimeric Antigen Receptor‐Macrophage Therapy Enters the Clinic: The First‐in‐Human Trial for Her2(+) Solid Tumors,” MedComm 6, no. 9 (2025): e70374. 40895191 10.1002/mco2.70374 PMC12390764

[473] Y. Chen , Z. Yu , X. Tan , et al., “Car‐Macrophage: A New Immunotherapy Candidate Against Solid Tumors,” Biomedicine & Pharmacotherapy 139 (2021): 111605. 33901872 10.1016/j.biopha.2021.111605

[474] A. Wróblewska , A. Szczygieł , B. O. Szermer‐Olearnik , et al., “Macrophages as Promising Carriers for Nanoparticle Delivery in Anticancer Therapy,” International Journal of Nanomedicine (2023): 4521–4539. 37576466 10.2147/IJN.S421173 PMC10422973

[475] D. Mayya Alexandrovna , T. Sergey Yurjevich , R. Vladimir Alexandrovich , et al., “Boron Neutron Capture Therapy: Current Status and Future Perspectives,” Cancer communications 40, no. 9 (2020): 406–421. 32805063 10.1002/cac2.12089 PMC7494062

[476] H. Cao , Z. Dan , X. He , et al., “Liposomes Coated With Isolated Macrophage Membrane Can Target Lung Metastasis of Breast Cancer,” ACS Nano 10, no. 8 (2016): 7738–7748. 27454827 10.1021/acsnano.6b03148

[477] K. Kuerban , X. Gao , H. Zhang , et al., “Doxorubicin‐Loaded Bacterial Outer‐Membrane Vesicles Exert Enhanced Anti‐Tumor Efficacy in Non‐Small‐Cell Lung Cancer,” Acta pharmaceutica Sinica B 10, no. 8 (2020): 1534–1548. 32963948 10.1016/j.apsb.2020.02.002 PMC7488491

[478] J. Fu , D. Wang , D. Mei , et al., “Macrophage Mediated Biomimetic Delivery System for the Treatment of Lung Metastasis of Breast Cancer,” Journal of Controlled Release 204 (2015): 11–19. 25646783 10.1016/j.jconrel.2015.01.039

[479] R. Kalluri and V. S. Lebleu , “The Biology, Function, and Biomedical Applications of Exosomes,” Science 367, no. 6478 (2020), 10.1126/science.aau6977. PMC7717626 32029601

[480] T. Wu , Y. Liu , Y. Cao , et al., “Engineering Macrophage Exosome Disguised Biodegradable Nanoplatform for Enhanced Sonodynamic Therapy of Glioblastoma,” Advanced Materials 34, no. 15 (2022): e2110364. 35133042 10.1002/adma.202110364

[481] S. Rayamajhi , T. D. T. Nguyen , R. Marasini , et al., “Macrophage‐Derived Exosome‐Mimetic Hybrid Vesicles for Tumor Targeted Drug Delivery,” Acta Biomaterialia 94 (2019): 482–494. 31129363 10.1016/j.actbio.2019.05.054

